# Target-dependent routing of sensory information in the olfactory system

**DOI:** 10.1038/s42003-026-11071-5

**Published:** 2026-10-07

**Authors:** Kim Le-Jahns, Siran Sireci, Jan Mayland, Franziska Richter, Pablo Chamero, Martin K. Schwarz, Daniela Brunert, Markus Rothermel

**Affiliations:** 1 Department of Chemosensation, RG Neuromodulation, Institute for Biology II, RWTH Aachen University, Aachen, Germany; 2 Institute of Physiology, RG Neurophysiology and Optogenetics, Medical Faculty, Otto-von-Guericke-University, Magdeburg, Germany; 3 Institute for Physiology and Cell Biology, University of Veterinary Medicine, Hanover, Germany; 4 Department of Pharmacology, Toxicology, and Pharmacy, University of Veterinary Medicine, Hanover, Germany; 5 Center for Systems Neuroscience Hanover (ZSN), Hanover, Germany; 6 Laboratoire de Physiologie de la Reproduction et des Comportements, INRAE-CNRS- University of Tours, Nouzilly, France; 7 Institute for Experimental Epileptology and Cognition Research, University of Bonn Medical Faculty, Bonn, Germany

**Keywords:** Olfactory bulb, Sensory processing

## Abstract

Parallel processing, the ability to split the information content of sensory stimuli and handle them simultaneously, is essential for sensory perception in many systems. Mitral (MCs) and tufted cells (TCs) in the olfactory bulb (OB), with their separate but overlapping projection areas, provide an ideal anatomical basis for parallel processing. Whether they convey different information to distinct areas of the olfactory cortex remains unclear. Here, we separated mouse OB output neurons by innervation area using retrograde tracing from the anterior olfactory nucleus (AON) or the anterior piriform cortex (APC). In vivo 2-photon calcium imaging in the OB revealed projection area-dependent differences, with APC-projecting neurons coding more strongly for odor concentration and more selectively for odor identity. This separation became more pronounced when differentially traced populations were split by cell type, with APC TCs displaying the strongest concentration-dependent changes in response and AON TCs showing very promiscuous odor tuning. Purely cell-type-derived properties did not explain the projection target-dependent differences in odor representation, as both mitral and tufted cells showed similar changes, and SVM classifier analysis demonstrated that classification by projection target outperformed classification by cell type. Our data suggests a prominent projection-defined separation of sensory information in the OB.

**Figure:**
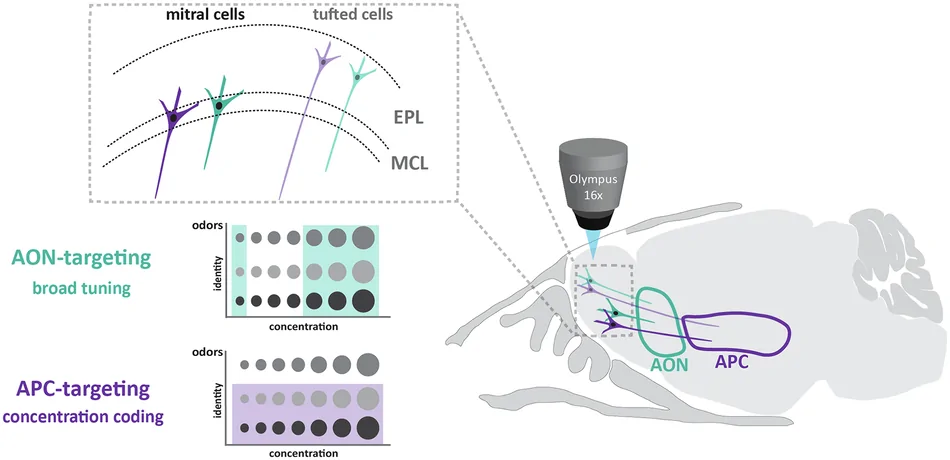

## Introduction

In a constantly changing environment, animals rely on rapid sensory uptake and efficient processing to respond adequately. In the brain, the rapid processing of complex sensory stimuli is facilitated by parallel processing, which divides stimulus features into distinct channels, allowing computations to be performed simultaneously rather than sequentially. This principle is well established for the auditory, somatosensory, and visual systems^1–3^. Since chemical features are immensely complex, parallel processing has been postulated to be especially advantageous for the olfactory system^4–6^. So far, only a few studies have investigated this in detail.

The olfactory system is well-equipped for the parallel processing of sensory information. Olfactory bulb (OB) output neurons project to anatomically and functionally diverse olfactory cortical regions, including the olfactory tubercle (OT), anterior olfactory nucleus, piriform and lateral entorhinal cortex, and the amygdala. While the cortical amygdala has been linked to innate, odor-driven behaviors^7^, the piriform cortex (PC) is implicated in odorant identity coding^8,9^, and olfactory memory^10,11^, the AON in storing episodic odor memories^12,13^, control of food intake^14^ and social odor processing^15–20^ and the OT is thought to be responsible for integrating sensory information and information about motivational states to guide affective and behavioral responses^21^. Accordingly, neuronal recordings from these different cortical areas show area-specific response properties, such as odor selectivity, integration of components in an odorant mixture, and experience-dependent plasticity^20,22–29^. While frequently treated as a single population of neurons, mitral (MCs) and tufted cells (TCs), the two principal OB output cells, target different areas in the olfactory cortex and are excellent candidates to initiate parallel processing in olfaction^6,30–33^. TCs predominantly innervate anterior targets such as the AON, OT, and the anterior part of the PC, while MCs project to all major olfactory cortical areas^32–38^. MCs and TCs also exhibit distinct odor response properties^31,39–43^. TCs have a higher firing rate^30^, lower odor sensitivity threshold^33,44–46^, shorter onset latencies^33^, and broader odor responsiveness^32,45,46^ compared to MCs. Inhalation-coupled activity is also processed differentially in these cell types, with TCs showing faster^33^, stronger^47^, and more reliable^48^ sniff-linked responses. It is unknown whether distinct activity patterns in olfactory cortical areas are mainly derived from local recurrent networks that shape incoming information^49^, or whether olfactory information is already segregated at the level of the OB, depending on the targeted olfactory cortical area.

Here, we analyzed the response properties of OB output neurons defined by their cortical projection. To investigate differences due to cortical projection rather than pure differences between MCs and TCs, we retrogradely traced OB inputs from two olfactory cortex areas receiving input from both OB output neuron types: the anterior olfactory nucleus (AON) and the anterior piriform cortex (APC). This approach enabled the separation of AON- and APC-projecting output neurons. At the population level, AON-projecting cells displayed stronger inhalation-linked amplitudes than APC-projecting cells. AON-projecting single cells were less odor selective and showed less concentration variance than APC-innervating cells. Further subdividing target-defined neurons by cell type, i.e., MCs and TCs, revealed that AON-projecting TCs showed the broadest response and APC-projecting TCs the strongest concentration-variant odor responses. Analyzing inhalation-evoked transients displayed a stronger sniff-modulation in AON projecting TCs, and classifier analysis based on inhalation transients showed a higher classification performance for AON vs. APC than for MCs vs. TCs.

In summary, our findings suggest that OB output is not only segregated by MCs and TCs. Instead, olfactory information at the OB level is segregated depending on the innervation target, thereby setting the stage for fast and efficient parallel processing in higher olfactory cortical areas.

## Results

### Target-defined OB output streams show similar spatiotemporal properties in widefield imaging

**Fig. 1:**
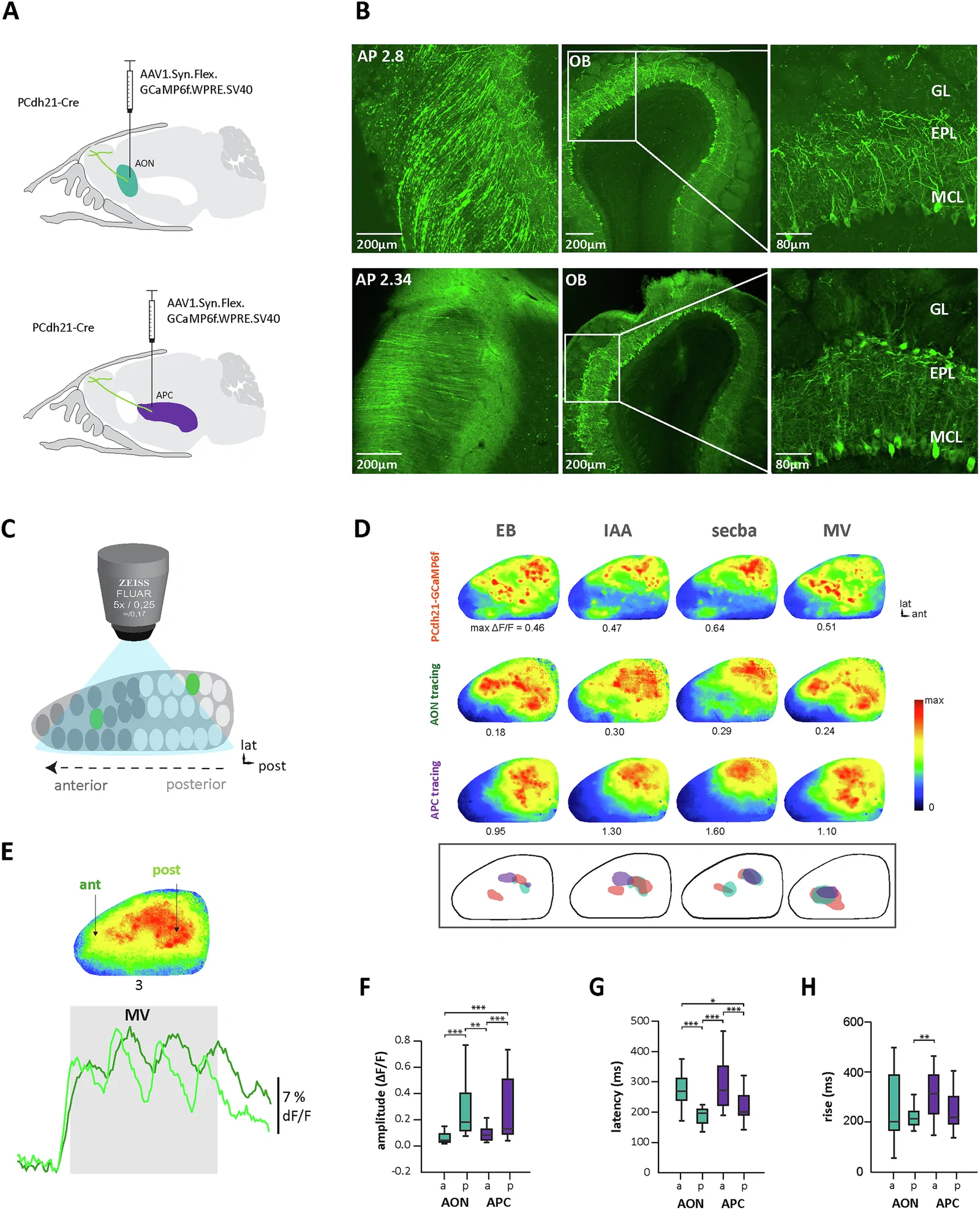
Target-defined OB output streams exhibit similar properties in widefield imaging. **A** Schematics of the tracing approach. AAV constructs were injected into AON or APC to separate MTCs projecting to the respective target. **B** Histological sections showing GCaMP6f expression in the lateral olfactory tract (LOT) (left) and OB (middle and right) for AON- (top, AP 2.8) and APC-traced animals (bottom, AP 2.34). **C** Schematic of the WF imaging approach. Imaging was performed in Pcdh21-GCaMP6f mice expressing GCaMP6f transgenically in ORN, ET and MTCs or virus-injected mice, expressing GCaMP6f in target-defined output neurons. **D** Topographic response maps for different odors from Pcdh21GCaMP6f, AON- (*n* = 5 mice), and APC-traced mice (*n* = 6). All maps are normalized to their own maximum; maximal Δ*F*/*F* values are given below each map. Activity maps in animals from the viral tracing approach exhibit a more prominent diffuse component than the activation of distinct foci observed in transgenic mice, likely reflecting pre- and postsynaptic activity of OSNs and output neurons. MV: methyl valerate, secba: sec-butyl acetate, IAA: isoamyl acetate, EB: ethyl butyrate. Bottom: Contour maps show similar topography across odorants. **E** Representative topographic odor response (top) and trace (bottom) from anterior and posterior parts of the OB of traced mice. **F**–**H** Inhalation-linked amplitudes, latencies, and rise times, respectively, for anterior and posterior parts of AON- and APC-projecting neurons (*n* = 5 mice for AON-traced, *n* = 6 for APC-traced mice, 28 and 32 cells, respectively). Boxplots display the median with horizontal lines, and the edges indicate the 25th and 75th percentiles, with maximum and minimum values plotted, excluding outliers. One-way ANOVA was performed for statistical analysis: **F** *p* < 0.001, Tukey’s HSD Post-Hoc comparisons AON a.-AON p. *p* < 0.001, AON a.-APC p. *p* < 0.001, AON p.-APC a. *p* = 0.004, APC a.-APC p. *p* < 0.001; **G** *p* < 0.001, Tukey’s HSD Post-Hoc comparisons AON a.-AON p. *p* < 0.001, AON a.-APC p. *p* = 0.012, AON p.-APC a. *p* < 0.001, APC a.-APC p. *p* < 0.001; **H** *p* = 0.12, Tukey’s HSD Post-Hoc comparisons AON p.-APC a. *p* = 0.008.

The principal output neurons of the OB, mitral and tufted cells (MTCs), can be differentiated by cell depth^50–52^ as well as by genetic markers^53^, and anatomical studies have demonstrated that they project to overlapping areas of the anterior olfactory cortex^32,33,54^. To investigate how far the olfactory cortex might receive specialized information from the OB, we labeled MTCs depending on their area of innervation^55,56^. To label OB output based on their axonal targets in the olfactory cortex, we used an AAV-mediated retrograde tracing approach in Pcdh21-Cre mice, a mouse line expressing Cre-recombinase selectively in the two types of olfactory output neurons, MCs and TCs, as well as external tufted cells within the OB^56,57^. This tracing approach relies on the ability of most AAV serotypes for retrograde uptake^55,58–63^. We tested an AAV construct encoding the calcium reporter GCaMP6f^64^ for its retrograde transport abilities. We chose to trace from the AON and APC (Fig. 1A) since these olfactory cortical areas receive overlapping input from both types of olfactory output neurons^33,34,36,65^. Injecting AAV-GCaMP6f (*n* = 7 animals for APC and 11 animals for AON) into the AON or APC resulted in robust retrograde labeling of OB neurons for both injection sites (Fig. 1B, middle and right). No labeled cell somata were detectable in the olfactory cortices injected with the AAV (Fig. 1B left), as cortical cells did not express Cre-recombinase. This resulted in a strict retrograde labeling of OB output neurons without additional labeling of top-down fibers from the AON/APC to the OB. To confirm adequate targeting of AON and APC we performed histological reconstruction of tracing sites and needle tracts (Supplementary Fig. 1). As expected, MTCs traced from APC and AON were distributed along the dorsoventral and rostrocaudal OB axis (Fig. 1B, middle). For both injection sites, labeled OB cell somata were located in the mitral cell layer (MCL) and external plexiform layer (EPL), with dendrites reaching into the glomerular layer (GL) (Fig. 1B, right). These data confirm AAV-GCaMP6f as a suitable retrograde tracing tool^66^.

Whereas the spatial organization of odorant-evoked activity patterns has been characterized for presynaptic olfactory sensory neuron (OSN) inputs^67–71^ as well as different populations of postsynaptic OB neurons^43,56,66,72,73^, how far the OB output activity depends on the MTC projection area remains elusive. Since AON and APC serve different functions in odor processing^20,22^, we speculated that differences between AON- and APC-projecting MTCs might already be observable using epifluorescence OB population imaging. For in vivo widefield calcium imaging experiments, mice were anesthetized with pentobarbital, and stimulus sampling was controlled using an artificial inhalation paradigm^67,74,75^. Animals were head-fixed in a stereotaxic device, and epifluorescence signals were acquired through the thinned bone overlying both olfactory bulbs.

To investigate whether MTCs projecting to AON/APC can code distinct odorant features, we recorded the odorant-evoked population-level activity of GCaMP6f-expressing, target-defined output neurons in the olfactory bulb (Fig. 1C). We observed no significant difference in peak odorant-evoked GCaMP6f signals in APC and AON output neuron populations, ranging from 24.5% to 129.7% ∆*F*/*F* and 29.2% to 129.9% ∆*F*/*F* (*n* = 30 ROIs, 5 animals for APC and *n* = 50 ROIs, 6 animals for AON), respectively.

We compared odorant-evoked maps obtained from AON- and APC-traced MTCs to maps from PCdh21-GCaMP6f mice that transgenically expressed GCaMP6f in all MCs, TCs, external tufted cells, and OSNs axon terminals^56^. To investigate the spatial odor representations in MTC populations innervating AON or APC, we topographically mapped odorant-evoked GCaMP6f signals across the dorsal OB for four odorants: EB, IAA, secba, and MV (for abbreviations see “Material and methods”). Odorant-evoked response maps with topographies spanning the entire dorsal OB for the AON- and APC-traced OB neuronal populations were similar to those derived from transgenic, homogeneously expressing PCdh21-GCaMP6f mice (Fig. 1D). In order to explore potential differences in topographic response maps for different odors, consensus topographies (contour plots)^68^ were calculated and compared between the virally traced or transgenic mice^68^. Each map was thresholded at 40% of the maximal amplitude, normalized, and responses to the same odorant were averaged across preparations to assess whether odorants evoked similar patterns across the dorsal surface of the OB. Contour plots show consensus topographies, i.e., they reflect the most reliably activated areas across several animals. Odorants evoked responses with similar topography across the dorsal OB for all three conditions (AON-, APC-virally traced, and transgenic), with peak responses in overlapping domains for each odorant. Activated domains highly overlapped for each odorant (Fig. 1D, lower panel, contour plots). Topographically similar odor maps and the location of labeled neurons across all outer OB layers indicate that APC- and AON-traced neurons are spatially intermingled throughout the OB.

Compared to PCdh21-GCaMP6f mice, maps of AON- or APC-traced MTCs displayed a more noticeably diffuse component, as was expected from previous publications^56^ (Fig. 1D, middle and lower rows), which has been speculated to be caused by the exclusive expression of GCaMP in mitral and tufted cells with their wide dendritic arborization. We measured the spatial contrast of epifluorescence GCaMP6f signals mapped across the dorsal OB for the same set of odorants using autocorrelation analysis (see “Materials and Methods”^56,68^). Autocorrelation widths derived from AON- or APC-traced response maps were not significantly different from each other (APC-traced: 158.5 ± 32.1 µm, *n* = 5 mice; AON-traced: 157.7 ± 31.6 µm, *n* = 6 mice; ANOVA, Tukey’s Honestly Significant Difference (HSD) Post-Hoc comparisons, *p* = 0.992), suggesting a similar diffuse component. In contrast, response maps imaged from PCdh21-GCaMP6f mice consisted primarily of discrete glomerular foci, as expected from the expression of Pcdh21 in presynaptic OSN axons, and a much weaker diffuse signal (Fig. 1D, upper row), resulting in significantly smaller autocorrelation widths (PCdh21-GCaMP6f: 87.1 ± 21.7 µm, *n* = 3 mice, ANOVA, Tukey’s HSD Post-Hoc comparisons, *p* < 0.01) similar to previous publications^56^. The significantly higher diffuse signal in both AON- and APC-traced response maps likely stems from the predominant expression of GCaMP6f in the primary and secondary dendrites of OB output neurons.

We also compared the signal strength and kinetics of inhalation-linked dynamics between AON- and APC-traced MTCs. Inhalation-locked modulation was evident for both traced cell populations and was stronger, and kinetics were faster in the posterior-lateral than the anterior-medial part of the OB (Fig. 1E), as shown previously for population signals in the OB^56,74^.

A custom algorithm that fits the optical signals to a double sigmoid function^56,76^ was used to calculate inhalation-linked amplitudes, latencies, and rise times of either anterior (ant.) or posterior (post.) located regions of interest (ROIs) in epifluorescence imaging experiments. We detected significant differences in inhalation-linked amplitudes (Fig. 1F, AON ant. 8.0 ± 7.2% Δ*F*/*F*; AON post. 25.0 ± 18.2% Δ*F*/*F*; APC ant. 10.9 ± 8.8% Δ*F*/*F*; APC post. 27.0 ± 22.7% Δ*F*/*F*; ANOVA *p* < 0.001, Tukey’s HSD Post-Hoc comparisons AON ant. vs. AON post. *p* < 0.001, AON ant. vs. APC ant. *p* = 0.891, AON ant. vs. APC post. *p* < 0.001, AON post. vs. APC ant. *p* = 0.004, AON post. vs. APC post. *p* = 0.964, APC ant. vs. APC post. *p* < 0.001) and latencies (Fig. 1G, AON ant. 279 ± 61 ms; AON post. 188 ± 27 ms; APC ant. 295 ± 83 ms; APC post. 225 ± 74 ms; ANOVA *p* < 0.001, Tukey’s HSD Post-Hoc comparisons AON ant. vs. AON post. *p* < 0.001, AON ant. vs. APC ant. *p* = 0.776, AON ant. vs. APC post. *p* = 0.012, AON post. vs. APC ant. *p* < 0.001, AON post. vs. APC post. *p* = 0.142, APC ant. vs. APC post. *p* < 0.001) comparing anterior and posterior-located ROIs of the traced neuronal population, similar to what has been shown previously for different OB neuron types^56,74^. Differences in rise time (Fig. 1H, AON ant. 261 ± 124 ms; AON post. 227 ± 58 ms; APC ant. 304 ± 94 ms; APC post. 250 ± 76 ms; ANOVA *p* = 0.012, Tukey’s HSD Post-Hoc comparisons AON ant. vs. AON post. *p* = 0.494, AON ant. vs. APC ant. *p* = 0.272, AON ant. vs. APC post. *p* = 0.965, AON post. vs. APC ant. *p* = 0.008, AON post. vs. APC post. *p* = 0.757, APC ant. vs. APC post. *p* = 0.089) were only significant between anterior-located APC-traced and posterior-located AON-traced ROIs. Comparing anterior and posterior regions between APC- and AON-traced MTCs did not yield significantly different results (*p* = 0.891 and 0.964 for amplitudes, *p* = 0.272 and 0.757 for latencies), indicating that epifluorescence odor maps and population kinetics do not differ between AON- and APC-projecting populations.

### AON-traced MTCs show a larger fraction of responsive cells

**Fig. 2:**
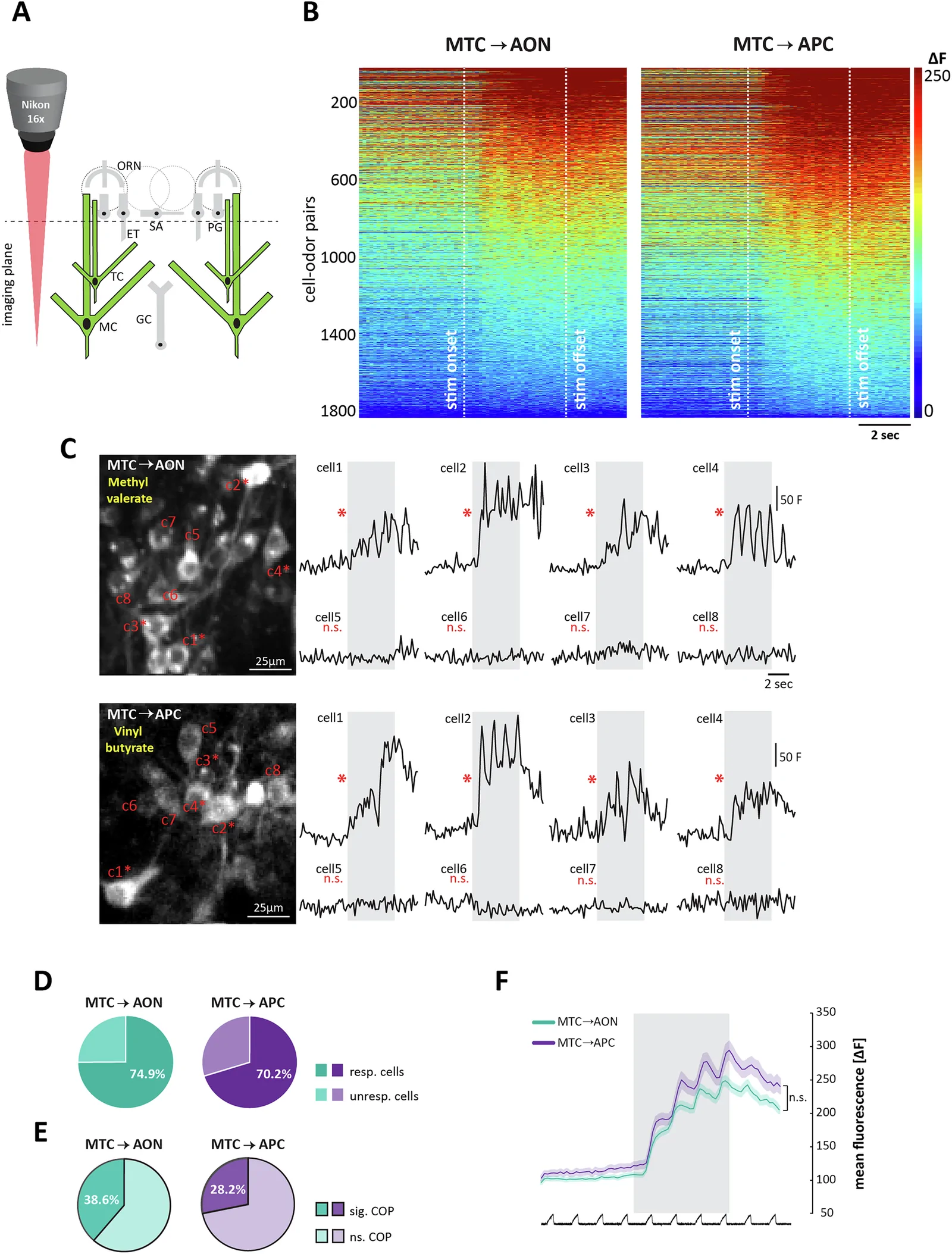
AON-traced MTCs show a larger fraction of responsive cells. **A** Schematic of the 2-photon imaging approach. **B** Pseudocolor plot of odorant-evoked GCaMP6f signals of AON- (left) and APC- (right) traced MTCs. Cell-odor pairs (AON-traced: 1816, APC-traced: 1800) are sorted by fluorescence intensity (Δ*F*) during the time of the odor application. Each row represents one cell-odor pair, with time along the horizontal axis. White dashed lines mark the onset and the end of odor application. **C** 2-photon field of view (left) and representative odor responses (right) of labeled OB MTCs of AON- (top row) and APC-traced cells (bottom row). Significant responses are marked with an asterisk. **D** Proportion of responsive and unresponsive cells in AON- and APC-traced MTCs. **E** Proportion of significant cell-odor pairs (COP) in AON- and APC-traced MTCs. **F** Sniff-aligned average mean fluorescence trace of significant odor-responsive AON- (696 COPs) and APC-traced (507 COPs) MTCs. Grey bar marks the odor stimulation. Lines represent mean values with bands as ± SEM. Two-sided Mann–Whitney U test was used for statistical analysis, *p* = 0.186.

Epifluorescence imaging is based on the integrated signal of a cell population. To investigate potential innervation area-dependent differences of MTCs projecting to AON or APC, we used in vivo 2-photon microscopy to visualize individual retrogradely labeled MT cell bodies at a depth ranging from 150 to 500 µm (Fig. 2A). Imaging sites tiled the entire dorsal olfactory bulb surface. Figure 2B depicts odorant-evoked responses recorded from AON- (left) and APC-traced cells (right) in response to a panel of eight monomolecular odors at 1% VP (see “Materials and Methods”) sorted by odor-evoked signal strength. Odorant stimulation primarily evoked increases in fluorescence signals. Significant inhibitory responses were observed only in 2.5% of AON-traced neurons and none in APC-traced neurons. Inhibitory responses are typically smaller than excitatory ones^66,77,78^, and we might not have picked them up with our metrics. Therefore, we decided to report exclusively on the increase in cellular activity upon odorant stimulation, but we cannot exclude that other distinctions between traced populations might be found in inhibitory responses. Due to the low number of inhibitory responses, further analysis was conducted only on excitatory cell-odor pairs (COPs). Compared to APC-traced MTCs, a slightly higher fraction of AON-traced MTCs responded to at least one odor in a panel (Fig. 2D, AON-traced: 74.9%, 170 out of 225 cells; APC-traced: 70.2%, 158 out of 227 cells, *n* = 5 mice each, χ2 (3) = 0.88, *p* = 0.82). However, when calculated on a cell-odor basis, AON-traced MTCs exhibited a significantly larger fraction of significant responses compared to APC-traced cells (AON-traced: 38.6% significant cell-odor pairs, 696 out of 1816, APC-traced: 28.2% significant cell-odor pairs, 507 out of 1800; χ2 (3) = 42.45, *p* < 0.001, Fig. 2E). In general, the moderate number of COPs depicting significant odor responses for both populations points to an adequate odor concentration used in these experiments.

The fluorescence changes of excitatory responses ranged from 31.5 to 3542.1 Δ*F* for APC-traced and 36.0–1914.6 Δ*F* for AON-traced neurons (mean 247.9 ± 5.2 and 266.1 ± 4.3, respectively). Response kinetics varied considerably within the population of AON- and APC-traced cells (Fig. 2C). Averaged fluorescence traces of all significant odor-responsive AON- or APC-traced MTCs (Fig. 2F) display nearly identical baseline values, while showing slightly, but not significantly, higher maximal amplitudes for APC-traced MTCs (baseline 118.16 ± 70.84 (mean ± SD) AON-MTCs; 119.09 ± 79.59 APC-MTCs, *p* = 0.85, Mann–Whitney U test; amplitudes 337.22 ± 190.17 (mean ± SD) AON-MTCs; 382.70 ± 292.05 APC-MTCs, *p* = 0.55, Mann–Whitney U test). The area under the curve (AUC) was also similar for AON- and APC-traced cells (AON-traced AUC 12,370 ± 7437 (mean ± SD); APC-traced AUC 13,990 ± 11,680; *p* = 0.186, Mann–Whitney U test), arguing that marker protein expression is similar for both target-defined OB output populations.

### MTC odor selectivity: AON-traced cells show reduced specificity

**Fig. 3:**
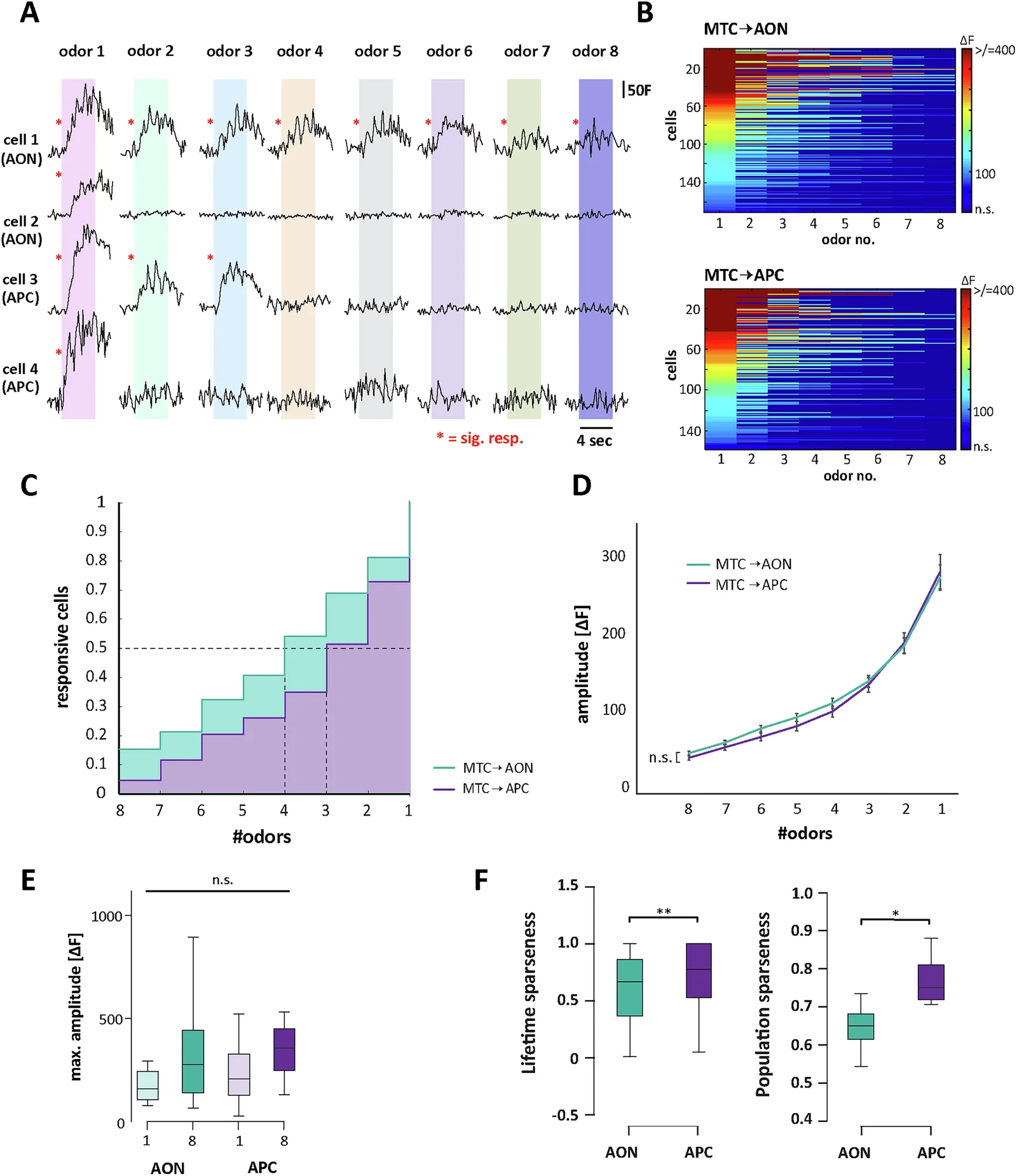
AON-traced MTCs exhibit lower selectivity than APC-traced MTCs. **A** Representative calcium transients of AON- and APC-traced MTCs stimulated with multiple odorants at 1% VP. Colored bars indicate odor stimulation. **B** Population response plot of AON- (top, 170 COPs) and APC- (bottom, 158 COPs) traced MTCs. Cells that showed at least one significant response were included. Cells were sorted by odor response amplitude (Δ*F*) in their respective strongest odor response. **C** Cumulative probability plot indicating the fraction of responsive cells. The probability of reacting to more odors is markedly higher in AON-traced MTCs. **D** Significant maximum odor response amplitudes (Δ*F*) of AON- (*n* = 5) and APC-traced (*n* = 5) cells ranked individually for COP from the weakest to the strongest response (mean ± SEM). All cells with at least one significant odor response were included. **E** Mean responses (Δ*F*) of AON- and APC-traced cells that reacted exclusively to 1 odorant (AON: 33 cells, APC: 43 cells) and those that reacted to all presented odors (AON: 25 cells, APC: 7 cells) (*n* = 5 each). **F** Lifetime and population sparseness analysis for AON- and APC-traced MTCs (*n* = 5 each). Boxplots display the median with horizontal lines, and the edges indicate the 25th and 75th percentiles, with maximum and minimum values plotted, excluding outliers. Statistical analysis for (**E**) was performed with one-way ANOVA (n.s.: not significant). For lifetime and population sparseness analyses in (**F**), two-sided Mann–Whitney U test was employed, and resulted in: *p* = 0.002 and *p* = 0.015, respectively.

Next, we tested for differences in odor selectivity among the two traced output populations. Figure 3A depicts a range of cellular responses obtained for different odorants. Sorting cell-odor pairs by their strongest odor response revealed no relationship between Ca^2+^ signal size and the odor response broadness for both output populations (Fig. 3B), indicating no major impact of signal size on selectivity tuning.

Cumulative probability distribution plots comparing response profiles of AON- and APC-traced MTCs (Fig. 3C) revealed a lower odor selectivity for AON-projecting output neurons (e.g. 50% of MTCs responded to only 3 or 4 odors for the APC- and AON-traced MTC, respectively. (Absolute numbers of responding neurons for different number of odors (AON and APC respectively), from 1 to 8 odors: 150, 152; 124, 111; 104, 77; 79, 53; 62, 40; 48, 32; 34, 18; 24,7)).

To analyze odorant response strength across cells, we ranked each unit’s odorant responses by amplitude, from the largest (rank 1) to the smallest (rank 8). Odorant responses were then ordered by rank and averaged in a rank-specific manner. AUC of rank-averaged odor response amplitude for APC- and AON-traced MTC was not statistically different for the two populations (Fig. 3D, AON-traced AUC 881.12 ± 615.93 (mean ± SD); APC-traced AUC 841.96 ± 736.58; *p* = 0.06 Mann–Whitney U test), further demonstrating no major impact of signal amplitudes.

Comparing response amplitudes of cells that responded selectively to only one odorant (AON 33 cells, APC 43 cells) to those that responded to all odorants (AON 25 cells, APC 7 cells) also revealed no significant difference, neither for AON- nor APC-traced cells, arguing that the difference in the number of effective odorants was not the result of an increase in absolute response amplitudes (Fig. 3E, AON-traced, one odorant 205.35 ± 141.01 (mean ± SD), all odorants 316.40 ± 216.86; APC-traced, one odorant 257.08 ± 194.69, all odorants 344.94 ± 153.99 ANOVA *p* = 0.09). We calculated both lifetime sparseness (Fig. 3F, left) as a measure of single cell selectivity as well as population sparseness (Fig. 3F, right) as an indicator of how sparse the populations were reacting to individual stimuli. Both lifetime and population sparseness showed a statistical difference between projection-defined populations (lifetime sparseness: *p* = 0.002, Mann–Whitney U test, and population sparseness: *p* = 0.015, Mann–Whitney U test) with APC-traced MTCs showing a higher selectivity than AON-traced MTCs. In conclusion, MTCs projecting to AON are more broadly tuned compared to MTCs projecting to APC.

### MTC concentration responses: APC-traced cells show increased sensitivity

**Fig. 4:**
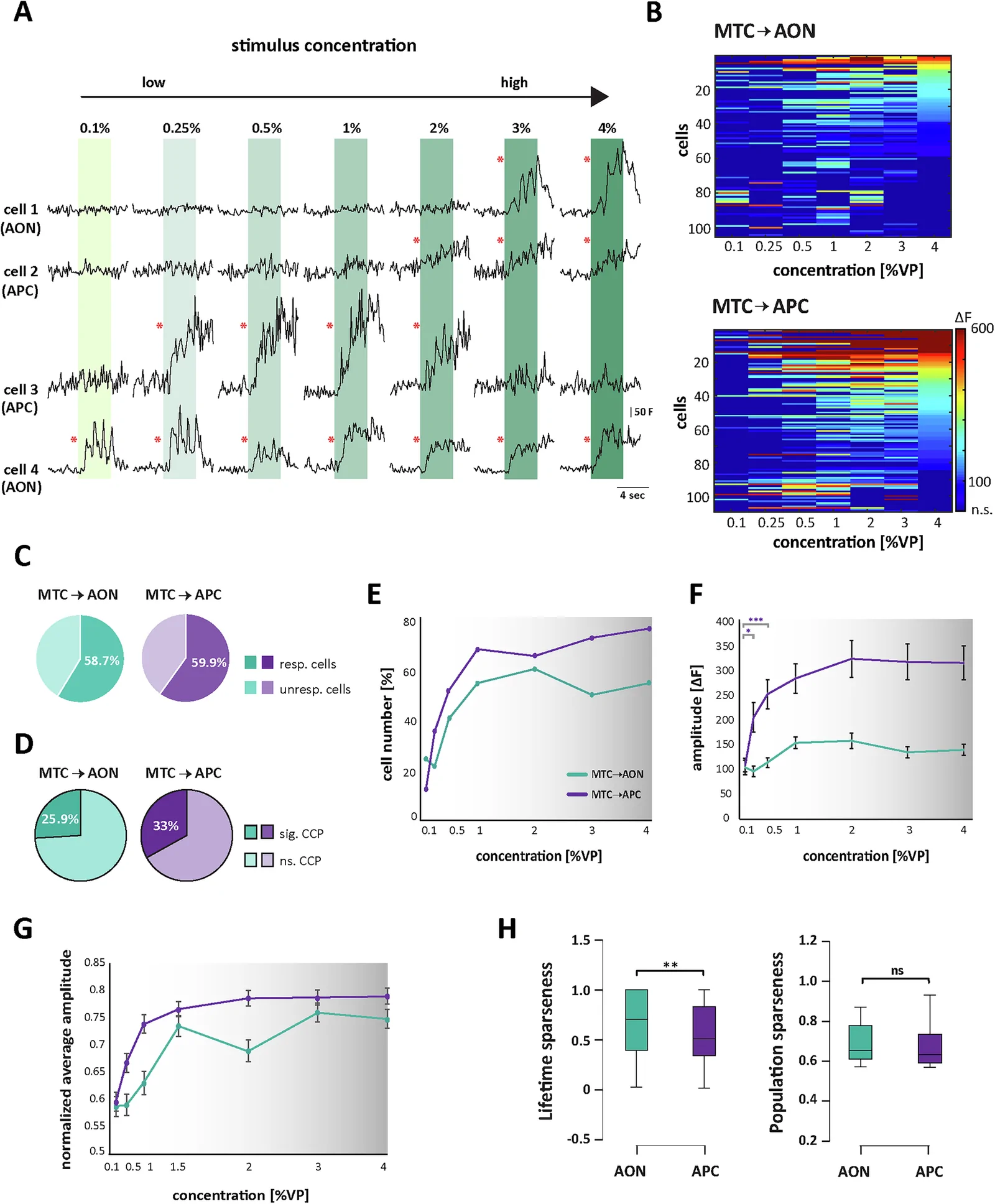
APC-traced MTCs exhibit enhanced concentration sensitivity over AON-traced MTCs. **A** Exemplary Ca^2+^-traces at different odor concentrations (MV, IAA, MV, EB from top to bottom) for AON- and APC-traced cells. **B** Population response plot of AON- (top, 105 cells) and APC- (bottom, 109 cells) traced MTCs. Only cells that responded significantly to at least one concentration were included. Cells were sorted according to their response strength to the highest concentration. **C** Proportion of cells responding to at least one odor for AON- (left) and APC- (right) traced MTCs. **D** Proportion of significant cell-concentration pairs for AON- (left) and APC- (right) traced MTCs. **E** Percentage of MTCs significantly responding to the different applied concentrations. **F** Averaged maximum amplitude (Δ*F*) of AON- and APC-traced MTCs at the applied odor concentrations (mean ± SEM, *n* = 5 each). Kruskal–Wallis test *p* < 0.001; Dunn’s Post-Hoc comparisons for APC *p* = 0.011 and *p* = < 0.001, for AON *p* = 1 and *p* = 0.096, respectively. **G** Normalized average amplitudes of AON- and APC-traced MTCs at the applied odor concentrations (*n* = 5 each). Data are presented as mean values ± SEM. Mann–Whitney U test, *p* = 0.001. **H** Lifetime and population sparseness analysis for AON- and APC-traced MTCs (*n* = 5 each). Boxplots display the median with horizontal lines, and the edges indicate the 25th and 75th percentiles, with maximum and minimum values plotted, excluding outliers. Statistical analysis was performed with two-sided Mann–Whitney U test: for lifetime sparseness: *p* = 0.002, for population sparseness: *p* = 0.805.

Next, we tested for differences in odor sensitivity among AON- or APC-projecting MTCs by applying odors at different concentrations (0.1 to 4% saturated vapor pressure (VP), Fig. 4A). A similar fraction of AON- and APC-traced MTCs responded significantly to at least one odor concentration across all tested odorants (58.7%, 105 out of 179 neurons, AON-traced; 59.9%, 109 out of 182 cells APC-traced; χ2 (3) = 0.09, *p* = 0.99, Fig. 4C). When calculated on a cell-concentration basis, AON-traced MTCs exhibited a significantly lower fraction of significant responses compared to APC-traced cells (cell-concentration pairs (CCPs) AON-traced: 25.9% significant CCPs 325 out of 1253, APC-traced: 33.04% significant CCPs, 421 out of 1274; χ2 (3) = 15.61, *p* = 0.001 (Fig. 4D)). The moderate number of CCPs depicting significant responses for both populations argues that the tested concentration range is well within physiological limits. For both populations, the number of responsive units increased with increasing odor concentration (26/14 cells for the lowest concentration vs 58/84 cells for the highest concentration for AON/APC-traced cells, respectively, Fig. 4E). Interestingly, however, the percentage of responsive AON-traced cells was lower at every tested concentration (Fig. 4B, E). Whereas the averaged odor response amplitude for both APC- and AON-traced MTCs significantly increased with odor concentration (responses at 0.1% vs. 4% saturated vapor pressure; AON-traced 106.11 ± 154.39 vs. 141.68 ± 114.7 Δ*F* (mean ± SD) *p* < 0.001 Wilcoxon signed-rank test; APC-traced 110.6 ± 142.55 vs. 318.98 ± 360.77 Δ*F* *p* < 0.001 Wilcoxon signed-rank test, Fig. 4F), APC-traced cells reacted far more strongly to an increase in odor concentrations (189.09% and 33.01% mean normalized amplitude increase comparing the highest to the lowest concentration for APC- and AON-traced cells, respectively). Calculating the area under the concentration-averaged odor response amplitude curves showed apparent differences between APC- and AON-traced populations, with APC-traced neurons displaying significantly higher response amplitudes (AON-traced AUC 347.35 ± 354.76 (mean ± SD); APC-traced AUC 504.18 ± 1049.26; *p* < 0.001 Mann–Whitney U test). Differences between the two traced populations were particularly clear at lower concentrations as only APC-traced cells displayed significant differences in amplitudes comparing 0.1 vs. 0.25% and 0.1 vs. 0.5% (Kruskal–Wallis test *p* < 0.001; Dunn’s Post-Hoc comparisons for APC *p* = 0.011 and *p* < 0.001, for AON *p* = 1 and *p* = 0.096, respectively; Dunn’s test revealed significant differences for both populations at following concentrations: for 0.1 vs. 1, AON *p* < 0.001, APC *p* < 0.001; for 0.1 vs. 2, AON *p* < 0.001, APC *p* < 0.001; for 0.1 vs. 3, AON *p* = 0.001, APC *p* < 0.001; for 0.1 vs. 4, AON *p* < 0.001, APC *p* < 0.001; for 0.25 vs. 1 AON *p* < 0.001, APC *p* = 0.002, for 0.25 vs. 2, AON *p* < 0.001, APC *p* < 0.001; for 0.25 vs. 3 AON *p* = 0.002, APC *p* < 0.001; for 0.25 vs. 4, AON *p* < 0.001, APC *p* < 0.001, Fig. 4F, for simplicity, significant differences observed in both populations are omitted in the figure). Differences in average amplitude were even evident when responses were normalized to the maximum response for each respective unit (Fig. 4G, *p* = 0.001, Mann–Whitney U test). Lifetime (Fig. 4H, left) and population sparseness (Fig. 4H, right) were calculated for projection target-defined populations. While population sparseness did not show a statistical difference between populations, lifetime sparseness was larger for AON projecting cells (lifetime sparseness: *p* = 0.002, Mann–Whitney U test, and population sparseness: *p* = 0.805, Mann–Whitney U test).

Taken together, MTCs projecting to the AON showed less variance in odor-concentration responses than APC-traced cells but sparser concentration coding, suggesting that both populations transmit concentration-dependent signals using different strategies.

### Target-dependent dual tracing reveals spatially segregated OB output neuron populations

So far, we have focused our analysis on comparing populations containing MTCs projecting to different olfactory cortical areas. However, MCs and TCs have been reported to differ in response profiles^31,33,41,79^. Since AON and APC, the two most anterior areas of the olfactory cortex, receive input from both OB output cell types^33^, we asked if a different proportion of MCs and TCs in the two traced populations might account for the observed differences.

**Fig. 5:**
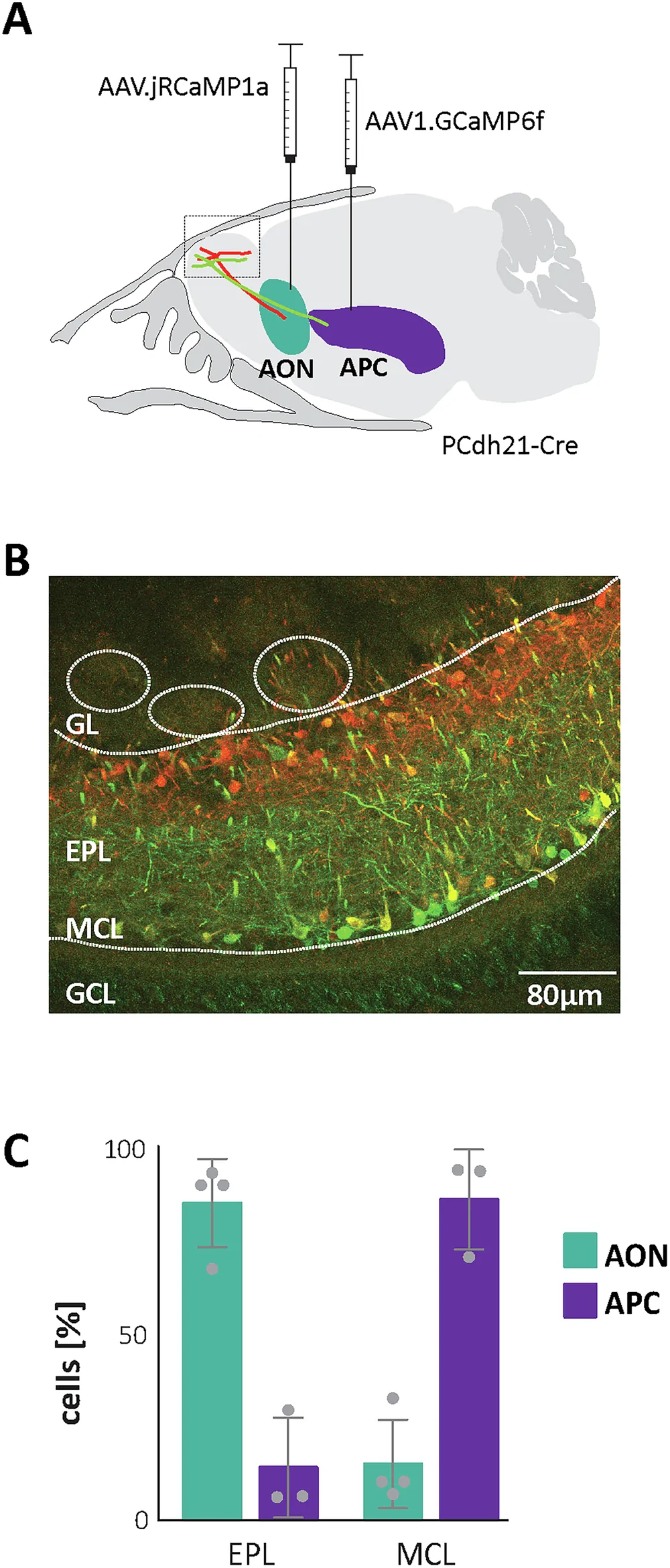
Target-dependent dual tracing reveals segregated populations of OB output neurons in the EPL and MCL. **A** Dual-tracing approach with different genetically encoded calcium indicators (GCaMP6f & jRCaMP1a) injected into APC and AON. **B** Histological section of the OB of mice injected with a green GECI into APC and a red GECI into AON. **C** Percentage of traced cells residing in the EPL or MCL for AON- and APC-traced OB neurons (*n* = 4 mice for AON, *n* = 3 mice for APC). Data are presented as mean values ± SD.

First, we quantified the total numbers of MCs and TCs in histological sections by simultaneously tracing from the AON and APC using dual-color tracing with red and green fluorescent marker proteins (Fig. 5A, B). We found that innervation-area-dependent labeled cell populations could be well separated. However, overlap of green and red labeling was also observed, which differed between cell populations: while half of the MCs population (49.2 ±7.4 % (mean ± SD) showed an overlap, for TCs, overlap was only 28.9 ±5.9 % (mean ± SD). Thus, the separation is, in agreement with previous reports^32,33^, not absolute. Marker protein-expressing cells were separated into MCs and TCs according to their location in the MCL or EPL. Most AON-traced cells (80.6%, 79 out of 98 cells (*n* = 4 mice) χ2 (1) = 36.73, *p* < 0.001) were found in the EPL. In contrast, APC-traced cells (82%, 55 out of 67 cells; χ2 (1) = 27.59, *p* < 0.001) were primarily located in the MCL (Fig. 5C), demonstrating an innervation area-dependent segregation. In preparations that were simultaneously traced, we separated double-labeled cells from single-traced cells and analyzed their respective GCaMP6f-signals upon odorant stimulation from 2P imaging. From those cell populations (AON-traced, APC-traced, and yellow (double-labeled cells), we plotted the cumulative probability of responding cells and calculated lifetime and population sparseness (Supplementary Fig. 2) to compare them to our previous findings.

Cumulative probability and sparseness plots showed that double-labeled cells had properties unlike those of AON- or APC-projecting cells but fell right between those of singly projecting cells. Interestingly, significance levels for difference between AON and APC projecting MTCs became larger through removal of the dually projecting cells for both lifetime (Kruskal–Wallis test *p* < 0.001, Dunn’s Post-Hoc comparisons: AON-projecting vs. APC-projecting, *p* < 0.001, AON-projecting vs. double-projecting TCs *p* = 0.134, APC-projecting vs. double-projecting TCs *p* = 0.005) and population sparseness (Kruskal–Wallis test *p* = 0.005, Dunn’s Post-Hoc comparisons AON-projecting vs. APC-projecting, *p* = 0.001, AON-projecting vs. double-projecting TCs *p* = 0.191, APC-projecting vs. double-projecting TCs *p* = 0.052), suggesting that, although not all OB output neurons project to a single cortical area, those that do show a highly significant difference in their coding properties.

### AON-targeting TCs display high odor responsiveness but low odor selectivity

**Fig. 6:**
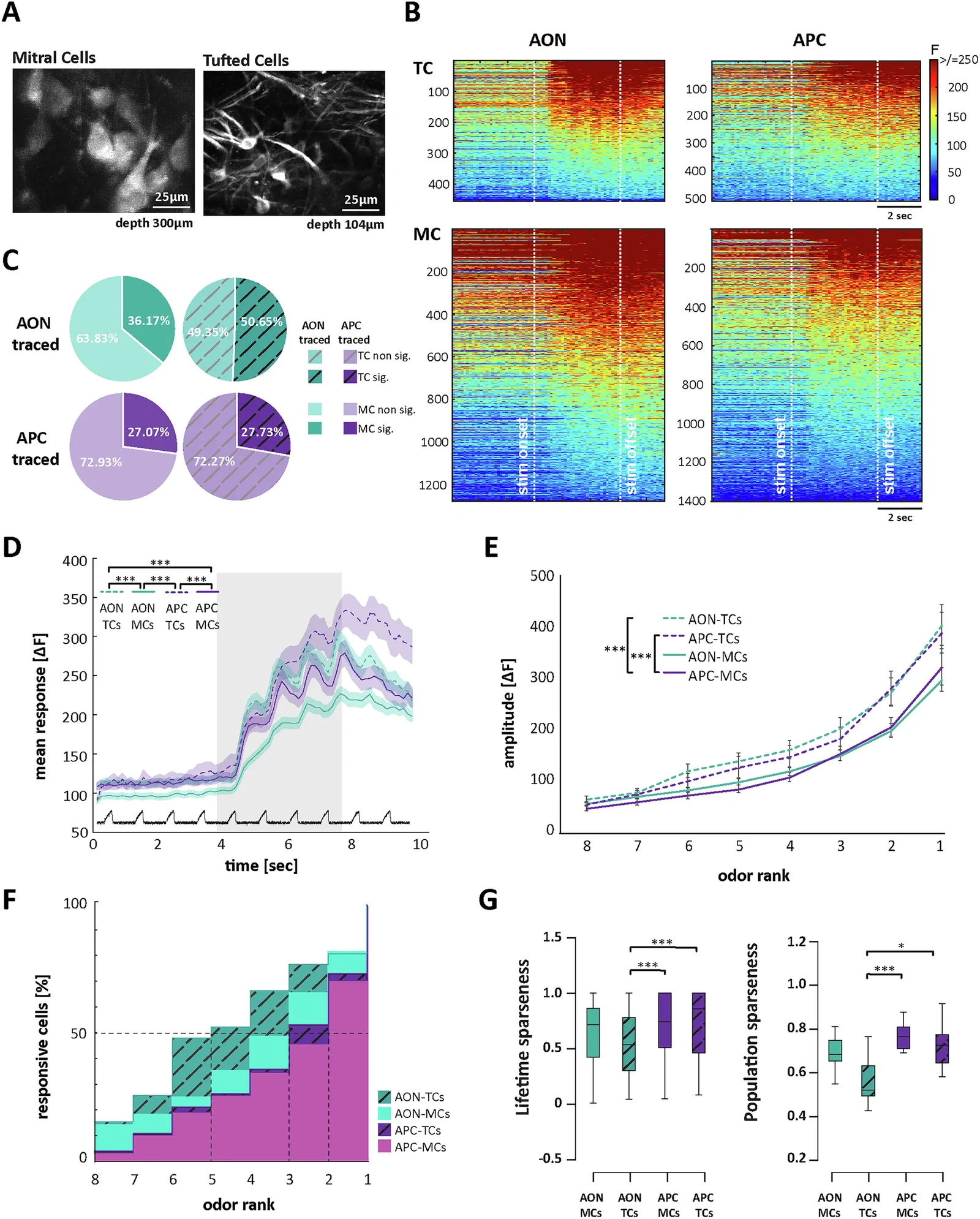
AON-targeting TCs exhibit high responsiveness and low selectivity. **A** Exemplary FOV of TCs in the EPL and MCs in the MCL. **B** Population response plots for the four different OB output streams (AON-TCs: 456 COPs, APC-TCs: 512 COPs, AON-MCs: 1280 COPs, APC-MCs: 1400 COPs). White dashed lines indicate the onset of odor stimulation. Inhalation-linked calcium fluctuations are most strongly evident for AON-TCs. **C** Proportion of significant COPs traced from AON (top) and APC (bottom) categorized into TCs (right) and MCs (left). **D** Averaged odor response of significant odor-responsive cells categorized by target area and cellular identity (*n* = 5 for AON-traced, *n* = 5 for APC-traced, AON-TCs: 231, APC-TCs: 142, AON-MCs: 463, APC-MCs: 379 cells). Lines represent mean values with bands as ± SEM. Statistical analysis was performed with *p* < 0.01 two-sided Kruskal–Wallis test, please see Results section ‘AON-targeting TCs display high odor responsiveness but low odor selectivity’ for pairwise comparisons. **E** Averaged maximum odor response amplitudes (Δ*F*) of AON- and APC-traced MCs and TCs ordered from the weakest (8, left) to the strongest (1, right) (mean ± SEM, *n* = 5 for AON-traced, *n* = 5 for APC-traced) (*p* < 0.001 two-sided Kruskal–Wallis test; Dunn’s Post-Hoc comparisons: *p* < 0.001 AON-TCs vs. APC-MCs, *p* = 0.001 APC-MCs vs. APC-TCs). **F** Cumulative probability plot indicating the fraction of cells of the whole population reacting to up to 8 odors. The probability of reacting to a high number of odors is markedly higher in AON-TCs. **G** Lifetime and population sparseness analyses of four different streams for selectivity profiles (*n* = 5 for AON-traced, *n* = 5 for APC-traced). Boxplots display the median with horizontal lines, and the edges indicate the 25th and 75th percentiles, with maximum and minimum values plotted, excluding outliers. Statistical analysis was performed with two-sided Kruskal–Wallis test, *p* = 0.04 (lifetime sparseness) and *p* = 0.013 (population sparsenss), Dunn’s Post-Hoc comparisons for lifetime sparseness: *p* = 0.002 for AON-TCs vs. APC-MCs, *p* = 0.003 for AON-TCs vs. APC-TCs; for population sparseness: *p* = 0.002 for AON-TCs vs. APC-MCs, *p* = 0.020 AON-TCs vs. APC-TCs.

Next, we aimed to test for distinctive projection area-dependent properties of MCs vs. TCs. Here, we used our functional 2-photon microscopy dataset, obtained with the initial approach (Fig. 1A), separated into MCs and TCs, though it contained potentially dually projecting cells, as this dataset was much larger and thus more representative. As in previous reports^50–52,80,81^, ROIs were classified as MCs or TCs based on imaging depth, soma size, and cell morphology visualized in high-resolution z-stacks. Figure 6A depicts two example fields of view categorized as TCs and MCs. Classification was performed blinded to animal identities and response amplitudes. Separating the differentially traced populations, we found that both contained an equal ratio of tufted to mitral cells, i.e., in both populations, only about a quarter of the cells were tufted cells. Those tufted cells were more responsive to the tested odor panel as more TCs showed at least one significant odor response than MCs for both traced populations (85.96% vs 74.375%, respectively AON-traced TCs 49 out of 57 cells, AON-traced MCs 119 out of 160 cells; χ2 (3) = 3.81, *p* = 0.282; APC-TCs 45 out of 64 cells, APC-MCs 116 out of 175 cells; χ2 (3) = 0.36, *p* = 0.947). When calculated on a cell-odor basis, AON-TCs showed the highest odor reactivity (AON-TCs, 50.65% significant COPs, 231 out of 456; AON-MCs, 36.17% significant COPs, 463 out of 1280; APC-TCs, 27.73% significant COPs, 142 out of 512; APC-MCs, 27.07% significant COPs, 379 out of 1400; χ2 (3) = 10.39, *p* = 0.015, Fig. 6C). Of all AON/APC-traced cells, 13.3/7.4% TC-COPs displayed a significant response, and 26.7/19.8% MC-COPs, respectively.

Odor response kinetics were variable for each cell population (Fig. 6B). Averaging odor responses for all significant COPs, however, displayed apparent differences between the populations (Fig. 6D). Analyzing the area under the odor response curve for the different cell populations (AON-projecting MCs vs. TCs and APC-projecting MCs vs. TCs) revealed that, as expected from previous literature^6,31,33^, mean calcium responses differed significantly between MCs and TCs (both within and between projection targets). We did not detect significant differences between population-averaged calcium traces of TCs or MCs, respectively, with different projection targets (*p* < 0.01 Kruskal–Wallis test; Dunn’s Post-Hoc comparison, *p* < 0.001 AON-MCs vs. AON-TCs, *p* = 0.103 AON-MCs vs. APC-MCs, *p* < 0.001 AON-MCs vs. APC-TCs, *p* < 0.001 AON-TCs vs. APC-MCs, *p* = 0.375 AON-TCs vs. APC-TCs, *p* < 0.001 APC-MCs vs. APC-TCs). We analyzed all four groups for their odor selectivity to further test for differences in the traced neuronal populations.

AON-TCs were exceptionally broadly-tuned. 53% of all AON-TCs (26 out of 49 cells) responded to 5 or more odorants compared to only 26.6% for APC-TCs (12 out of 45 cells) (AON-MCs 35.3%, 42 out of 119 cells; APC-MCs 26.7%, 31 out of 116 cells) (Fig. 6F). The rank-averaged odor response amplitude for the four groups did differ significantly for AON-TCs vs. APC-MCs and APC-MCs vs. APC-TCs but were not different for TCs or MCs of different projection targets (Fig. 6E) (*p* < 0.001 Kruskal–Wallis test; Dunn’s Post-Hoc comparison: *p* = 0.073 AON-MCs vs. AON-TCs, *p* = 0.092 AON-MCs vs. APC-MCs, *p* = 0.092 AON-MCs vs. APC-TCs, *p* < 0.001 AON-TCs vs. APC-MCs, *p* = 0.912 AON-TCs vs. APC-TCs, *p* = 0.001 APC-MCs vs. APC-TCs). Independent of innervation area, TCs displayed higher mean amplitudes, particularly evident in the strongest odor responses (Fig. 6E). Thus, differences in maximum response amplitudes are unlikely to account for the innervation area-dependent impact on odor responsiveness.

To quantify differences in selectivity between the cell types, we calculated lifetime sparseness (Fig. 6G, left). AON projecting TCs exhibited lower sparseness than APC projecting cell types (*p* = 0.04 Kruskal–Wallis test; Dunn’s Post-Hoc comparison, *p* = 0.102 AON-MCs vs. AON-TCs, *p* = 0.064 AON-MCs vs. APC-MCs, *p* = 0.049 AON-MCs vs. APC-TCs, *p* = 0.002 AON-TCs vs. APC-MCs, *p* = 0.003 AON-TCs vs. APC-TCs, *p* = 0.556 APC-MCs vs. APC-TCs). This was also reflected in population sparseness (Fig. 6G right), though the lower sparseness of AON MCs did not reach significance levels (*p* = 0.013 Kruskal–Wallis test; Dunn’s Post-Hoc comparison, *p* = 0.098 AON-MCs vs. AON-TCs, *p* = 0.129 AON-MCs vs. APC-MCs, *p* = 0.505 AON-MCs vs. APC-TCs, *p* = 0.002 AON-TCs vs. APC-MCs, *p* = 0.020 AON-TCs vs. APC-TCs, *p* = 0.394 APC-MCs vs. APC-TCs). This means that the lower selectivity we observed for the AON-traced cell population primarily stems from the low selectivity of AON-TCs.

To assess whether this difference in selectivity increases when we filter out potentially dual-projecting cells, we used the very limited dataset we had for successful dual labeling (Supplementary Fig. 2) and analyzed the properties of singly and dually projecting MCs and TCs based on their projection targets. As expected, in both cell types, dually projecting streams displayed properties that fell between AON- and APC-projecting streams. Lifetime and population sparseness analyses showed distributions and levels similar to those from analyses of differentially projecting mixed MTC populations (Supplementary Fig. 3). MCs and TCs showed significant differences in lifetime and population sparseness between AON- and APC-projecting populations, consistent with the target-dependent properties of MTCs. (TC lifetime sparseness: Kruskal–Wallis test *p* = 0.016, Dunn’s posthoc comparisons: AON-TCs vs. APC-TCs *p* = 0.009, AON-TCs vs. double-projecting TCs *p* = 0.165, APC-TCs vs. double-projecting TCs *p* = 0.066; TC population sparseness: Kruskal–Wallis test *p* < 0.001, Dunn’s Post-Hoc comparisons: AON-TCs vs. APC-TCs p < 0.001, AON-TCs vs. double-projecting TCs *p* = 0.011, APC-TCs vs. double-projecting TCs *p* = 0.112; MC lifetime sparseness: Kruskal–Wallis test *p* = 0.016, Dunn’s Post-Hoc comparisons: AON-MCs vs. APC-MCs, *p* = 0.005, AON-MCs vs. double-projecting MCs *p* = 0.324, APC-MCs vs. double-projecting MCs *p* = 0.051; MC population sparseness: Kruskal–Wallis test *p* = 0.077, Dunn’s Post-Hoc comparisons: AON-MCs vs. APC-MCs *p* = 0.024, AON-MCs vs. double-projecting MCs *p* = 0.305, APC-MCs vs. double-projecting MCs *p* = 0.216)

### APC-targeting TCs exhibit enhanced concentration sensitivity

**Fig. 7:**
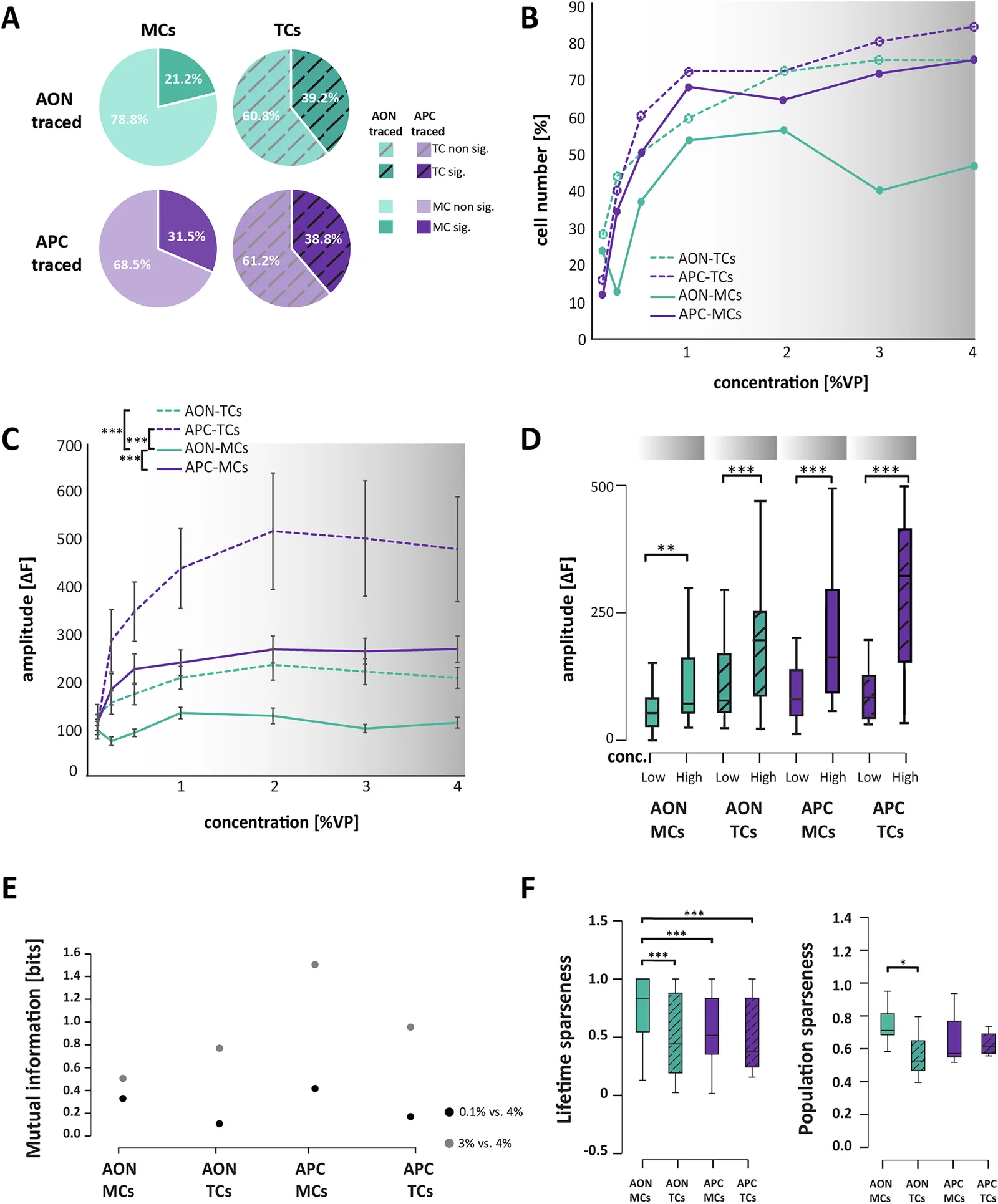
APC-targeting TCs exhibit enhanced concentration sensitivity. **A** Proportion of significant CCPs traced from AON (top) and APC (bottom) categorized into TCs (right) and MCs (left). **B** Percentage of AON- and APC-traced cells separated by MC and TC streams responding to different odor concentrations. **C** Averaged maximum amplitudes of AON- (*n* = 5) and APC-traced (*n* = 5) MCs and TCs. APC-traced TCs show a much higher increase in amplitude in the lower concentrations than the other cell populations (mean ± SEM). Two-sided Kruskal–Wallis test was used for statistical analysis, *p* < 0.001, Dunn’s Post-Hoc comparisons: *p* < 0.001 AON-MCs vs. AON-TCs, *p* < 0.001 AON-MCs vs. APC-MCs, *p* < 0.001 AON-MCs vs. APC-TCs. **D** Concentration averaged odor responses of 4 OB output streams to the lowest (0.1% VP) and highest (4% VP) odor concentrations (*n* = 5 for AON-traced, *n* = 5 for APC-traced, AON-MCs: 73, AON-TCs: 32, APC-MCs: 84, APC-TCs: 25 units). Boxplots indicate the median with horizontal lines, and the edges show the 25th and 75th percentiles, with maximum and minimum values plotted, excluding outliers. Statistical analysis was done with two-sided Wilcoxon signed-rank test, AON-MCs *p* = 0.005, AON-TCs *p* = 0.003, APC-MCs *p* < 0.001, APC-TCs *p* < 0.001. **E** Mutual information analysis to decode the sensitivity profiles of four different streams. Black dots representing the comparison of the lowest (0.1%) to the highest (4%) concentration, while grey dots represent the comparison of responses to the highest (4%) to the second highest (3%) concentration. **F** Lifetime and population sparseness analyses of four different streams for selectivity profiles (*n* = 5 for AON-traced, *n* = 5 for APC-traced). Boxplots display the median with horizontal lines, and the edges indicate the 25th and 75th percentiles, with maximum and minimum values plotted, excluding outliers. Statistical analysis was performed with a two-sided Kruskal–Wallis test, *p* < 0.001 (lifetime sparseness) and *p* = 0.097 (population sparsenss), Dunn’s Post-Hoc comparisons for lifetime sparseness: *p* < 0.001 AON-MCs vs. AON-TCs, *p* < 0.001 AON-MCs vs. APC-MCs, *p* < 0.001 AON-MCs vs. APC-TCs; for population sparseness: *p* = 0.012 AON-MCs vs. AON-TCs.

Next, we tested for the concentration dependence of the different cell populations. A similar fraction of AON- and APC-traced MTCs responded significantly to at least one odor concentration (55.3%, 73 out of 132 neurons, AON-traced MCs; 68.1%, 32 out of 47 neurons, AON-traced TCs; 58.7%, 84 out of 143 cells APC-traced MCs; 64.1%, 25 out of 39 cells APC-traced TCs, χ2 (3) = 1.67, *p* = 0.64). When calculated on a cell-concentration basis, AON-traced MCs exhibited a lower fraction of significant responses compared to the other populations (cell-concentration pairs (CCP): AON-traced TCs, 39.2% significant CCPs, 129 out of 329; AON-traced MCs, 21.2% significant CCPs, 196 out of 924; APC-traced TCs, 38.8% significant CCPs, 106 out of 273; APC-traced MCs, 31.5% significant CCPs, 315 out of 1001, χ2 (3) = 6.73, *p* = 0.081, Fig. 7A). For all populations, the number of responsive units increased with increasing odor concentration (9/24 AON-traced TCs for the lowest/highest concentration, 17/34 AON-traced MCs; 4/21 APC-traced TCs; 10/63 APC-traced MCs), though the percentage of responsive AON-traced MCs was lower at every tested concentration (Fig. 7B). The concentration-averaged odor response amplitude for all groups increased significantly with odor concentration (Fig. 7D, responses at 0.1% vs. 4% saturated vapor pressure; AON-MCs 96.18 ± 164.11 vs. 112.47 ± 97.17 *ΔF* (mean ± SD) *p* = 0.005 Wilcoxon signed-rank test; AON-TCs 128.77 ± 129.05 vs. 208.3 ± 124.96 *ΔF*, *p* = 0.003 Wilcoxon signed-rank test; APC-MCs 112.83 ± 156.4 vs. 269.87 ± 259.24 Δ*F*, *p* < 0.001 Wilcoxon signed-rank test; APC-TCs 103.1 ± 82.27 vs. 484 ± 562.56 Δ*F,* *p* < 0.001 Wilcoxon signed-rank test). Analyzing the AUC revealed that all groups showed a significant difference to the amplitude increase in AON-traced MCs (*p* < 0.001 Kruskal–Wallis test; Dunn’s Post-Hoc comparison, *p* < 0.001 AON-MCs vs. AON-TCs, *p* < 0.001 AON-MCs vs. APC-MCs, *p* < 0.001 AON-MCs vs. APC-TCs, *p* = 0.9 AON-TCs vs. APC-MCs, *p* = 0.13 AON-TCs vs. APC-TCs, *p* = 0.12 APC-MCs vs. APC-TCs) (Fig. 7C). APC-traced TCs reacted the strongest to an increase in odor concentrations (445% amplitude increase comparing the highest to the lowest mean concentration for APC-traced TCs; 140% for APC-traced MCs; 62% for AON-traced TCs and 16% for AON-traced MCs).

We estimated the capacity of separated cell populations for concentration coding by calculating the mutual information between odor responses at different concentrations, as mutual information should be higher when concentrations are similar. Comparison of mutual information between similar (3% vs 4% VP) and different concentrations (0.1% vs 4% VP) showed that APC-traced cells consistently exhibited a greater difference in mutual information (Fig. 7E). Mutual information values for AON MC were especially low and did not increase substantially even at similar concentrations. We furthermore calculated lifetime and population sparseness for the concentration-dependent responses of AON and APC projecting TCs and MCs (Fig. 7F). Lifetime sparseness was significantly higher in AON-MCs compared to all other cell populations (*p* < 0.001 Kruskal–Wallis test; Dunn’s Post-Hoc comparison: *p* < 0.001 AON-MCs vs. AON-TCs, *p* < 0.001 AON-MCs vs. APC-MCs, *p* < 0.001 AON-MCs vs. APC-TCs, *p* = 0.534 AON-TCs vs. APC-MCs, *p* = 0.819 AON-TCs vs. APC-TCs, *p* = 0.404 APC-MCs vs. APC-TCs). Population sparseness showed sparser coding in AON-projecting MCs, which was significantly different from TCs that projected to the same region (*p* = 0.097 Kruskal–Wallis test; Dunn’s Post-Hoc comparison: *p* = 0.012 AON-MCs vs. AON-TCs, *p* = 0.153 AON-MCs vs. APC-MCs, *p* = 0.229 AON-MCs vs. APC-TCs, *p* = 0.284 AON-TCs vs. APC-MCs, *p* = 0.194 AON-TCs vs. APC-TCs, *p* = 0.820 APC-MCs vs. APC-TCs).

In conclusion, APC projecting cells exhibit the strongest capacity for concentration-dependent responses. This suggests that APC-projecting cells convey concentration-dependent information by increasing response strength, whereas AON-projecting MCs respond more sparsely to different odor concentrations, which might point to population-based concentration coding.

### Projection target defines inhalation-linked dynamics in TCs

**Fig. 8:**
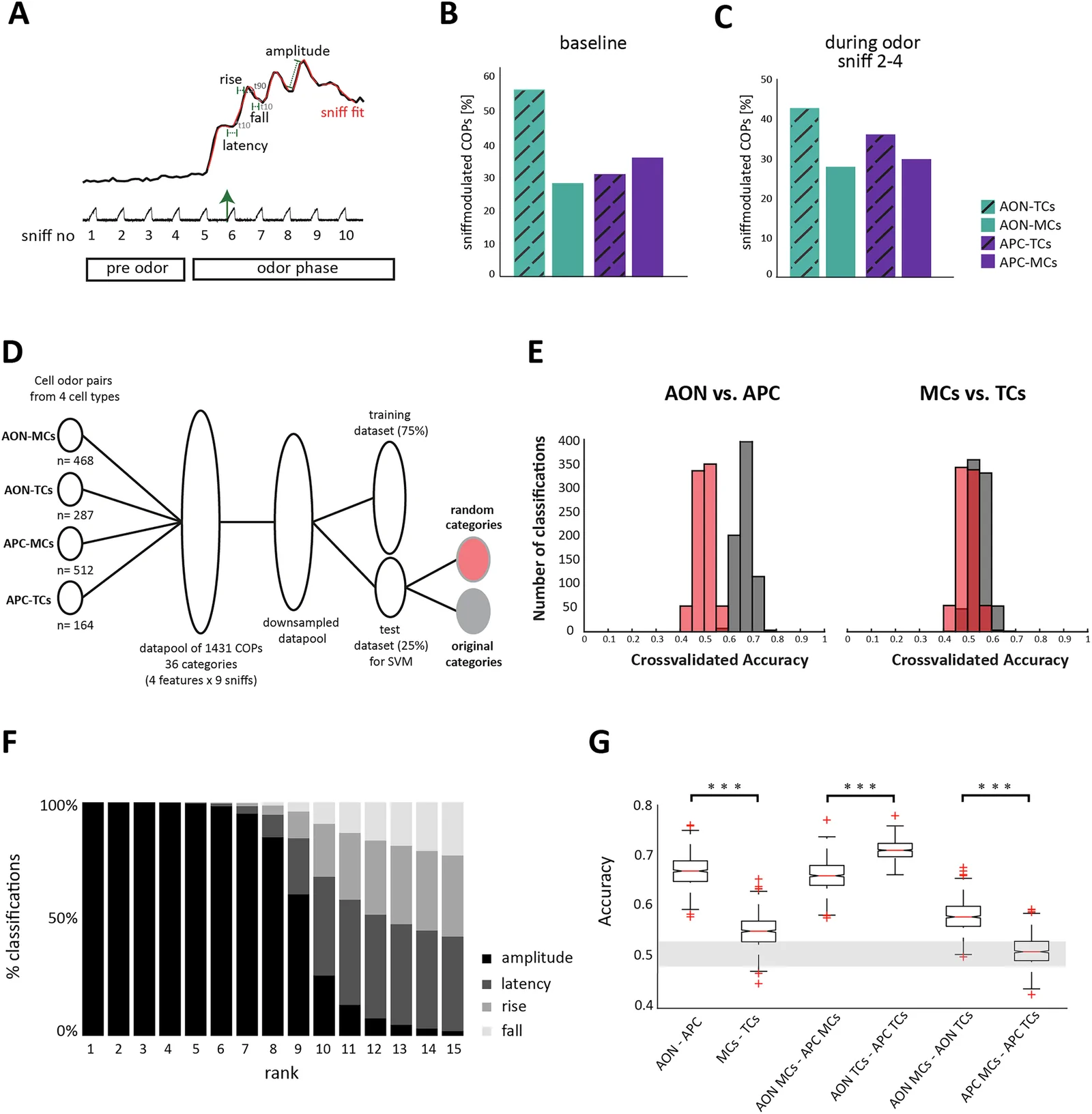
Target area dominates cell identity in shaping neuronal responses. **A** Schematic of the parameters extracted by the custom algorithm fitting the optical signals to a double sigmoid function (see “Materials and Methods”). **B** Percentage of cell-odor pairs showing inhalation-linked calcium transients before onset of the odor presentation. **C** Percentage of cell-odor pairs showing inhalation-linked calcium transients during odor presentation. **D** Schematic of data handling for support vector machine (SVM) decoder. **E** Cross-validated classification performance for distinguishing target-defined MTCs (left) compared to MCs and TCs (right). **F** Percentage of classifications plotted against feature rank for SVM classification. **G** Accuracy of the decoder for separating different combinations of the four OB output streams (*n* = 5 for AON-traced, *n* = 5 for APC-traced) based on cellular identity and target region, central mark = median values, box edges = 25th and 75th percentiles, whiskers extend to the most extreme data points excluding outliers; ‘+‘ outliers. Two-sided Mann–Whitney U test was used for statistical analysis, *p* < 0.001.

Phasic activity related to the respiratory cycle can be observed in OB output neurons^82–84^ from where it is propagated to OC, including PC^85–89^ and AON^90^. TCs typically react more strongly to afferent input^79^ and might reflect rhythmic respiratory events better than MCs^47^. Plotting the mean population response of AON- and APC-traced MCs and TCs revealed a strong, regular phasic calcium signal during the odor response (Fig. 6D). To further analyze sniff modulations, we fitted the calcium traces to a double sigmoid function. Even before odorant presentation, many recorded cells showed detectable “spontaneous” sniff amplitudes. Over 55% of odor-responsive AON-TCs displayed inhalation-linked phasic modulations before odor onset. In comparison, only ~30% of the other cell populations showed similar responses (Fig. 8B, AON-traced TCs 55.9%, AON-traced MCs 27.8%, APC-traced TCs 30.5%, APC-traced MCs 35.5%). Analyzing phasic “sniff-modulated” odor responses further revealed marked differences between the populations. Cell-odor pairs (COPs) were classified as significantly “sniff-modulated” if two criteria were met: (1) fluorescence trace fluctuations were considered as odor-modulated if the maximum amplitude during odor presentation was at least 1.5 times higher compared to the pre-odor period. (2) fluorescence trace fluctuations were considered as sniff modulated (as opposed to a tonic response) if the relative sniff amplitude, defined as the signal amplitude following the sniff divided by the amplitude before the sniff, was less than 1.1 (see “Materials and Methods”). AON-traced TCs displayed the highest amount of significant inhalation-modulated COPs during odor application (Fig. 8C, AON-traced TCs 42.5%; AON-traced MCs 27.7%; APC-traced TCs 35.9%; APC-traced MCs 29.6%). In general, consistent with previous reports, TCs showed stronger sniff modulation than MCs in the respective target area. However, the degree of TC-sniff modulation was strongly influenced by their innervation area.

We used features (amplitude, rise time, latency, and fall) extracted separately for each sniff from fitted 2-photon calcium imaging traces (see methods, Fig. 8A) comprising 36 sniff-features (4 features x 9 sniffs). These features were used as input parameters for a support vector machine (SVM) classifier analysis. Our data pool contained 1431 cell odor pairs (AON mitral 468, AON tufted 287, APC mitral 512, APC tufted 164). Data was downsampled to match the number of data points in the smallest category (Fig. 8D). To calculate cross-validated accuracy values, the downsampled data was split into training (75%) and testing (25%) sets, while maintaining a 1:1 category distribution. We report mean test accuracies of the test dataset for both original and random categories. The cross-validated accuracy for MTCs innervating the AON versus those innervating APC was 0.65, which is clearly separated from the accuracy values centered around the chance level (0.5) obtained from random categories (Fig. 8E, left). Classifier performance was substantially reduced and nearly indistinguishable from the accuracies obtained from random categories when classifying MCs vs. TCs (irrespective of innervation area) (Fig. 8E, right).

To measure sniff-feature importance, we employed feature selection algorithms. A filter-type feature selection algorithm (we used a univariate feature ranking for classification using chi-square) measures feature importance based on the characteristics of the features, such as feature variance and feature relevance to the response. This filter-type feature selection is uncorrelated to the training algorithm. Downsampling was repeated 100.000 times (bootstrapping), each followed by feature ranking. The results are depicted in Fig. 8F, demonstrating that the feature “amplitude” had the highest feature importance, followed by “onset” and “risetime”.

We also calculated classification accuracies for other permutations. Classifier performance was statistically different for AON APC vs. mitral tufted cell, mitral vs. tufted cells (pooled across innervation area), and AON vs APC (pooled across cell type) data (Mann–Whitney U test: AON-APC vs. MCs-TCs *p* < 0.001; AON MCs-APC MCs vs. AON TCs-APC TCs *p* < 0.001; AON MCs-AON TCs vs. APC MCs-APC TCs *p* < 0.001). The weakest classification performance was observed for APC-MCs versus APC-TCs, where accuracies dropped to chance level (Fig. 8G).

In conclusion, the SVM classification performance of innervation area-dependent labeled cells outperformed cell-based classification, demonstrating that differences in target area can outweigh pure cell-identity differences.

## Discussion

Parallel processing is one of the most essential features of sensory systems, ensuring fast and reliable perception. In the olfactory system, the two principal OB output neurons, mitral and tufted cells, exhibit different properties^91–93^ and distribute olfactory information to several cortical areas^94^. Even though distinct functions were assigned to olfactory cortical regions^7,38^, both types of output neurons project to overlapping areas within the anterior olfactory cortex, rendering it unclear to what extent different olfactory cortical regions receive segregated information. Alternatively, the encoding of distinct sensory information features could primarily arise from local computations within these cortical areas^95^. Here, we tackled this question by anatomically separating OB outputs based on their projection to the two most anterior olfactory areas, the AON and the APC. Comparing these output streams using in vivo functional calcium imaging revealed marked differences between innervation area-dependent labeled cells, with APC-traced cells showing narrower odor tuning and more concentration-dependent coding than AON-traced cells. A limited set of experiments involving virus injections into AON and APC using different-colored fluorophores allowed us to estimate the number of dually labeled neurons, with approximately one-third of TCs and roughly half of MCs exhibiting double labeling. This labeling likely results from a population of MCs and TCs branching in both the AON and the APC^96^, which is consistent with previous reports^54^. Removing dually traced cells in the analysis of these experiments made distinctions between differentially traced populations clearer, supporting the concept of projection-dependent information routing.

Differences in odor processing between AON and APC projecting populations could result from a difference in cell types within these populations, as it has been shown that most AON-projecting cells are TCs, while most of the APC-traced cells are MCs^32^. We further segregated innervation area-dependent labeled cells into TCs or MCs based on position and morphology, similar to approaches by Kapoor et al.^50^ and others^51,80,97^, which was also genetically confirmed^81^.

Splitting our dataset into MCs and TCs revealed that it contained far fewer TCs than MCs, but both projection-defined populations had similar numbers of each. Therefore, our datasets for AON and APC projecting populations are comparable in cell numbers but do not necessarily reflect physiological compositions.

Differences in odor processing between TCs and MCs have been shown previously, such as stronger afferent evoked excitation demonstrated for TC^79^, or differences in the surrounding inhibitory network, including lateral inhibition^40,47,98^ and are also reflected in our dataset, e.g., in higher response probabilities for TCs than for MCs.

Our data also show differences when considering the same cell type with different projection targets, e.g., AON TCs, which are much less odor-selective than their APC-projecting counterparts (Fig. 6F, G), and APC TCs, which seem to convey more concentration information, at least in terms of an increase in response strength (Fig. 7C). SVM classification accuracy was higher when relying on projection target information than on information about cell type.

Evidence that OB odor information is already processed differently depending on the cortical recipient of this information is a phenomenon that has not been demonstrated so far. Most publications have investigated odor processing within cortical areas^20^ rather than their respective incoming information. Evidence for parallel processing via target-dependent information routing has been shown for projections from higher brain areas, including the visual cortex^99^, amygdala^100^ and the hippocampus^101,102^. To our knowledge, this concept has not been demonstrated for sensory projections prior to cortical processing steps.

The underlying mechanisms for such a projection-dependent separation remain unclear. Differences in response properties might arise from internal factors like channel density (e.g., voltage-gated potassium channels^79^), external factors like stronger afferent evoked excitation demonstrated for TC^79^, or differences in the surrounding inhibitory network, including lateral inhibition^40,47,98^. Cellular disparity has been shown not only in the heterogeneous TC population^91^, but also for MCs. MCs display diverse intrinsic biophysical properties^103–105^, differences in embryonic development^106^ and temporal responses, even when they synapse onto the same glomerulus^107^.

Transcriptional regulation by different neurotrophic factors released by the target areas is another possible explanation. Recent progress in in vitro and in situ sequencing techniques has indeed revealed several subcategories of MCs that differ in projection properties^54^ as well as in the expression of ion channels, cell adhesion, and axon guidance molecules^53^. Therefore, transcriptional differences might account for the variation in odor-coding properties.

Linking a possible function to the discovery of differential odor processing and routing from the OB to olfactory cortical centers is difficult, especially since the functional separation of AON and APC is less than clear. Though very different in anatomical composition, both have been shown to project back to the OB in an activity-dependent manner^108,109^ and to form olfactory memory traces^10,13^. As the APC is considered the “primary” cortical region for odor information processing and odor identification, the function of the AON remains poorly understood, despite notable efforts in recent years.

Our study might help form a new theory based mainly on two of our findings:

(1) We found that projection-target defined TC subpopulations displayed a more heterogeneous response profile than the MC, with APC-TCs exhibiting the strongest increase in response with rising odor concentration (Fig. 7C), and AON-TCs exhibiting the broadest response profile (Fig. 6F, G) and the highest number of inhalation-modulated cells (Fig. 8B), but only small signal amplitude changes in response to odor concentration changes (Fig. 7C, D), at least in the concentration range that was tested here. One reason for the greater diversity in TC response properties might be that, according to our dual-tracing results, these contain fewer neurons with collaterals in both areas, which also corresponds well with previous reports demonstrating that TCs, in general, convey stronger stimulus-specific information^51^. TCs have furthermore been shown to form a more heterogeneous cell population^91^, receive stronger afferent input than mitral cells^110^, exhibit greater excitability and more irregular firing patterns^79^, and respond with more stable timing relative to sniff onset^41^. All these findings support the idea that TCs are responsible for the rapid distribution of information to olfactory cortical centers. Their wide range of firing patterns observed in in vitro recordings supports the notion that cells in this population have inherently different response properties, possibly due to differing projection targets. Our data furthermore show that TCs have more detectable sniff-triggered transients, suggesting that they might also convey mechanical signals that OB output neurons encode, stemming from blood pressure pulsations^111^ and airflow through the nose^112^.

(2) Another notable finding is that both AON-projecting mitral and tufted cells show less selectivity for different odors than APC-projecting populations. In this study, our odor set was limited and primarily comprised esters that have been shown to exhibit robust activation on the dorsal OB surface^56,113^. Even with this limited set of chemicals, significant differences in the selectivity of AON- and APC-projecting cells were readily detectable. This suggests that information is routed differently from the OB to the AON or APC, and that the AON might receive less information about odor identity than the APC does.

One possible interpretation is that the AON, with its strong, fast, and sniff-modulated inputs, serves as a first responder in odor detection in animals where specificity could be sacrificed for reliability and speed. The first sniffs of an incoming odor might then trigger responses in the animal, such as an increase in sniffing (i.e., sampling rate), if warranted. This would also align with the findings of Schiltz et al., who report that AON neurons exhibit poor odor specificity but respond strongly to novel stimuli^114^, and with the hypothesis that the AON might function as a tool for olfactory navigation^19^. The APC that receives more specific odor-related information would then be responsible for fine discrimination and valence coding, tasks that require such information.

The OB output streams investigated in our study not only carry olfactory information to different cortical areas but also receive feedback modulation from both AON and APC. Top-down projections from AON to the OB are more odorant-specific than those of APC^51,108,115^. But how does the AON support stimulus-specific feature projections to the OB if it receives broadly tuned input?

Broad, unspecific input to the AON does not imply that the backprojections to the OB must be equally unspecific. AON-derived projection fibers likely project to similar glomerular columns from which they receive information, as has been shown for bilateral connections^116^ and odorant-activated signals in AON fibers on the level of the OB are also centered around those OB areas that are naturally activated by this respective odor^108^.

Although we cannot rule out that differential odor processing is influenced by backpropagation, we think it might play only a minor role in this experimental setup, and that the effects shown here are due to intrinsic cellular heterogeneity. The reason is that both AON- and APC-derived cortical fibers are glutamatergic but exert a predominantly inhibitory effect on the OB network^109,117^. This requires activation of the OB’s inhibitory network, which exhibits markedly reduced excitability in the anesthetized animal^56^.

In the awake animal, however, AON-derived activity, backcoupled precisely to previously activated areas, is therefore potentially responsible for dampening ambient odors to increase signal-to-noise or improve gating of behaviorally relevant odorants. The AON’s potential speed and reliability advantage might allow modulation to occur well before processing in the APC can begin. Simultaneous recordings of bottom-up and top-down information might shed light on the respective timing of these pathways.

In summary, this study extends the current hypothesis of parallel processing in olfaction by showing that OB output streams are divided not only by cell type but also by the projection target. Target-defined olfactory bulb output populations exhibit distinct odor-coding properties, likely enabling rapid processing of complex olfactory signals. Moreover, our study provides the first evidence of target-dependent routing of sensory information prior to cortical processing.

## Materials and methods

### Animals strain and care

All experiments were conducted according to national and institutional guidelines for the care and use of laboratory animals, the rules laid down by the EC Council Directive (86/609/ECC) and German animal welfare. Study protocols were approved by IACUC Protocol Number 10-07011 (University of Utah), LANUV (84-02.04.2015.A260), the Government of Sachsen-Anhalt (203.m-42502-2-1833). Experiments were performed on adult PCdh21-Cre mice^57^, The GENSAT Project^118^, Stock #030952-UCD (Mutant Mouse Regional Resource Centers) and PCdh21-Cre crossed to the Ai95D GCaMP6f reporter line (JAX Stock No. 028865)^119^ of both sexes and homozygous for the Cre-expressing allele. Animals were housed under standard conditions in ventilated racks. Food and water were available *ad libitum*.

### Viral vectors

Viral vectors were obtained from the University of Pennsylvania’s viral vector core or Addgene. Vectors were from stock batches available for general distribution. pAAV.Syn.Flex.GCaMP6f.WPRE.SV40 (Addgene viral prep # 100833-AAV1; http://n2t.net/addgene:100833; RRID:Addgene_100833) and pAAV.Syn.Flex.NES-jRGECO1a.WPRE.SV40 (Addgene viral prep # 100853-AAV1; http://n2t.net/addgene:100853; RRID: Addgene_100853) was a gift from Douglas Kim & GENIE Project. Injection of Cre-dependent vectors (AAV1.Syn.Flex.GCaMP6f.WPRE.SV40 (*GCaMP6f*), AAV1.Syn.Flex.NES-jRCaMP1a.WPRE.SV40 (*jRCaMP1a*) was targeted to the AON or APC using stereotaxic targeting (AON +2.8 AP, ±1.25 ML, −2.8 DV; APC +2.35 AP, ±1.5 ML, −3.125 DV, relative to Bregma in mm) using previously described procedures^55,56,117^. Virus (0.5 μl, with titers in the range of 2.5–4.2 × 10^12^ gc/ml) was delivered through a 26 or 30-gauge metal needle at a rate of 0.1 μl/min. Mice were between 4 months and 1 year of age at the time of virus injection and were individually housed for at least 21 days before evaluating transgene expression or recording.

### Olfactometry

Odorants were presented as dilutions from saturated vapor (s.v.) in cleaned, humidified air using a custom olfactometer under computer control^68,120,121^. Odorants were presented for 4 s, with odor onset preceding the inhalation of the artificial sniff. This way, odor onset and inhalation onset/frequency are precisely timed, allowing precise averaging across animals without the need for e.g., “time warping”. All odorants were obtained at 95–99% purity from Sigma-Aldrich and stored under nitrogen/argon. The concentration of the odorants ranged from 0.1% to 4% (s.v.). The following monomolecular odorants were used: methyl valerate (MV), sec-butyl acetate (secba), isoamyl acetate (IAA), ethyl butyrate (EB), butyl ethyl ether (BEE), 4-Methylanisole (4MA), Triethylamine (TEA), vinyl butyrate (VB), isopropyl butyrate (IPB), methyl hexanoate (MH), ethyl tiglate (ET), 2-hexanone (2HEX), hexanal (HEX), propyl butyrate (PB) isopropylamine (IPB).

### Widefield calcium imaging

In vivo, widefield calcium imaging was performed as described previously^56^ with several modifications. Briefly, for acute imaging experiments, mice were anesthetized with pentobarbital (50 mg/kg), and the body temperature was maintained at 37 °C. To control stimulus sampling during anesthesia, an artificial inhalation paradigm using a double tracheotomy was performed^67,74,75^. A sniff tube was inserted into the trachea and directed to the nasal cavity to control stimulus sampling. A second breathing tube was directed to the lungs to allow for free breathing. During odor stimulation in imaging experiments, sniffing was triggered at 1 Hz with a sniff duration of 150 ms. Animals were head-fixed in a stereotaxic device (Kopf Instruments). The bone overlying both olfactory bulbs was thinned, and epifluorescence signals were acquired through the thinned bone covered with a coverslip and Ringer’s solution.

The optical setup for widefield microscopy included an Olympus BX51 illumination turret, a Zeiss objective Fluar 5x/0.25 NA x (∞/0.17), a filter set optimized for GFP (exciter: HQ480/40, dichroic: Q505LP, emitter: HQ535/ 50; Semrock), and a 470 nm LED source (Thorlabs). Optical signals were acquired at a 512 × 512-pixel resolution (4× on-chip binning) and a frame rate of 25 Hz and digitized at 14-bit using a scientific CMOS camera (PCO edge 4.2.LT, PCO), then synchronized with other experimental signals (respiration, odor control) and stored to disk using LabVIEW (National Instruments).

### Epifluorescence data analysis

Basic processing and analysis of optical signals were slightly modified from protocols described previously for epifluorescence imaging^56^. Briefly, initial data processing included extracting fluorescence time courses from visually selected regions of interest (ROIs). Epifluorescence traces were smoothed with a low-pass Gaussian kernel with a 6–8 Hz cut-off. The pseudocolor topographic activation maps reflect single trials in which the movies were spatially filtered using a Gaussian window of 0–2 and a bin size of 10. For display in the figures, topographic odor maps were normalized to their maximum values; the maximum Δ*F*/*F* values are indicated below each map. Bilinear interpolation was applied to double the pixel resolution. For autocorrelation analysis, maps were demeaned, and the resulting autocorrelations were normalized to their peak amplitude. Autocorrelation widths were determined as the average diameter of a contour line drawn at 75% of the maximum value to generate estimates consistent with previously reported values^56,68^. To explore potential differences in topographic response maps across odors, consensus topographies (contour plots)^68^ were calculated and compared between virally traced and transgenic mice. Contour plots show consensus topographies, i.e., they reflect the most reliably activated areas across several animals. Consensus topographies were depicted for four odors: EB, IAA, secba, and MV. Response maps were smoothed with a 6 × 6 pixel (52 µm) mean kernel to generate these topographies and aligned across preparations using the caudal sinus and midline of the dorsal bulbar region^68^. Each map was thresholded at 40% maximal amplitude, further smoothed (Gaussian kernel SD of 12 pixels), normalized in amplitude, and responses to the same odorant averaged across preparations. Only mice with a broad GCaMP6f expression across the dorsal OB were included in this analysis.

### 2-photon calcium imaging

Animals were prepared for 2-photon imaging in the same manner described for widefield imaging experiments, except that a craniotomy covering the dorsal surface of the OBs was performed and covered with saline or electrode gel (Dahlhausen & Co. GmbH). 2.5% low melting point agarose was applied directly to the brain surface to reduce movement artifacts if necessary. Imaging was performed with a 2-photon microscope (MOM; Sutter Instruments) coupled to a pulsed Ti: Sapphire laser (Mai Tai HP; Spectra-Physics) and controlled by Scanimage^122^. High-resolution imaging was performed using a 16x/0.80 NA objective (Nikon, LWD, DIC N2, ∞/0, WD 3.0), and the emitted light was collected by gallium arsenide phosphide (GaAsP) photomultiplier tubes (R6357; Hamamatsu). Bandwidth filters with ranges of 535 nm ± 50 nm and 610 nm ± 75 nm (Semrock) were used. Cell bodies were visualized at depths ranging from 150 to 500 µm. Fluorescence videos were acquired at 7.5 Hz in most experiments or at 3.76 Hz, and z-stacks were recorded at 1.88 Hz or 0.47 Hz. Fluorescence time series were extracted and analyzed with custom MATLAB scripts. Due to the low baseline values of GCaMP6f, odor response amplitudes for 2-photon imaging were calculated as *∆F* rather than Δ*F/F* to avoid aberrant amplification (similar to ref.^123^). A series of eight odors was used to test the odor selectivity of individual cells. An increasing concentration series (0.1%, 0.25%, 0.5%, 1%, 2%, 3%, and 4% s.v. odor) was applied to test the concentration sensitivity of individual cells. GCaMP6f responses to single presentations of each stimulus were imaged and measured. All cells within a single field of view were marked using maximum-projection maps. As standard deviations of AON and APC traced neurons were not significantly different from each other (23,2 (AON) vs. 24,3 (APC), *p* = 0.950 Mann–Whitney U test), responses were considered significant if the odor-evoked fluorescence change exceeded two times the standard deviation of the pre-odor baseline in either positive or negative direction. Only cells showing a significant response to at least one odorant were analyzed further.

In part of the analysis, cells were classified as MCs or TCs based on imaging depth, cell body morphology, and appearance in higher-resolution z-stacks of the imaging area (similar to refs. ^50–52,80^. Cells were classified only according to these criteria, without knowledge of their response patterns.

### Sniff modulation analysis

Sniff kinetics (amplitude, latency, rise time, fall time) relative to inhalation were calculated as described previously for optical signals in rats using a custom algorithm that fit the optical signals to a double-sigmoid function^56,76^. Latency was defined as the time from the start of the inhalation, as reported by an upward deflection in the pressure signal, to the time the fitted optical signal reached 10% of its maximal amplitude (t10). Time from 10% to 90% of maximal amplitude was defined as rise; time from 90% to 10% as fall time. For descriptive statistics, values were measured from multiple foci and odorants at the first inhalation of each trial. To compare anteromedial versus caudal-lateral dynamics, values were calculated for the second to fourth odorant inhalation. Measurements were made for multiple ROIs in each domain and averaged to obtain a single value per domain. Relative values between the domains were plotted and compared using a paired t-test. Traces were classified as significantly “sniff modulated” if two criteria were met: (1) Fluorescence trace fluctuations were considered as odor modulated if the maximum amplitude during odor presentation was 1.5 times higher than in the pre-odor period. (2) Fluorescence trace fluctuations were considered as sniff modulated (as opposed to a tonic response) if the relative sniff amplitude, defined as the signal amplitude following the sniff divided by the amplitude before the sniff, was less than 1.1. For the analysis, we excluded the first sniff following odor application. All analyses were performed using custom-written software in MATLAB or LabVIEW. All statistical tests were performed with MATLAB, Origin, Excel, or JASP and were two-sided, unpaired t-tests unless stated otherwise. Summary data are reported as mean ± SD unless noted otherwise.

### Lifetime and population sparseness

Lifetime sparseness is a measure of the non-uniformity of the distribution of responses to different odors for a cell (e.g., how selective a single neuron responds across many stimuli), whereas population sparseness measures the distribution of cell responses for an odor (e.g., how many neurons respond to a stimulus)^124^. Lifetime sparseness was calculated similarly to Bolding and Franks^20^.

$${{\rm{S}}}=(1-({(\sum {{{\rm{r}}}}_{{{\rm{i}}}})}^{2}/\sum ({{{\rm{r}}}}_{{{\rm{i}}}}^{2})))/(1-1/{{\rm{n}}})$$

where *r*_*i*_ is the response to the *i*th odor and *n* is the number of odors. Values near 0 indicate that all odorants uniformly activate a given cell, whereas values near 1 indicate a high degree of odorant selectivity. Population sparseness for each odor was calculated with *r*_*i*_ as response of the *i*th cell and *n* as the number of cells. Values near 0 indicate uniform activity across all elements, and values near 1 indicate a lack of activity in most elements.

### Mutual information

Mutual information was calculated using a nearest-neighbors method for both the continuous versus continuous variable^125^ and for the continuous versus discrete (binary, categorical) variable^126^ using the MATLAB Mutual Information Package provided by John M. O’Toole (2026). Mutual information (https://github.com/otoolej/mutual_info_kNN/releases/tag/v0.1.1), GitHub). There is one discrete variable (AON or APC) and multiple continuous variables (fluorescent signal at different concentrations).

### Support vector machine (SVM)

We used features (amplitude, rise time, latency, fall (decay kinetics) extracted separately for each sniff from the fitted 2-photon calcium imaging data (see section “Sniff modulation analysis”) as input parameters for the classifier analysis, comprising 36 sniff-features (4 features × 9 sniffs). Only cell odor pairs with a successful fit were incorporated into the classifier analysis. To measure sniff-feature importance, we employed feature selection algorithms. A filter-type feature selection algorithm (we used a univariate feature ranking for classification using chi-square) measures feature importance based on the characteristics of the features, such as feature variance and feature relevance to the response (available in MATLAB Statistics and Machine Learning Toolbox). This filter-type feature selection is uncorrelated to the training algorithm. Our data pool contained 1431 cell odor pairs (AON mitral 468, AON tufted 287, APC mitral 512, APC tufted 164). The data was downsampled to match the number of data points in the smallest category. Downsampling was repeated 100,000 times (bootstrapping), each followed by feature ranking. Cross-validated accuracy was calculated to evaluate classification performance. We split the downsampled data into training (75%) and test (25%) sets, maintaining a 1:1 category distribution (fivefold cross-validated, 100 repetitions). All features were used to train the SVM. We report mean test accuracies of the test dataset for both original and random categories.

### Histology

In all experiments, we evaluated viral expression (GCaMP6f, jRCaMP1a, GCaMP6s) in OB cells using post-hoc histology. Briefly, mice were deeply anesthetized with an overdose of sodium pentobarbital and perfused with 4% paraformaldehyde in PBS. 100–200 µm-thick tissue sections were evaluated from native fluorescence without immunohistochemical amplification with a Leica TCS SP2 confocal laser scanning microscope at 10x or 20× magnification. The fluorescence intensity of confocal images was analyzed and plotted in ImageJ. All mice that underwent imaging were perfused after the experiment and histological sections were collected to confirm the correct injection target. This was confirmed by following the needle tracts (Supplementary Fig. 1), as there are no Pdh21-expressing cells within AON or APC that could have provided orientation. Only mice showing correct AON or APC targeting were used for analysis.

### Statistics and reproducibility

Data reproducibility was ensured by conducting experiments in a number of animals (see Results and Figure legends). A representative experiment is shown for all data presented. Mice were randomly selected for injection sites. All data were reanalyzed blind to the experimental condition.

We analyzed the amplitudes of calcium transients from OB output neurons in widefield and single-cell 2-photon recordings to compare odor signals in output neurons projecting to AON versus APC. The normality of calcium transient amplitudes or kinetic parameters was assessed analytically with the Shapiro–Wilk Test and graphically with distribution and Q-Q plots. Mann–Whitney U tests were used to compare unpaired data. Wilcoxon signed-rank tests were used for the paired sample test.

To compare more than two groups, the Kruskal–Wallis test was used, with subsequent Dunn’s Post-Hoc analysis if the Kruskal–Wallis test was significant.

Values used for statistical analysis of calcium signals were either *ΔF* fluorescence values or calculated as the area under the curve (AUC). Boxplots depict the median, interquartile range, minimum to maximum range, and outliers. Statistical analysis, tables, and boxplots were performed and generated with the open-source JASP software, MATLAB, or Origin. Boxplots visualize median value, 25 and 75% quartiles, interquartile range (IQR), i.e., 75% - 25% quartile values, and maximum and minimum values plotted with outliers excluded. Significance is given as *p* < 0.05 *, *p* < 0.01 **, *p* < 0.005 ***. Figure 8G and Supplementary Fig. 2C, D were created using the “boxplot” MATLAB function; the central mark indicates the median, and the bottom and top edges of the box indicate the 25th and 75th percentiles, respectively. The whiskers extend to the most extreme data points, excluding outliers, and outliers are plotted individually using the ‘+‘ marker.

## Supplementary information

Supplementary Information

Supplementary Data

Transparent Peer Review file

## Data Availability

All numerical data that is represented in the figures (Figs. 1–8, Supplements) available as source data file corresponding to the figures (Supplementary Data 1). All other data are available from the corresponding author on reasonable request.

## Code availability

No unique software or complex algorithms were designed for this project. However, the custom MATLAB scripts used for data processing and analysis are available from the corresponding author upon reasonable request.
