# Supplementary material for: Target-dependent routing of sensory information in the olfactory system
Source: Commun Biol. 2026 Oct 7;9:1306. doi: 10.1038/s42003-026-11071-5 (PMC13646270; doi:10.1038/s42003-026-11071-5)
Supplement: Supplementary file 1 [file 42003_2026_11071_MOESM1_ESM.pdf]

Supplementary Information

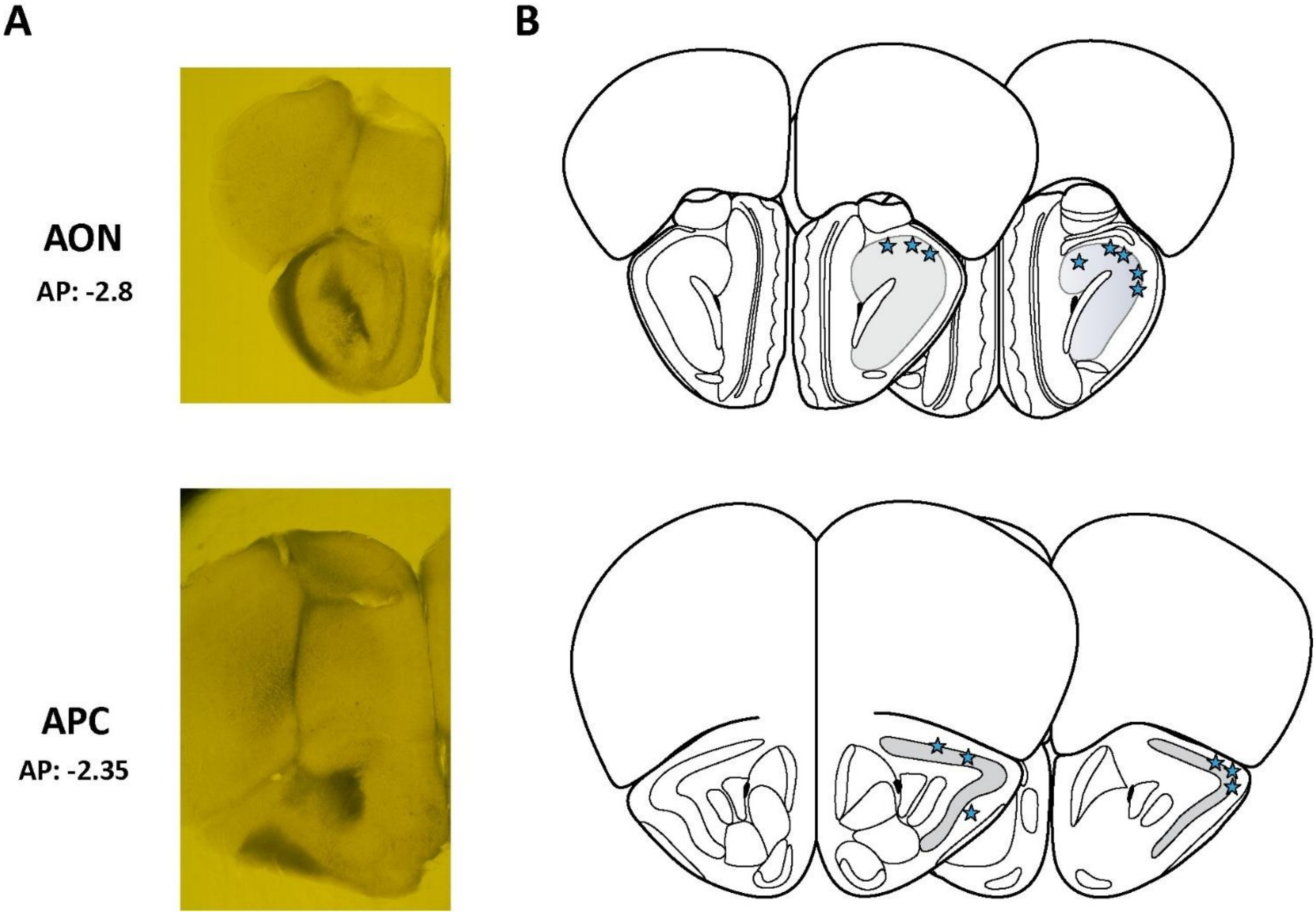

Supplementary Figure 1: Injection sites for AON and APC. **A** Example histological sections of AON (top) and APC (bottom) injections. **B** Reconstruction of injection sites in all animals included in the analysis.

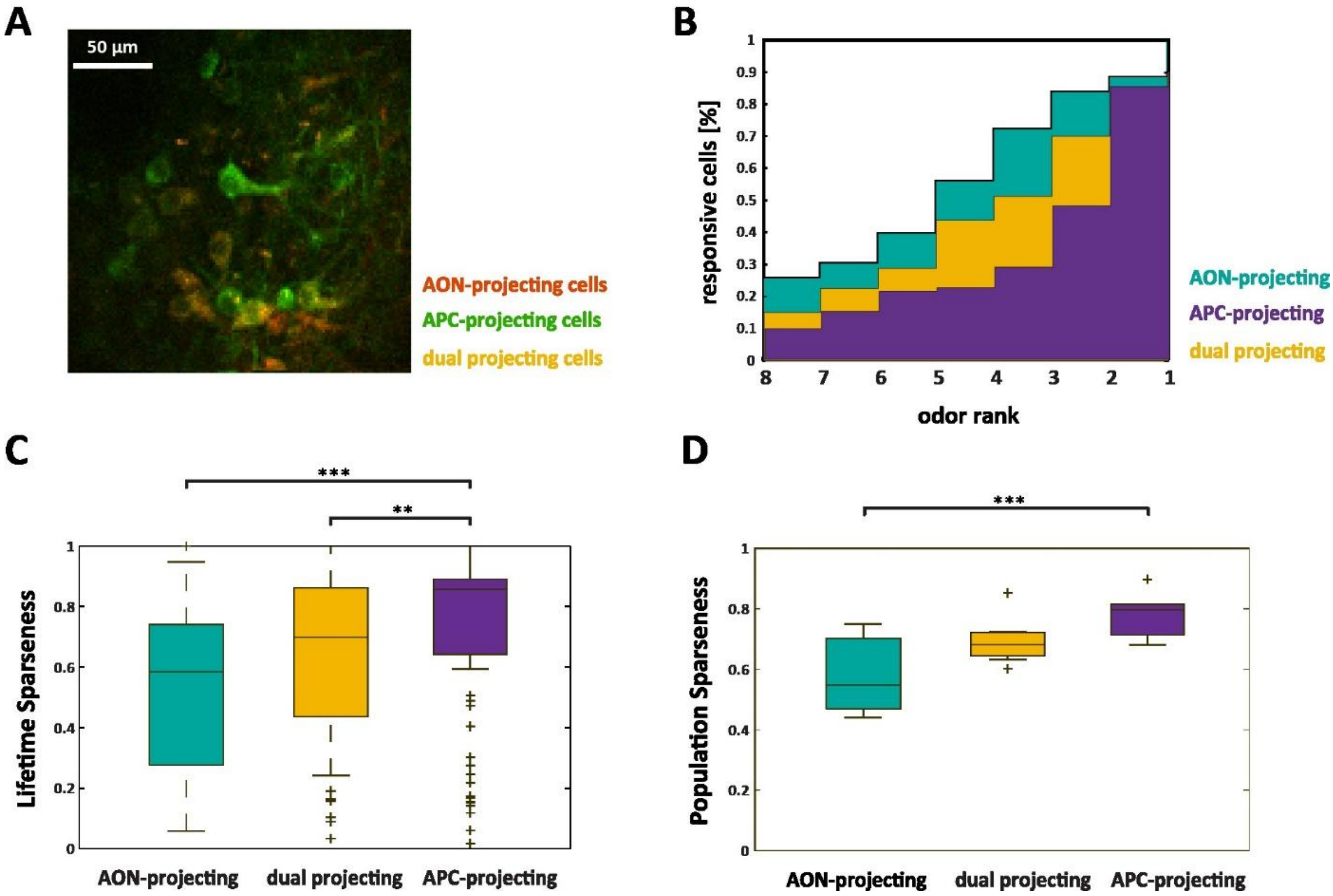

Supplementary Figure 2: Odor selectivity properties of dual-projecting cells. **A** Example OB of a mouse injected with a green fluorescent marker protein into APC and a red fluorescent marker protein into AON. **B** Cumulative probability plot indicating the fraction of responsive cells in dual tracing experiments. Dual-projecting cells exhibit a mixed odor-response behavior that falls between AON- and APC-traced cells. **C** Lifetime sparseness analysis for AON-projecting, APC-projecting, and dual projecting MTCs. **D** Population sparseness analysis for AON-projecting, APC-projecting, and dual projecting MTCs. Central mark = median, box edges = 25th and 75th percentiles, whiskers extend to the most extreme data points excluding outliers; '+' denotes outliers.

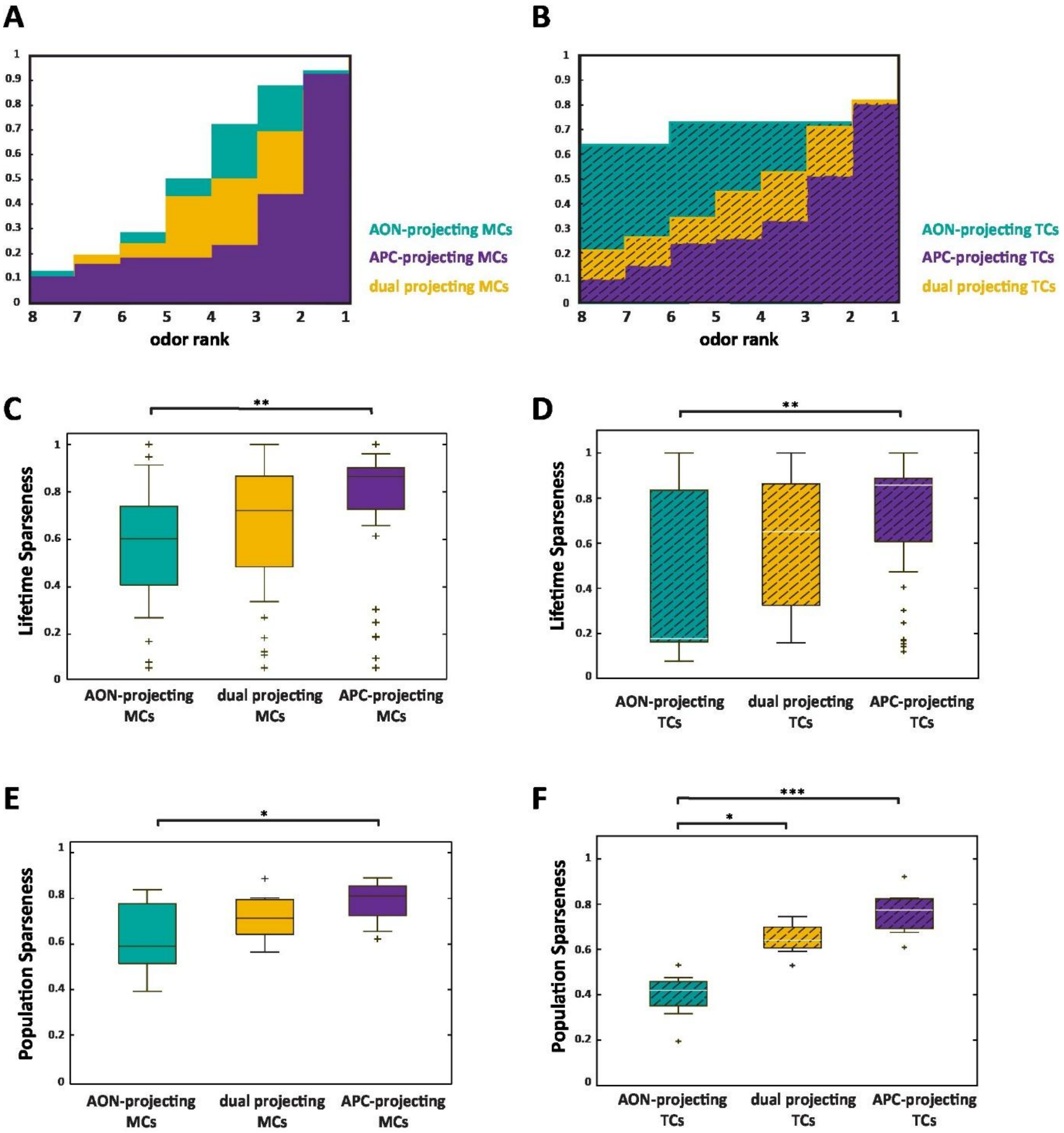

Supplementary Figure 3: Odor selectivity properties of AON-, APC-, and dual-projecting MCs and TCs. **A, B** Cumulative probability plots indicating the fraction of responsive MCs (**A**) and TCs (**B**) separately in dual tracing experiments. Dual-projecting cells exhibit a mixed odor-response behavior that falls between AON- and APC-traced cells. **C, D:** Lifetime sparseness analyses for AON-projecting, APC-projecting, and dual-projecting MCs (**C**) and TCs (**D**). **E, F** Population sparseness analyses for AON-projecting, APC-projecting, and dual-projecting MCs (**E**) and TCs (**F**). Central mark = median, box edges = 25th and 75th percentiles, whiskers extend to the most extreme data points excluding outliers; '+' denotes outliers.
