# Supplementary material for: Target-dependent routing of sensory information in the olfactory system
Source: Commun Biol. 2026 Oct 7;9:1306. doi: 10.1038/s42003-026-11071-5 (PMC13646270; doi:10.1038/s42003-026-11071-5)
Supplement: Supplementary file 2 [file 42003_2026_11071_MOESM3_ESM.pdf]

nature portfolio

Peer Review File

Target-dependent routing of sensory information in the olfactory system

Corresponding Author: Professor Markus Rothermel

This file contains all reviewer reports in order by version, followed by all author rebuttals in order by version.

Version 0:

Reviewer comments:

Reviewer #1

(Remarks to the Author)
Review of Target-dependent routing of sensory information in the olfactory system by Kim et al.

This manuscript addresses a key question about olfactory processing. Namely, given the view that different cortical regions process distinct aspects of the sensory stimulus, the authors asked if these differences are due to the properties of the projection cells, rather than local processing at the target. Using viral tracing from two different regions (AON and APC) the authors could separate between neurons projecting to each of these regions, and also, by analyzing cell body locations, whether these are mitral or tufted cells. The experiments are complex and well-designed and the data would be very valuable to people in the field.

The authors conclude that there are differences between AON and APC projecting neurons, with the former being less selective and less "concentration dependent". These are important conclusions. However, these differences are rather subtle qualitative differences, and I feel that describing these as distinct channels of sensory information is misleading. Furthermore, it is not clear how they are related to the distinct functions proposed for the two target areas. Another major issue, is that that claims about these differences are made with little statistical justification. Finally, I think that the manuscript could be more accessible to, by focusing on the major points and better explaining and illustrating some of the results. More specific comments are given below.

My comments appear in the order that they came up, rather than importance. The major comments are marked with an *.

General issues for consideration:
* 1. According to the tracing analysis, a large fraction of cells target both areas. In fact, I would expect this to be an underestimate of the extent of overlap. This weakens the argument of separation into different streams. More importantly, it could explain why the TCs – which show less overlap – have more distinct features for subpopulations projecting to each of the two regions. Please address this issue in the text.

* 2. As mentioned above, the differences between cells with distinct projection targets are not huge. One explanation may be that there are other, perhaps more dramatic, differences, which cannot be detected with the current analyses, and particularly, the rather small stimulus set used. Another possibility is that the differences are in realty rather subtle. I think that this point should be addressed.

General data analysis issues:
1. Was there only one repeat pers stimulus? This should be clarified.

2. The authors show that response selectivity cannot be easily accounted for by maximal signal strength. Isn't the more relevant metric "signal to noise"? With S/N I mean variability as evaluated without a stimulus, and the strength of the response. Using this measure, I would ask if there is correlation between S/N and selectivity.

3. Other than controlling rate and depth of breathing, I don't see the benefit of the rather complex tracheotomy procedure. Could this form of (retro-nasal, if I understand correctly) breathing impact the sniff-locked responses? I feel that this point

should be addressed/discussed.

4. Please clarify how Ca imaging data was up-sampled. What algorithm was used.

5. You write: “We employed a univariate feature ranking method for classification, utilizing chi-square tests to assess feature importance based on the characteristics of each feature, including variance and relevance to the response”. What decisions were made based on this ranking? More generally, the description of the SVM analyses (and the point of Figure 8F) lack in detail and are not clear.

6. In figure 1D, I really cannot see the overlap and how the shaded regions match what is seen in the upper plots

*7. It seems that the conclusion regarding different selectivity as a function of target region is based on visual inspection of Figure 3C. There are no statistics as far as I can judge. I would expect some more quantification, including a comparison of distributions of lifetime sparseness of neurons from different classes. Also, while cumulative frequencies are informative, they make it harder to grasp the general form of the distribution. Please include the non-cumulative distributions (how many neurons responded to any number of stimuli) in this and in similar plots.

8. The conclusion in the statement “AUC of rank-averaged odor response amplitude for APC- and AON-traced MTC was not statistically different for the two populations (Fig. 3D, AON-traced AUC 881,12 ± 615.93 (mean ± SD); APC-traced AUC 841.96 ± 736.58; p = 0.06 Mann-Whitney U Test), further demonstrating no major impact of signal amplitudes.” Is not clear to me. As part of this, the phrase “AUC of rank-averaged odor response” is not self-explanatory and leaves the reader guessing. What exactly are the rank averaged odor responses?

9. Concerning the statement: “A similar fraction of AON- and APC-traced MTCs responded significantly to at least one odor concentration”, is this for one specific odorant or across all odorants? I think that this distinction is significant, because the number of effective odors differs for the two areas, as shown here.

*10. Re the statement: “When calculated on a cell-concentration basis, AON traced MTCs exhibited a significantly lower fraction of significant responses compared to APC traced cells”, Doesn’t this argue for higher concentration selectivity for AON cells? If the fraction of responding cells across regions is similar, but there are more cell odor pairs for APC, then this implies less concentration selectivity for the APC. No?

* 11. A key observation (Figure 4), which is the basis for one of the main conclusions here is that: “APC-traced cells reacted far more strongly to an increase in odor concentrations”. However, if you scale the two regions in Figure 4F to the same maximum value, is there still a difference in the form of the dependence? No doubt that there is a difference in maximum amplitude, but this may be just a difference in scale. Here too, I think that concentration dependence should be analyzed and compared more rigorously.

* 12. connected to the previous comment: The non-monotonic concentration response of AON cells is not discussed. This could certainly account for the contrast between the number of cells and the number of cell-concentration pairs. Right?

13. Classification analysis: “In comparison, a classifier trained on a dataset with randomized categories only predicted 495 samples correctly, achieving an overall accuracy of 35%, which demonstrates the information content contained in the true categories.” Isn’t chance performance 25%? Please clarify this point.

* 14. “APC-traced TCs reacted the strongest to an increase in odor concentrations”. This is not at all apparent from Figure 7D. From that figure, all populations seem similarly concentration dependent.

Presentation issues:
* 1. Why do we need to go through the first part (without separation into cell types) before looking at separate populations? The first part seems somewhat redundant with the second, which is more informative. Why not start there?

2. Some of the labels and the colors make it quite hard to make quick sense of the data. It would help to have a specific color for each cell type, and a different line style for different regions. A case in point is Figure 6C, with confusing labelling. I suggest to add labels across the “columns” and maybe also right next to pie chart slices, to facilitate the interpretation of the charts.

3. Although the differences between cell populations are not huge, it would be nice to try and link the observed differences to known function of the projection regions. If there is no obvious link this is ok too, but please at least address this issue.

4. A reference or more detail about the deep learning model is needed.

5. When discussing classification results, please define precision and specificity

6. I don’t understand the “(as might be expected)” comment on line 591. If it is obvious, then why say it. If it is not obvious, then explain why this is expected.

7. I cannot follow the logic of the discussion in lines 809-814. The argument about specificity seems quite vague. Please clarify.

8. In Figure 4A, please indicate which cells correspond to these traces? (that is, projection region and subtype)

9. “concentration-averaged odor response amplitude for both APC- and AON-traced MTCs significantly increased with odor concentration”. I expected that concentration averaged means an average over concentrations. I believe this is just an issue phrasing, but please clarify.

Minor:
1. In “(see chapter “Sniff modulation analysis”) – change chapter to section

2. The sentence beginning on line 388 (“To display topographic …”) is a bit hard to read. Consider rephrasing.

Reviewer #2

(Remarks to the Author)
In this article, the authors discuss the target-dependent routing of sensory information in the olfactory system, focusing on the differences in output from the OB to the two olfactory cortical areas and their implications in odor information processing. Authors have utilized retrograde labelling from the anterior olfactory nucleus (AON) and anterior piriform cortex (APC) to differentiate the OB output neurons. They utilised in vivo 2-photon calcium imaging to reveal that AON-projecting MTCs have lower odor selectivity and concentration dependence compared to APC-projecting MTCs. Further, their results show APC-projecting TCs displaying narrower tuning and stronger concentration dependence than AON-targeting TCs. This study provides interesting insights into how variable the OB output could be while considering four separate output streams. Another interesting result is the differential ‘sniff’ dependency of the AON-MTCs and APC-MTCs. These are important results considering the increasing evidence on the multimodality of olfactory information processing. Experiments are carefully designed and analysed, which is commendable. The MS can be improved by adding some missing experimental evidence and making the discussion broader.
I have the following suggestions:
Major concerns:
1. As the authors used the retrograde labelling and the target areas are nearby, it’s important to show the evidence on the specificity of injection sites. Please show some examples proving the specificity of injections. This is critical for dual tracing experiments.
2. In lines 240-245, the authors explain the selection criteria. Was this applied in all experiments? Please justify this in the context of narrow vs. broader tuning properties.
3. Did authors observe external tufted cells projecting to the APC/AON? Are there any observations of synapse-crossing with the viral vector that the authors have used?
4. Figure 1D analysis: “more noticeably diffuse component” – Do authors expect any artifacts due to viral transduction in the experimental groups? Please increase the ‘n’ of control group to 5-6.
5. Lines 441 - 442: “Significant inhibitory responses were observed only in 2.5% of AON-traced neurons and none in APC-traced neurons. Due to the low number of inhibitory responses, further analysis was conducted only on excitatory cell-odor pairs (COPs)”. How did authors classify inhibitory responses? Please explain how the authors delineated excitatory and inhibitory responses. Please discuss the inhibitory action.
6. Line nos. 380 and 401: Why are the animal numbers different for the AON group?
7. Line no. 330 says “Only mice showing correct AON or APC targeting were used for analysis.” – Please explain the exclusion criteria.
8. Authors declare that AON-traced cells were predominantly located in the external plexiform layer whereas APC-traced cells are primarily found in the mitral cell layer. Please discuss the observed differences in odor selectivity in detail in the context of differences in their arborization, connectivity with neighbouring neurons, channel density, and the inhibitory feedback (Burton and Urban 2014, Fukunaga 2012).
9. Please elaborate on the discussion of correlation between breathing dynamics and the OB output, considering the recent reports of multimodality in the olfactory system (Salameh et al., 2024 Science; Mahajan et al., 2024 bioRxiv)
Minor points:
1. The age group of experimental mice is very variable (4 weeks to 1 year) – Please explain
2. It would be great to add the PID traces, as the authors used varying concentrations ranging from 0.1% to 4% (s.v.).
3. Line 383: Presynaptic OSN expression of GCaMP6f – please clarify

Reviewer #3

(Remarks to the Author)
I co-reviewed this manuscript with one of the reviewers who provided the listed reports. This is part of the Communications Biology initiative to facilitate training in peer review and to provide appropriate recognition for Early Career Researchers who co-review manuscripts.

Reviewer #4

(Remarks to the Author)

Title: Target-dependent routing of sensory information in the olfactory system

Summary
Overall, this study makes a valuable contribution in identifying differential response properties of MCs and TCs according to their projection targets (APC vs AON). This raises the interesting possibility that specialized parallel channels may already be present at the level of OB outputs for conveying odor information to the olfactory cortex. The authors make use of an impressive suite of tools to offer mechanistic insights into odor coding, and the results are potentially important and of broad interest. However, the paper would be strengthened by analytical and text revisions to make the results more clear.

Major Comments/Concerns

1. The introduction frames the role of cortical feedback versus feedforward segregation as a dichotomy,and argues that the evidence presented in this paper favors a model in which OB outputs constitute independent information streams defined by cell type and projection target. However, this largely ignores the contribution of cortical feedback in differentially regulating the activity of projection cell types. In fact, studies (e.g. Chae et al. 2023) have shown that feedback from APC and AON selectively restructures the responses of its dominant projection type (TCs for AOn, MCs for APC). Without directly recording the activity of these corticofugal projections or employing manipulations to disrupt these projections, it is difficult to assess the degree to which the distinct properties of APC vs AON projecting MTCs are shaped by the functional properties of the MTCs themselves vs differential contributions of feedback projections from the different cortical targets. The authors should discuss what is known about AON/APC feedback on MTC activity under anesthesia, whether feedforward and feedback streams maintain parallel identities, and how feedback could influence the tuning differences reported here.
2. The authors introduce this study under the premise that parallel processing streams of sensory stimuli leads to faster processing. However, the authors do not provide evidence nor discussion about whether their findings would support faster processing or the behavioral consequences of such coding mechanisms. This study could benefit from additional comments on this issue.
3. Dual-color tracing (Fig. 5B–C) reveals that 30–50% of MTCs collateralize to both AON and APC. However, it remains unclear how these hybrid cells contribute to identity and concentration coding. For example, it would be interesting to note whether decoder performances change when excluding dual-projectors. A deeper analysis of their functional identity—e.g. whether they resemble mitral- or tufted-type kinetics—and how these properties align with the projection-specific parallel processing streams that the authors describe, would be helpful. Furthermore, the authors should clarify if this dual-projecting population was included both in the APC-projecting and AON-projecting analyses of MCs and TCs.
4. The pooled analyses in Figures 1–4 compare AON- versus APC-projecting populations that differ substantially in their MC/TC ratio (as confirmed in Figure 5C). Since it is well-established that MCs and TCs have distinct tuning properties, these differences may primarily reflect cell-type composition rather than target-specific coding. Given the centrality of this cell type distinction, it should be introduced more explicitly and earlier in the text. Additionally, the magnitude of this disparity calls into question the value of the analyses in Figures 1-4, which fail to control for projection cell type identity. The authors should attempt to quantify how much of the observed AON/APC differences can be explained by relative cell-type fractions, and discuss the implications for their pooled analyses.

Minor Comments/Concerns
1. A significant fraction of the analyses in the paper rely on computing the fraction of units which are significantly odor responsive. The methods define "significantly odor responsive" as exceeding two standard deviations of a pre-odor baseline, but do not specify the exact baseline window or the statistical criteria used to establish significance for cell-odor pair analyses. The authors should also assess how the population of responsive cells identified with criterion align with other standard measures of responsivity (e.g. auROC, Wilcoxon-rank sum analysis).
2. In Figure 1D, the authors note that viral GCaMP expression in target-defined output neurons yields a significantly higher diffuse signal in the OB than transgenic Pcdh21-GCaMP approaches. The authors should comment on how their retrograde viral strategy affects MTC coverage relative to other work.
3. It's unclear why the MTCs in figure 2B are grouped together, rather than separated, since the cells would've been imaged across different imaging planes. For clarity and ease of comparison in future figures, the authors should indicate which cell-odor pairs are MCs vs TCs. Similarly, it's unclear if the data in figure 6B is the sorted version of the data presented here. The authors should clarify.
4. Given the A-P axis differences observed in figure 1, the authors should clarify relatively where the 2P imaging took place.
5. In each figure legend, the authors should include the total numbers of cells per projection area included in the analyses to supplement the fractional metrics.
6. The authors should provide examples of the calcium traces returning to baseline in figure 2 so that the reader can get a better sense of the odor-response dynamics.
7. To supplement the cell-odor pair analysis in figure 2b, the authors should include a cell x odor-evoked responses for all odors so that the reader can visualize the response sparseness and variability for different populations more clearly.
8. A comment about the breathing trace is needed in figure 2 legend.
9. For all figures which include mean calculations, the authors should indicate how many cells were included (e.g. Figure 2F, 3E,7D etc.) and what the population sparseness is for both odor concentrations and odor identities. Alternatively, the authors could normalize by the minimum number of cells per group.
10. This study and others (including Zhang et al., 2025), have demonstrated variability in mitral and tufted cell responses, even for those that innervate the same glomerulus. Given the variability of cell responses, the authors should identify correlations between odor response vectors for all cells (sorted by cell type and projection region) so that the similarities and differences between cell class and projection region area apparent at a single cell level.
11. Line 451 – what was the concentration used in these experiments?
12. It's unclear what the authors mean by lines 462-463. Given the known differences in mitral and tufted cell properties,

comparing mean AUC for the combined population doesn't make sense – examples where the molecular identity slightly differs. The authors should clarify or delete this conclusion.

13. The metrics that the authors use to quantify the breadth of odor tuning in Figure 3 (fraction of MTCs responding to N odors) are difficult to interpret. Given that the authors report a greater overall fraction of odor-responsive AON-projecting cells than APC-projecting cells, metrics that do not rely directly on the fraction of responsive cells will be informative. The authors should supplement this analysis with additional metrics of odor selectivity (such as lifetime sparseness, response entropy, selectivity index, ROC-based measures), and/or odor identity decoding analyses.

14. The visualization of odor responsiveness in figure 3B is difficult to interpret. In order to understand how diverse the responses are across odors, the authors should include a figure that illustrates maximum dF per odor for all cells by projection target. This will better illustrate if all cells are responding to the same odor or a different set of odors.

15. Likewise, the metrics used to quantify concentration-tuning in Figure 4, which also rely on quantifying the fraction of responsive cells suffer from similar concerns. As the authors note, the fraction of responsive AON-traced cells is lower than the fraction of responsive APC-traced cells at every concentration. This fact obscures the effect of concentration-dependence on the fraction of responsive cells in Figure 4E. Additional analyses (e.g. mutual information, concentration decoding) should be included to supplement this analysis.

16. In Figure 4F, the response amplitude of APC-projecting MTCs is significantly greater than the response amplitude of AON-projecting MTCs over a range of concentrations. However, in Figure 3D/E, the authors report no significant difference in the response amplitude of APC vs AON-projecting MTCs. The authors should clarify the odor concentration used in earlier figures to explain this apparent disparity (in methods, results and figure legends), and comment on the significance of the difference in response amplitude at higher concentrations.

17. The data in figure 5C indicates that a significant proportion of AON-projecting cells were found in the EPL. However, in lines 553-555, the authors write that about 25% of AON and APC-projecting cells were TCs. The authors should explain this discrepancy.

18. The deep learning model that the authors use in Figure 5E is not particularly informative given its black-box nature. As the authors note, "the predictive features of this model are not easily extractable". Given that there are no significant differences between the population averaged calcium traces of TCs or MCs with different projection targets (Figure 5D), identifying the features that drive this above-chance decoding is particularly important. We encourage the authors to utilize another approach to estimate the features distinguishing the different output streams. For instance, the SVM that the authors use in Figure 8A, which explicitly includes features extracted from activity traces could be more informative.

19. The respiration-modulation analysis appears to use different response criteria for classifying odor-modulated cell odor pairs than the criteria for classifying odor-responsive cell odor-pairs used elsewhere in the paper. The authors should please clarify the rationale for this discrepancy

20. In Figure 8C, the authors show that AON-traced TCs display the greatest sniff modulation during odor presentation. However, as shown in Figure 8B, AON-traced TCs also display this greater sniff modulation during baseline, likely driven by stronger sniff modulation in TCs vs MCs overall. The authors should comment on the extent to which this effect is specific to AON-traced TCs during odor presentation, and provide additional analyses to support this conclusion.

21. In the discussion, the authors argue that "although only a minority of the TC population, the APC-projecting TCs convey the predominant part of information on odor identity and concentration to the olfactory cortex". This is a substantial claim, and difficult to support without direct recordings in APC. Additionally, while the authors propose that APC-projecting TCs may convey more odor identity information than APC-projecting MCs, this is not directly shown in the analysis.

22. The authors should cite Koldaeva et al., 2021 as a reference for distinguishing between the activity of mitral and tufted cells

23. To improve clarity, the authors should consider rephrasing "most unselective" to "broadest response" instead

24. Virus concentrations need to be clearly indicated in methods

25. Given that mitral and tufted cells generally are more active under anesthesia, the justification of dF instead of dF/F0 is not convincing. The choice to use dF (instead of normalizing it) makes the comparison of amplitudes between projection-specific MTCs difficult to assess (between GECIs, mice, cells at different imaging depths due to nonlinear excitation through the tissue). At a minimum, the authors should provide odor-evoked z-score or dF/F responses or justify the use of dF (e.g. provide examples of low baseline responses), which may be prone to artifacts from imaging depth, relative calcium concentration, etc. An additional experiment where GCaMP and jrCaMP are expressed by AON and APC-projecting cells within the same animal may serve as a more direct comparison of their response properties without the animal-dependent artifacts.

26. Correction: line 355 "sites" instead of "sides"

27. Citation needed: line 368

28. The set of odors hardly allows the authors to distinguish odor selectivity across a broad range of odorant classes. The authors should mention this as a potential caveat when interpreting the results about odor selectivity across processing streams.

29. The authors should convert their "selectivity" measure into lifetime sparseness so that it is more easily comparable to other studies on the OB and olfactory cortex.

30. It's unclear in the tuning curve calculations if the authors accounted for differences in the number of imaged mitral and tufted cells by regional projections (see lines 624-626). Since it's unclear if the tuning curves would be dominated by the most numerous cell type, the authors should provide additional evidence or account for this potential artifact.

31. Lines 641-642 – is it possible that APC-projecting TCs respond to a narrow range of concentrations but that their sensitivity is higher? Did the authors use concentrations lower than 0.1%?

32. It's unclear if the odor-evoked responses were aligned to the inhalation. The authors should clarify and include additional newly aligned odor-responses (as latencies, and other metrics may shift the results).

33. Lines 787-788 - the authors should highlight how the AON-TCs fits well with prior literature

34. The discussion could benefit from a more detailed speculation on the role of these parallel processing streams in the behaving animal.

Version 1:

Reviewer comments:

Reviewer #1

(Remarks to the Author)
The authors addressed all of my concerns and I now consider this article ready for publication.

I am glad that the deep learning model-based classification has been removed. Such models tend to overclassify, but this applies to training data. They should not achieve above chance performance on held-out test data. If they do, this is likely an indication of cross talk between training and test data or some other failed assumption. According to my understanding, the present classifiers are not performing above chance levels so this is no longer a concern.

Reviewer #2

(Remarks to the Author)
The authors have addressed all of my prior concerns, and the manuscript is now suitable for publication.

Reviewer #4

(Remarks to the Author)
The authors have provided additional analyses of the population and lifetime sparseness which has improved both the interpretability of their manuscript and strengthened their claims that projection-targets reflect another parallel stream of information for the olfactory system. These data are an important contribution to the field, and the manuscript has been substantially improved during the revision process. However, the manuscript could be strengthened by some additional clarifications which should be straightforward to implement.

Major Comments
1. One of our initial concerns focused on the question of how the 30-50% of MTCs that collateralize to both AON and APC contribute to odor coding. The addition of the new supplemental figure (S2) helps to clarify this point by comparing odor tuning in dual-labeled vs single-labeled cells. It would be informative to also generate a similar plot showing the response properties of single-labeled vs dual-labeled cells separated by cell type (MCs vs TCs) and in response to the concentration series if this data is available. Finally, the authors should clarify which analyses do and do not contain dual-projecting MTCs.
2. As we noted in our initial review, differences in the tuning properties of MCs and TCs are central to explaining the differences in target-specific MTC activity, as this paper shows in later figures. The importance of this distinction argues for the introduction of these cell-type specific differences earlier in the paper. Since this information is critical to the paper's argument, and (if we understand correctly), available for all of the data present in Figures 1-4, we would argue (in agreement with reviewer 2) that the repeated presentation of grouped vs separated datasets reads as redundant, and that the paper would be made more clear by separating the data by cell type from the start.
3. We note the addition of lifetime and population sparseness, and mutual information metrics, which we agree have significantly improved the paper. However, we feel that additional value would be added by comparing the performance of population decoders trained to decode odor identity and concentration separately using these two cell populations (AON vs APC projecting). Since neurons in APC/AON receive convergent input from many MTCs, this population decoding could better approximate the information available to these downstream cortical circuits. It would be informative to compare the overall decoding performance as well as the distribution of decoding errors (e.g. bias towards high or low concentrations) between the two populations.
4. The authors indicate that they classify cells by lamination, cell body size, and other morphological features. Given the importance of this classification, representative images of the different cell types (mitral and tufted cells) and the features with which they classified them would be helpful.
5. The difference in image quality between mitral and tufted cells in figure 6a illustrates the potential confounds of comparing neural activity of mitral and tufted cells without normalization, given that the amplitudes may simply reflect imaging quality at different depths (see e.g. 6d and 6e, where MCs have lower response amplitudes than TCs regardless of projection target). The authors should acknowledge this caveat and describe how they account for the potential confound of imaging quality at different imaging depths when comparing MCs and TCs. We would further urge the authors to consider dF/F normalization - which is straightforward - or to otherwise demonstrate that the differences observed between MC/TC responses are not due solely to depth.

Minor Comments

1. We appreciate the addition of information to the legends (including # of cells per projection area, information about concentration in earlier figures) which we feel have improved the paper's clarity. Could the authors also please clarify which odorant(s) were used in the concentration series experiments?
2. The following sentence is unclear: “Cumulative probability and sparseness plots showed double-labeled cells located between AON or APC-projecting cells. Interestingly, significance levels increased for both lifetime (ANOVA, post hoc test, p<0.001) and population sparseness (ANOVA, post hoc test, p = 0.001), suggesting that, although not all OB output neurons project to a single cortical area, those that do show a highly significant difference in their coding properties.
3. The following sentence is unclear: “For both AON- and APC-traced OB output streams, we found that about a fourth of the identified cells were TCs and that on average more TCs showed at least one significant odor response than MCs for both traced populations”
4. It's unclear if figures 6-7 use the dual injection strategy or if it uses the approach in figure 1 (diff mice with the same calcium indicator). The author could clarify this in their figures by including a schematic and/or more detail in the figure legends.
5. Row 316 should say ‘sites’
6. This sentence in the abstract is unclear: However, differences in target outweigh pure cell-identity differences, as demonstrated by support vector machine classifier analysis. Please clarify.

Open Access This Peer Review File is licensed under a Creative Commons Attribution 4.0 International License, which permits use, sharing, adaptation, distribution and reproduction in any medium or format, as long as you give appropriate credit to the original author(s) and the source, provide a link to the Creative Commons license, and indicate if changes were made.
In cases where reviewers are anonymous, credit should be given to 'Anonymous Referee' and the source.
The images or other third party material in this Peer Review File are included in the article’s Creative Commons license, unless indicated otherwise in a credit line to the material. If material is not included in the article’s Creative Commons license and your intended use is not permitted by statutory regulation or exceeds the permitted use, you will need to obtain permission directly from the copyright holder.
To view a copy of this license, visit https://creativecommons.org/licenses/by/4.0/

Reviewer 1

	General issues for consideration:	
*** *1.***	**According to the tracing analysis, a large fraction of cells target both areas. In fact, I would expect this to be an underestimate of the extent of overlap. This weakens the argument of separation into different streams. More importantly, it could explain why the TCs – which show less overlap – have more distinct features for subpopulations projecting to each of the two regions. Please address this issue in the text.**	Thanks for raising this concern, which could be a potential drawback. We have addressed the fraction of cells that show collaterals in both areas and calculated it over four mice, which we expect to provide a fairly good representation of the total overlap. While half of the MC population showed double labeling, it was one-third of the population for TCs, which corresponds well with the literature (Chen et al. 2022). Indeed, this could partially explain the stronger disparity of properties in the TCs, and we have commented on this now in the discussion (line 751-758):<br>"A limited set of experiments involving virus injections into AON and APC using different-colored fluorophores allowed us to estimate the number of dually labeled neurons, with approximately one-third of TCs and roughly half of MCs exhibiting double labeling. This labeling likely results from a population of MCs and TCs branching in both the AON and the APC (Hirata and Fujisawa 1999) which is consistent with previous reports (Chen et al. 2022). Removing dually traced cells in the analysis of these experiments made distinctions between differentially traced populations clearer, supporting the concept of projection-dependent information routing."
***** *2.***	***As mentioned above, the differences between cells with distinct projection targets are not huge. One explanation may be that there are other, perhaps more dramatic, differences, which cannot be detected with the current analyses, and particularly, the rather small stimulus set used. Another possibility is that the differences are in realty rather subtle. I think that this point should be addressed.***	We thank the reviewer for suggesting a deeper analysis, as it made the manuscript significantly stronger.<br>To shed more light on differences in selectivity and sensitivity of differentially projecting cell populations, we added analyses of population and lifetime sparseness (Fig. 3F, 4H, 6G und 7F) as well as mutual information (Fig. 7E), which<br>1) confirmed the stronger odor selectivity of APC projecting cell types and that AON TCs are especially broadly tuned (selectivity) and<br>2) indicated that though APC projecting TCs show the strongest concentration-variant responses (the biggest response changes upon increase in concentration), AON projecting cells, especially AON MCs, might also transfer considerable concentration information. In these cells, this is probably not realized as a rate code (one cell/the population shows stronger responses with higher concentrations) but rather as a population code, i.e., different cells in the population respond to different

		concentrations. The sparse responses in AON-projecting cells (Fig. 7F) and their low mutual information across different odor concentrations (Fig. 7E) provide strong evidence for this mode of concentration coding.<br><br>Overall, these additional analyses support the first possibility, that previously more dramatic differences could not be detected due to inherent limitations of the analyses rather than an absence of distinct properties in differentially projecting neurons.
	General data analysis issues:	
1.	**Was there only one repeat per stimulus? This should be clarified.**	Yes, responses are from single presentations, as GCaMP6 responses to odorants were large and robust. We have stated this now in line 226-227:<br>"GCaMP6f responses to single presentations of each stimulus were imaged and measured."
**2.**	**The authors show that response selectivity cannot be easily accounted for by maximal signal strength. Isn't the more relevant metric "signal to noise"? With S/N I mean variability as evaluated without a stimulus, and the strength of the response. Using this measure, I would ask if there is correlation between S/N and selectivity.**	Thank you for your comments. This is a valid concern, as differences in signal-to-noise between populations could explain differences in the number of significantly responding cells, using a criterion of 2 SDs from baseline. To address this, we have<br>(1) analyzed a 4-second-long baseline before odor onset in all recorded neurons. Standard deviations of AON and APC traced neurons were not significantly different from each other ((23,2 (AON) vs. 24,3 (APC), p= 0.950 – Mann-Whitney U test). This information was now integrated into the methods line 228<br>"As standard deviations of AON and APC traced neurons were not significantly different from each other ((23,2 (AON) vs. 24,3 (APC), p= 0.950 – Mann-Whitney U test) responses were…."<br><br>(2) We have furthermore expanded the metrics of selectivity by calculating the lifetime sparseness of these populations. There, we show significant differences among traced populations: APC-projecting neurons exhibit higher lifetime sparseness, i.e., higher odor selectivity. The figure for this is now integrated into Fig. 3F.<br>We therefore think that differences in tuning are not due to differences in the signal-to-noise ratio among the populations.

3.	Other than controlling rate and depth of breathing, I don't see the benefit of the rather complex tracheotomy procedure. Could this form of (retro-nasal, if I understand correctly) breathing impact the sniff-locked responses? I feel that this point should be addressed/discussed.	Thanks for bringing this point up. The double tracheotomy procedure we used enables artificial sniffing, providing complete control over orthonasal odor stimulation. This method was described in detail in (Cheung et al. 2009), increasing the reproducibility of results, reducing noise, and overcoming optical signal issues caused by natural breathing. Thanks to these advantages, this procedure was employed in many other studies (Wachowiak and Cohen 2001; Spors et al. 2006; Carey and Wachowiak 2011; Wachowiak et al. 2013; Medinaceli Quintela et al. 2020; Boehm et al. 2020). Furthermore, a key advantage is the reproducibility of results across animals and odor presentations, as odor and inhalation onsets are precisely timed and inhalation duration is constant, so no "time warping" is necessary.<br>As in "artificial sniffing," the airflow directly mimics the natural inhalation, we are certain that sniff locking will not be affected, especially since our results in Fig. 8 on TC inhalation-triggered transients (Fig 8B) reflect previous findings on sniff coupling observed with GCaMP6f "Overall, baseline sniff-coupling is most clearly observed in TCs with imaging,…" (Ackels et al. 2020).
4.	Please clarify how Ca imaging data was up-sampled. What algorithm was used. ??	Thank you for pointing this out. This was incorrect. No up-sampling has been performed. This section is now removed.
5.	You write: "We employed a univariate feature ranking method for classification, utilizing chi-square tests to assess feature importance based on the characteristics of each feature, including variance and relevance to the response". What decisions were made based on this ranking? More generally, the description of the SVM analyses (and the point of Figure 8F) lack in detail and are not clear.	Thank you for your comments. We agree that the feature importance/SVM analysis needs further description. We used features (amplitude, rise time, latency, and fall) extracted separately for each sniff from fitted 2-photon calcium imaging traces (see methods, Fig. 8A) comprising 36 sniff-features (4 features x 9 sniffs). To measure sniff-feature importance, we employed feature selection algorithms. A filter-type feature selection algorithm (we used a univariate feature ranking for classification using chi-square) measures feature importance based on feature characteristics, such as variance and relevance to the response (available in the MATLAB Statistics and Machine Learning Toolbox). This filter-type feature selection is uncorrelated to the training algorithm. Our data pool contained 1431 cell odor pairs (AON mitral 468, AON tufted 287, APC mitral 512, APC tufted 164). Data was down-sampled to match the number of data points in the smallest category. Down-sampling was repeated 100.000 times (bootstrapping), each followed by feature ranking. The results are depicted in Figure

		8F, demonstrating that the feature “amplitude” had the highest feature importance, followed by “onset” and “risetime”. To provide a clearer explanation in our manuscript, we have integrated it into the methods line 283-299 and results line 708-728.
6.	***In figure 1D, I really cannot see the overlap and how the shaded regions match what is seen in the upper plots.***	We admit this has been insufficiently explained. Contour plots show consensus topographies, i.e., they reflect the most reliably activated areas across several animals, which only roughly overlap with the strongest responses in a single animal, as depicted in the upper three rows. Our tested odorants evoked responses with similar topography across the dorsal OB for all 3 conditions (AON-traced, APC-traced, PCdh21-GCaMP6f)), with peak responses in overlapping domains for each odorant.<br>We stated this now more clearly in the results<br>Line 389-397<br>“In order to explore potential differences in topographic response maps for different odors, consensus topographies (contour plots) (Bozza et al. 2004) were calculated and compared between the virally traced or transgenic mice. (Bozza et al. 2004). Each map was thresholded at 40% of the maximal amplitude, normalized, and responses to the same odorant were averaged across preparations to assess whether odorants evoked similar patterns across the dorsal surface of the OB. Contour plots show consensus topographies, i.e., they reflect the most reliably activated areas across several animals. Odorants evoked responses with similar topography across the dorsal OB for all three conditions (AON-, APC-virally traced, and transgenic), with peak responses in overlapping domains for each odorant.”
*7.	**It seems that the conclusion regarding different selectivity as a function of target region is based on visual inspection of Figure 3C. There are no statistics as far as I can judge. I would expect some more quantification, including a comparison of distributions of lifetime sparseness of neurons from different classes. Also, while cumulative frequencies are informative, they make it harder to grasp the general form of the distribution. Please include the non-**	Thanks for raising this concern. To address your question, we added the analysis of lifetime sparseness (Fig 3F), which showed significantly sparser responses for APC neurons (lifetime sparseness: $p = 0.002$, Mann-Whitney U Test) and thus higher selectivity. This result was even further supported when we proceeded to separate these streams into TCs and MCs with the same sparseness analysis (Fig.6G)(($p = 0.04$ Kruskal-Wallis test; Dunn’s Post Hoc Comparison, $p = 0.102$ AON-MCs vs. AON-TCs, $p = 0.064$ AON-MCs vs. APC-MCs, $p =0.049$ AON-MCs vs. APC-TCs, $p = 0.002$ AON-TCs vs. APC-MCs, $p = 0.003$ AON-TCs vs. APC-TCs, $p =0.556$ APC-MCs vs. APC-TCs). Here, it became clear that the lower selectivity of AON-traced cells primarily is derived from the low selectivity of AON-projecting TCs.

	**cumulative distributions (how many neurons responded to any number of stimuli) in this and in similar plots.**	For the non-cumulative distribution we have provided cell numbers at each odor rank in line 482-484.
8.	***The conclusion in the statement "AUC of rank-averaged odor response amplitude for APC- and AON-traced MTC was not statistically different for the two populations (Fig. 3D, AON-traced AUC 881,12 ± 615.93 (mean ± SD); APC-traced AUC 841.96 ± 736.58; p = 0.06 Mann-Whitney U Test), further demonstrating no major impact of signal amplitudes." Is not clear to me. As part of this, the phrase "AUC of rank-averaged odor response" is not self-explanatory and leaves the reader guessing. What exactly are the rank averaged odor responses?***	Thank you for pointing this out. This section was rewritten to clarify our analysis. The section that the reviewer mentions is now preceded by:<br>Line 485-487<br>"To analyze odorant response strength across cells, we ranked each unit's odorant responses by amplitude, from the largest (rank 1) to the smallest (rank 8). Odorant responses were then ordered by rank and averaged in a rank-specific manner."
9.	***Concerning the statement: "A similar fraction of AON- and APC-traced MTCs responded significantly to at least one odor concentration", is this for one specific odorant or across all odorants? I think that this distinction is significant, because the number of effective odors differs for the two areas, as shown here.***	This metric was taken across different odorants. Each randomly chosen FOV was tested with a set of 8 odorants. For selectivity calculations, cells were included that showed a significant response to at least one of the tested cells. Despite similar odorant sets of predominantly chemically related odorants, APC-traced cells showed significantly narrower tuning than AON-traced cells. To clarify this, we have included a section in the results that states this:<br>Line 509-511<br>"A similar fraction of AON- and APC-traced MTCs responded significantly to at least one odor concentration across all tested odorants"
****10.***	***Re the statement: "When calculated on a cell-concentration basis, AON traced MTCs exhibited a significantly lower fraction of significant responses compared to APC traced cells", Doesn't this argue for higher concentration selectivity for AON cells? If the fraction of responding cells across regions is similar, but there are more cell odor pairs for***	Thank you for your suggestion. Within the measured populations, both the average amplitude and the cell number increase more strongly with odor concentration for the APC; we interpret this as the APC having a higher capacity for concentration coding at least in terms of rate, or dose response coding. The larger sparseness seen for the concentration responses in AON projecting cells also hint to those cells coding for concentration but with each unit coding sparsely for a narrow concentration range it would have to occur via population coding. We have changed the Results and Discussion now to reflect this.

	APC, then this implies less concentration selectivity for the APC. No?	E.g. in Line 678-682:<br>"In conclusion, APC projecting cells exhibit the strongest capacity for concentration-dependent responses. This suggests that APC-projecting cells convey concentration-dependent information by increasing response strength, whereas AON-projecting MCs respond more sparsely to different odor concentrations, which might point to population-based concentration coding."
***<br>11.**	***A key observation (Figure 4), which is the basis for one of the main conclusions here is that: "APC-traced cells reacted far more strongly to an increase in odor concentrations". However, if you scale the two regions in Figure 4F to the same maximum value, is there still a difference in the form of the dependence? No doubt that there is a difference in maximum amplitude, but this may be just a difference in scale. Here too, I think that concentration dependence should be analyzed and compared more rigorously.***	Thank you for your comment. We have now included a figure showing the average amplitudes of projection-target-defined populations, normalized to the respective maximum values for each neuron (Fig. 4 G). Though the differences are less obvious, they are still significant.
***<br>12.**	**connected to the previous comment: The non-monotonic concentration response of AON cells is not discussed. This could certainly account for the contrast between the number of cells and the number of cell-concentration pairs. Right?**	This would have been indeed very interesting. We have investigated the monotonicity of AON and APC projecting units to assess how uniformly they increase over the applied concentration range and at what concentration they reach their maximum. Calculations were done similarly to the publication of (Leong et al. 2025). AON and APC projecting populations showed both a high number of non-monotonic units. We were, however, not able to show differences in the average monotonicity index (-2,3 vs -2,4), and both populations also had similar numbers of monotonic units (21,8 vs 20,9 %). Therefore, we do not think it can account for the discrepancies in the concentration responses of these units.

		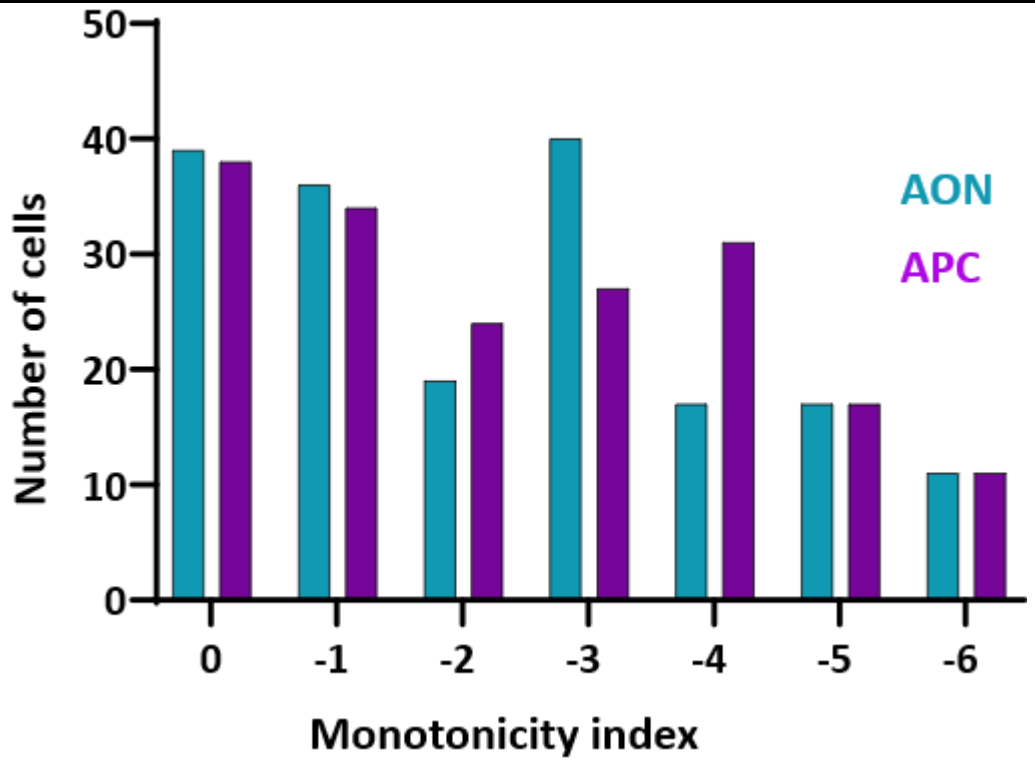
**13.**	**Classification analysis: "In comparison, a classifier trained on a dataset with randomized categories only predicted 495 samples correctly, achieving an overall accuracy of 35%, which demonstrates the information content contained in the true categories." Isn't chance performance 25%? Please clarify this point.**	Thank you for your comment. Overclassification is a common problem in classifiers like deep learning models. While it is correct that the change performance should be 25%, we did not simply assume this; rather, to control for overclassification, we calculated the "true change" performance of our model using downsampled data with randomized categories. A classifier trained on a dataset with randomized categories reached an overall accuracy of 35%. While some overclassification is present in our current model, the accuracy of the model trained on true categories is clearly higher (48.36%), demonstrating the information content contained in the true categories. Given the more informative nature of the SVM model, we agree with the reviewers that the additional benefit of providing the deep learning model (also given the overclassification problem) is limited and therefore removed it in the modified version of the manuscript.
***<br>14.**	**"APC-traced TCs reacted the strongest to an increase in odor concentrations". This is not at all apparent from Figure 7D. From that figure, all populations seem similarly concentration dependent.**	This is a fair concern, and we understand why Figure 7D might seem counterintuitive. However, we think that is a phrasing issue that creates a misunderstanding. All four populations show concentration sensitivity, as shown in Figure 7D, which compares responses to the lowest and highest odor concentrations (AON-MCs 96.18 ± 164.11 vs. 112.47 ± 97.17 ΔF (mean ± SD) $p=0.005$ Wilcoxon signed-rank; AON-TCs 128.77 ± 129.05 vs. 208.3 ± 124.96 ΔF (mean ± SD) $p=0.003$ Wilcoxon signed-rank; APC-MCs 112.83 ± 156.4 vs. 269.87 ± 259.24 ΔF $p<0.001$ Wilcoxon signed-rank; APC-TCs 103.1 ± 82.27 vs. 484 ± 562.56 ΔF $p<0.001$ Wilcoxon signed-rank)). This aligns well with observations from other studies that show concentration tuning in MTCs (Parabucki et al. 2019). However, when we compare the level of concentration sensitivity within each population using

		AUC analysis (Figure 7C) to identify potential differences between projection target-defined streams, APC-traced TCs showed the strongest amplitude change with increase in concentration (responses at 0.1% vs. 4% saturated vapor pressure; AON-MCs 96.18 ± 164.11 vs. 112.47 ± 97.17 ΔF (mean ± SD) p=0.005 Wilcoxon signed-rank; AON-TCs 128.77 ± 129.05 vs. 208.3 ± 124.96 ΔF (mean ± SD) p=0.003 Wilcoxon signed-rank; APC-MCs 112.83 ± 156.4 vs. 269.87 ± 259.24 ΔF p<0.001 Wilcoxon signed-rank; APC-TCs 103.1 ± 82.27 vs. 484 ± 562.56 ΔF p<0.001 Wilcoxon signed-rank).<br>Together with the results from the sparseness analysis, our conclusions on odor coding of the respective populations have changed as we write, e.g., here.<br>Line 678-682:<br>"In conclusion, APC projecting cells exhibit the strongest capacity for concentration-dependent responses. This suggests that APC-projecting cells convey concentration-dependent information by increasing response strength, whereas AON-projecting MCs respond more sparsely to different odor concentrations, which might point to population-based concentration coding."
	Presentation issues:	
* 1.	**Why do we need to go through the first part (without separation into cell types) before looking at separate populations? The first part seems somewhat redundant with the second, which is more informative. Why not start there?**	Thank you for highlighting this issue. We think it is important to first show the reader that our initial intention was not to separate these two cell types. We set out to examine projection-target-dependent differences in olfactory bulb output neurons. The separation of the two different types of output neurons was done only retrospectively, after we saw strong differences in an effort to explain these differences. Also, this categorization is not based on genetic markers but only on location morphology, which might be partially insufficient. Therefore, we think it is beneficial for the reader to see these parts separately.
**2.**	**Some of the labels and the colors make it quite hard to make quick sense of the data. It would help to have a specific color for each cell type, and a different line style for different regions. A case in point is Figure 6C, with confusing labelling. I suggest to add labels across the "columns" and maybe also right next**	Thanks for your suggestion. We have integrated this design now in Figures 6, 7, and 8 where we previously used 4 different colors. This suggestion improved data representation, as the graphs are now more intuitive to read. For the pie charts, the suggested changes were made in the figures, and you can see an example below:

	**to pie chart slices, to facilitate the interpretation of the charts.**	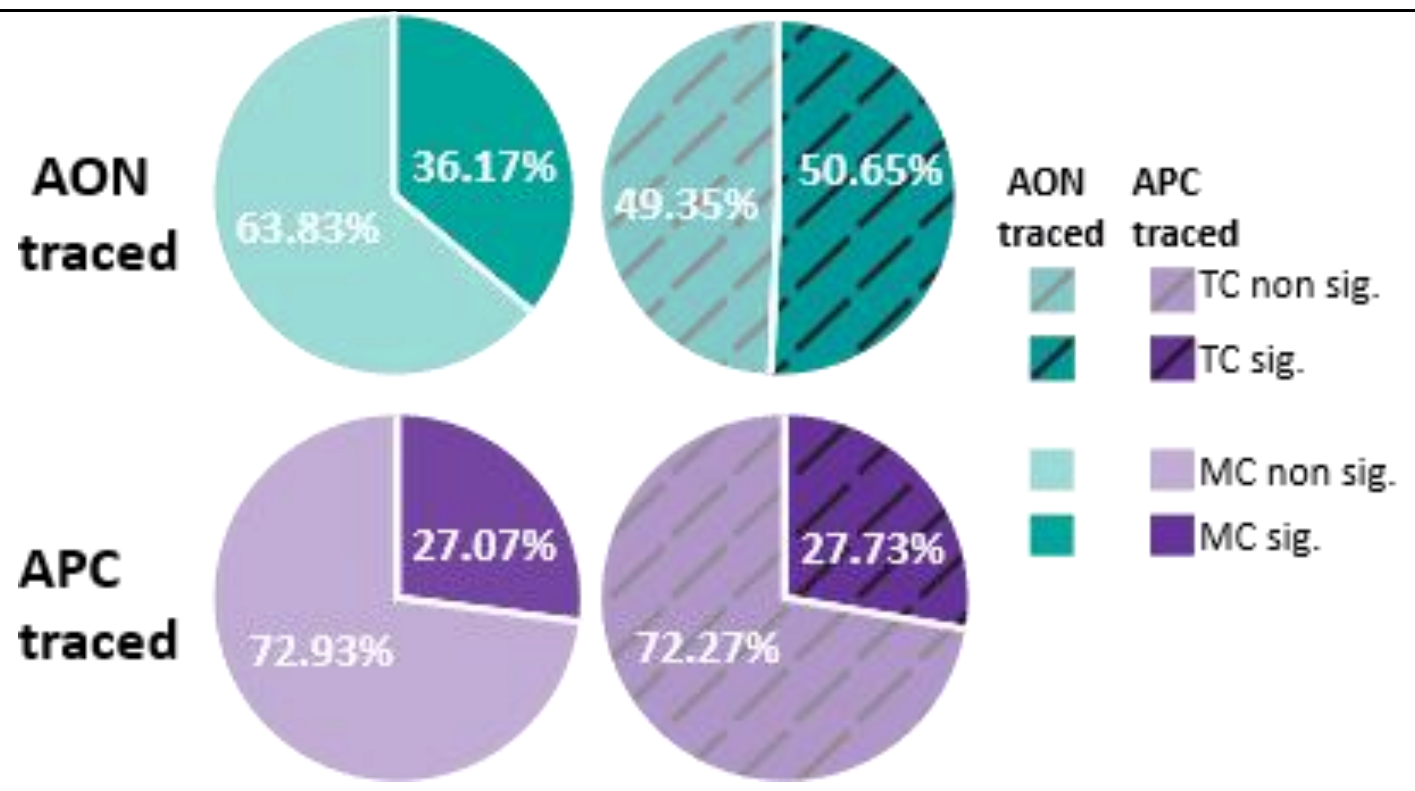
**3.**	**Although the differences between cell populations are not huge, it would be nice to try and link the observed differences to known function of the projection regions. If there is no obvious link this is ok too, but please at least address this issue.**	Thanks for bringing this up. While we understand why the differences might appear small, this is more a matter of the current tracing method and the limitations of the data analysis methods. To shed more light on differences in selectivity and sensitivity of differentially projecting cell populations, we added analyses of population and lifetime sparseness (Fig. 3F, 4H, 6G und 7F) as well as mutual information (Fig. 7E), which<br>1) confirmed the stronger odor selectivity of APC projecting cell types and that AON TCs are especially broadly tuned (selectivity) and<br>2) indicated that though APC projecting TCs show the strongest concentration-variant responses (the biggest response changes upon increase in concentration), AON projecting cells, especially AON MCs, might also transfer considerable concentration information. In these cells, this is probably not realized as a rate code (one cell/the population shows stronger responses with higher concentrations) but as a population code, i.e., different cells in the population respond to different concentrations. The sparse responses in AON-projecting cells (Fig. 7F) and their low mutual information across different odor concentrations (Fig. 7E) provide strong evidence for this mode of concentration coding.<br><br>It is hard to really match the observed differences to the functions of the two output regions, as the functions are not clearly defined. However, we now address this point in a separate part of the Discussion titled "*Differential output streams functions*"<br>Line 834-842<br>"One possible interpretation is that the AON, with its strong, fast, and sniff-modulated inputs, serves as a first responder in odor detection in animals where specificity is sacrificed for reliability and speed. The first sniffs of an incoming odor might then trigger responses in the animal, such as an increase in sniffing (i.e.,

		sampling rate), if warranted. This would also align with the findings of Schiltz et al., who report that AON neurons exhibit poor odor specificity but respond strongly to novel stimuli (Schiltz et al. 2025), and with the hypothesis that the AON might function as a tool for olfactory navigation (Brunert et al. 2023). The APC that receives more specific odor-related information would then be responsible for fine discrimination and valence coding, tasks that require such information.“
**4.**	**A reference or more detail about the deep learning model is needed.**	Given the more informative nature of the SVM model, we agree with the reviewers that the additional benefit of providing the deep learning model (also given the overclassification problem) is limited and therefore removed it in the modified version of the manuscript.
**5.**	**When discussing classification results, please define precision and specificity**	Machine learning algorithms can be evaluated using several metrics derived from the confusion matrix. The specificity, or true negative rate (TN / (FP + TN)) is the proportion of correctly classified negative instances out of all actual negative instances. Precision (Accurate Positives = TP / (TP + FP) assesses the proportion of correctly classified positive instances out of all predicted positives.<br>As the deep learning algorithm was criticized by the reviewers, we opted to exclude it from the manuscript.
**6.**	**I don’t understand the “(as might be expected)” comment on line 591. If it is obvious, then why say it. If it is not obvious, then explain why this is expected.**	Thanks for highlighting it. This comment is now removed.
**7.**	**I cannot follow the logic of the discussion in lines 809-814. The argument about specificity seems quite vague. Please clarify.**	We concede to this point and have expanded the discussion on this point under “Possible impact on corticofugal projections”. This section now reads (line 845 - 866)<br>“The OB output streams investigated in our study not only carry olfactory information to different cortical areas but also receive feedback modulation from both AON and APC. Top-down projections from AON to the OB are more odorant-specific than those of APC (Chae et al. 2022; Amrutkar et al. 2025; Rothermel and Wachowiak 2014; Otazu et al. 2015; Banerjee et al. 2015). But how does the AON support stimulus-specific feature projections to the OB if it receives broadly tuned input?<br><br>Broad, unspecific input to the AON does not imply that the backprojections to the OB must be equally unspecific. AON-

		derived projection fibers likely project to similar glomerular columns from which they receive information, as has been shown for bilateral connections (Grobman et al. 2018)1 and odorant-activated signals in AON fibers on the level of the OB are also centered around those OB areas that are naturally activated by this respective odor (Rothermel and Wachowiak 2014).<br><br>Although we cannot rule out that differential odor processing is influenced by backpropagation, we think it might play only a minor role in this experimental setup, and that the effects shown here are due to intrinsic cellular heterogeneity. The reason is that both AON- and APC-derived cortical fibers are glutamatergic but exert a predominantly inhibitory effect on the OB network (Medinaceli Quintela et al. 2020; Otazu et al. 2015). This requires activation of the OB's inhibitory network, which exhibits markedly reduced excitability in the anesthetized animal (Wachowiak et al. 2013).<br><br>In the awake animal, however, AON-derived activity, backcoupled precisely to previously activated areas, is therefore potentially responsible for dampening ambient odors to increase signal-to-noise or improve gating of behaviorally relevant odorants. The AON's speed and reliability advantage might allow modulation to occur well before processing in the APC can begin. Simultaneous recordings of bottom-up and top-down information might shed light on the respective timing of these pathways."<br><br>Together with the response to question 3, we hope that this argument is now clearer to the reader.
**8.**	**In Figure 4A, please indicate which cells correspond to these traces? (that is, projection region and subtype)**	We have added the projection region information for the respective traces into the figures and legends. As there is no subtype separation in Fig 4, we have not integrated subtype information into the figure. However, in Figure 4A, cell 1 is a TC, while the other three cells are MCs.
9.	"**concentration-averaged odor response amplitude for both APC- and AON-traced MTCs significantly increased with odor concentration". I expected that concentration averaged means an average over concentrations. I believe this is just**	We are sorry for the confusing phrase. This actually refers to the average response across units at this particular concentration. To clear it up, we have rephrased the sentence:<br>Line 522-523:<br>"Whereas the averaged odor response amplitude for both APC- and AON-traced MTCs significantly increased with odor concentration… ".

	**an issue phrasing, but please clarify.**	
	Minor:	
**1.**	**In "(see chapter "Sniff modulation analysis") – change chapter to section**	This has been changed.
**2.**	**The sentence beginning on line 388 ("To display topographic …") is a bit hard to read. Consider rephrasing.**	Thanks for bringing this out to our attention. To make it easier to read, this sentence has been rephrased:<br>Line 389-397:<br>"In order to explore potential differences in topographic response maps for different odors, consensus topographies (contour plots) (Bozza et al. 2004) were calculated and compared between the virally traced or transgenic mice. (Bozza et al. 2004). Each map was thresholded at 40% of the maximal amplitude, normalized, and responses to the same odorant were averaged across preparations to assess whether odorants evoked similar patterns across the dorsal surface of the OB. Contour plots show consensus topographies, i.e., they reflect the most reliably activated areas across several animals. Odorants evoked responses with similar topography across the dorsal OB for all three conditions (AON-, APC-virally traced, and transgenic), with peak responses in overlapping domains for each odorant."

Reviewer #2 (Remarks to the Author):

	Major concerns:	
1.	As the authors used the retrograde labelling and the target areas are nearby, it's important to show the evidence on the specificity of injection sites. Please show some examples proving the specificity of injections. This is critical for dual tracing experiments.	We understand the reviewers' valid concern regarding the specificity of the viral expression and the overlap between AON and APC projecting cells due to collaterals. Calculations of dually infected cells correspond well with the numbers of output neurons with collaterals (Chen et al. 2022), including a lower number of TCs showing dual infection. We therefore assume that the majority of dually traced cells arise from collaterals. To further convince the reviewers of the specificity of our approach, we added exemplary images of injection sites and a schematic figure depicting all reconstructed injection sites (Supp. Fig 2). Furthermore, the coordinates are the same as those used in previously published, successful tracing experiments (Medinaceli Quintela et al. 2020; Rothermel and Wachowiak 2014;

		Wachowiak et al. 2013). About 0.5 mm separates AON and APC in our coordinates, the same distance used to selectively pharmacologically inhibit either AON or APC (Chae et al. 2022).
2.	**In lines 240-245, the authors explain the selection criteria. Was this applied in all experiments? Please justify this in the context of narrow vs. broader tuning properties**.	Yes, responses were considered significant when they exceeded 2 times the baseline standard deviation. As the standard deviation between AON and APC projecting cells was not significantly different (23,2 (AON) vs. 24,3 (APC), p= 0.950 – Mann-Whitney U test) (line 228-229) we consider this method for determining the number of significant responses, and thus the tuning properties of a neuron, appropriate.
3.	**Did authors observe external tufted cells projecting to the APC/AON? Are there any observations of synapse-crossing with the viral vector that the authors have used?**	We did not specifically look for external tufted cells, because they do not project outside the OB. As we performed retrograde labeling with injections into AON and/or APC, we did not expect to find this cell type in our data set as they do not express Cre-recombinase. This retrograde labeling of MCs and TCs also did not show any synaptic transfer, neither in the anterograde direction (there were no cells visible in the cortices) nor in the posterior direction (labeled interneurons inside the bulb), as these postsynaptic cells are also not expressing Cre-recombinase necessary for viral construct expression. These tracing results match well with previous data, where no synaptic transfer was observed (Rothermel et al. 2013).
4.	**Figure 1D analysis: "more noticeably diffuse component" – Do authors expect any artifacts due to viral transduction in the experimental groups? Please increase the 'n' of control group to 5-6.**	We understand why this phrase might give the impression of artifacts in viral transduction. However, the more diffuse component arises due to an inherent property of PCdh21 expression. As previously shown by (Wachowiak et al. 2013), contour plots derived from widefield imaging of PCdh21-GCaMP display a more diffuse signal than those from other models in which GCaMP was expressed in ORNs or TH-positive neurons. As they have confirmed with additional two-photon imaging, this effect arises from the combination of widefield imaging and labeling of exclusively MTCs, which are known to form a widespread network of primary and secondary dendrites. Therefore, PCdh21 expression appears more diffuse in widefield imaging, even though a discrete glomerular signal can be detected with 2-photon imaging. A critical point is that PCdh21 is expressed in both MTC dendrites and ORN axon terminals. In our experiments, we employed a retrograde viral labeling approach in PCdh21-Cre mice, resulting in GCaMP expression only in MTCs, unlike the PCdh21-GCaMP transgenic line. The difference in

		diffuse components between these groups arises from differences in the cell population being labeled.<br><br>In order to make this clearer, we have now added the following to our manuscript:<br>Line 401-404:<br>“Compared to PCdh21-GCaMP6f mice, maps of AON- or APC-traced MTCs displayed a more noticeably diffuse component, as was expected from previous publications (Wachowiak et al. 2013) (Fig. 1D, middle and lower rows), which has been speculated to be caused by the exclusive expression of GCaMP in mitral and tufted cells with their wide dendritic arborization.”
**5.**	**Lines 441 - 442: “Significant inhibitory responses were observed only in 2.5% of AON-traced neurons and none in APC-traced neurons. Due to the low number of inhibitory responses, further analysis was conducted only on excitatory cell-odor pairs (COPs)”. How did authors classify inhibitory responses? Please explain how the authors delineated excitatory and inhibitory responses. Please discuss the inhibitory action.**	Inhibitory responses were considered significant if ΔF values diverged from the baseline more than 2x SD of the pre-odor baseline (in the negative direction). We were also surprised not to observe more inhibitory responses. However, as can be seen from previous papers (Economo et al. 2016; Congiu et al. 2023; Vanwalleghem et al. 2020) inhibitory responses are much smaller than excitatory ones, and we might not have picked them up with our metrics. Therefore, we decided to report exclusively on the increase in cellular activity upon odorant stimulation, but cannot exclude that other distinctions between traced populations might be found in inhibitory responses. We also added a short discussion to the text at line 446
**6.**	**Line nos. 380 and 401: Why are the animal numbers different for the AON group?**	Please excuse us for this error. The numbers have been corrected in the text (5 animals for APC, 6 animals for AON).<br>Line 380 and 408
**7.**	**Line no. 330 says “Only mice showing correct AON or APC targeting were used for analysis.” – Please explain the exclusion criteria.**	All mice that underwent imaging were perfused after the experiment, and histological sections were collected to confirm the correct injection target. This was confirmed by reconstructing the needle tracts (as there are no Pdh21-expressing cells within AON or APC that could provide orientation). The animals showing incorrect targeting were excluded from the analysis. We have now created a supplementary figure showing the end of these needle tracts and examples of histological sections from AON- and APC-injected animals.
**8.**	**Authors declare that AON-traced cells were predominantly located in**	Thank you for your comment; however, we believe this may be a misunderstanding. We have now made it clearer in the discussion

	**the external plexiform layer whereas APC-traced cells are primarily found in the mitral cell layer. Please discuss the observed differences in odor selectivity in detail in the context of differences in their arborization, connectivity with neighbouring neurons, channel density, and the inhibitory feedback (Burton and Urban 2014, Fukunaga 2012).**	that we have separated the dataset by projection target and cell type. Both projection-defined populations contain roughly equal numbers of mitral and tufted cells, so differences in TCs and MCs cannot account for the differences we see between AON- and APC-projecting populations. We have integrated the referenced studies into the discussion of the respective cell types, supporting our hypothesis about the function of TCs.<br>Line 817 – 822<br>“TCs have furthermore been shown to form a more heterogeneous cell population (Nagayama et al. 2014), receive stronger afferent input than mitral cells (Gire et al. 2012), exhibit greater excitability and more irregular firing patterns (Burton and Urban 2014), and respond with more stable timing relative to sniff onset (Fukunaga et al. 2012). All these findings support the idea that TCs are responsible for the rapid distribution of information to olfactory cortical centers”
**9.**	**Please elaborate on the discussion of correlation between breathing dynamics and the OB output, considering the recent reports of multimodality in the olfactory system (Salameh et al., 2024 Science; Mahajan et al., 2024 bioRxiv)**	We have done so in line 824-826<br>“Our data furthermore show that TCs have more detectable sniff-triggered transients, suggesting that they might also convey mechanical signals that OB output neurons encode, stemming from blood pressure pulsations (Jammal Salameh et al. 2024) and airflow through the nose (Mahajan et al. 2025).“
	Minor points:	
**1.**	**The age group of experimental mice is very variable (4 weeks to 1 year) – Please explain**	Please excuse this error; this is supposed to read 4 months to 1 year, and means that we used young adult to middle-aged mice. Ages did not differ between animals with AON- and APC-projecting cells labeled (7 +/- 2.9 months for AON and 8.4 +/- 2.7 months for APC; p=0.35, t-test).
2**.**	**It would be great to add the PID traces, as the authors used varying concentrations ranging from 0.1% to 4% (s.v.).**	We are sorry, as we did not record PID traces on a regular bases therefore we cannot provide them. However, our olfactometer design recapitulates the classical design used in Matt Wachowiak's group, which has been used in multiple previous publications e.g. (Wachowiak et al. 2013; Bozza et al. 2004; Verhagen et al. 2007).

**3.**	**Line 383: Presynaptic OSN expression of GCaMP6f – please clarify**	Pcdh21-cre mice are known to express Cre-recombinase also in olfactory receptor neurons as previously stated in (Wachowiak et al. 2013).<br><br>“In PCdh21-Cre mice, reporter-driven expression […] led to expression in MT cells and clear expression in ORNs (Fig. 1C).”<br><br>This led us to establish this retrograde tracing approach in the first place.

Reviewer #4

	Major Comments/Concerns	
**1.**	**The introduction frames the role of cortical feedback versus feedforward segregation as a dichotomy, and argues that the evidence presented in this paper favors a model in which OB outputs constitute independent information streams defined by cell type and projection target. However, this largely ignores the contribution of cortical feedback in differentially regulating the activity of projection cell types. In fact, studies (e.g. Chae et al. 2023) have shown that feedback from APC and AON selectively restructures the responses of its dominant projection type (TCs for AOn, MCs for APC). Without directly recording the activity of these corticofugal projections or employing manipulations to disrupt these projections, it is difficult to assess the degree to which the distinct properties of APC vs AON projecting MTCs are shaped by the functional properties of the MTCs themselves vs differential contributions of feedback projections from the different**	Thank you for your comment. We have expanded on this topic now in line 851-866 of the discussion, :<br><br>“AON-derived projection fibers likely project to similar glomerular columns from which they receive information, as has been shown for bilateral connections (Grobman et al. 2018) and odorant-activated signals in AON fibers on the level of the OB are also centered around those OB areas that are naturally activated by this respective odor (Rothermel and Wachowiak 2014).<br>Although we cannot rule out that differential odor processing is influenced by backpropagation, we think it might play only a minor role in this experimental setup, and that the effects shown here are due to intrinsic cellular heterogeneity. The reason is that both AON- and APC-derived cortical fibers are glutamatergic but exert a predominantly inhibitory effect on the OB network (Medinaceli Quintela et al. 2020; Otazu et al. 2015). This requires activation of the OB's inhibitory network, which exhibits markedly reduced excitability in the anesthetized animal (Wachowiak et al. 2013).<br>In the awake animal, however, AON-derived activity, backcoupled precisely to previously activated areas, is therefore potentially responsible for dampening ambient odors to increase signal-to-noise or improve gating of behaviorally relevant odorants. The AON's potential speed and reliability advantages might allow modulation to occur well before processing in the APC begins. Simultaneous recordings of bottom-up and top-down information might shed light on the respective timing of these pathways. “

	**cortical targets. The authors should discuss what is known about AON/APC feedback on MTC activity under anesthesia, whether feedforward and feedback streams maintain parallel identities, and how feedback could influence the tuning differences reported here.**	
**2.**	**The authors introduce this study under the premise that parallel processing streams of sensory stimuli leads to faster processing. However, the authors do not provide evidence nor discussion about whether their findings would support faster processing or the behavioral consequences of such coding mechanisms. This study could benefit from additional comments on this issue.**	We have added this now in line 834-842 of the discussion, speculating on a possible function of these output streams:<br><br>"One possible interpretation is that the AON, with its strong, fast, and sniff-modulated inputs, serves as a first responder in odor detection in animals where specificity is sacrificed for reliability and speed. The first sniffs of an incoming odor might then trigger responses in the animal, such as an increase in sniffing (i.e., sampling rate), if warranted. This would also align with the findings of Schiltz et al., who report that AON neurons exhibit poor odor specificity but respond strongly to novel stimuli (Schiltz et al. 2025), and with the hypothesis that the AON might function as a tool for olfactory navigation (Brunert et al. 2023). The APC that receives more specific odor-related information would then be responsible for fine discrimination and valence coding, tasks that require such information. "
**3.**	**Dual-color tracing (Fig. 5B–C) reveals that 30–50% of MTCs collateralize to both AON and APC. However, it remains unclear how these hybrid cells contribute to identity and concentration coding. For example, it would be interesting to note whether decoder performances change when excluding dual-projectors. A deeper analysis of their functional identity—e.g. whether they resemble mitral- or tufted-type kinetics—and how these properties align with the projection-specific parallel processing streams that the authors describe, would be helpful. Furthermore, the authors should clarify if this dual-projecting**	Dual-projecting population are included in both in the APC-projecting and AON-projecting analyses of MCs and TCs.<br><br>In response to the reviewers' comments we removed the deep learning model, and created a supplemental figure (Suppl. Fig. 2) where we show the cumulative probability as well as population and lifetime sparseness for the selectivity of exclusively AON -projecting, exclusively APC-projecting as well as dually infected cells. Our analyses show that yellow cells do not behave like AON- or APC-projecting cells, but their values fall between the two cell types. Even though our dataset was limited, sparseness analysis clearly revealed more distinct properties when double-projection cells were excluded. Overall, these additional analyses support the first possibility, that more dramatic differences could not be detected due to inherent limitations of the current analyses and stimulus set, rather than an absence of distinct properties in

	**population was included both in the APC-projecting and AON-projecting analyses of MCs and TCs.**	differentially projecting neurons. To address this, we have now included the following in our manuscript:<br>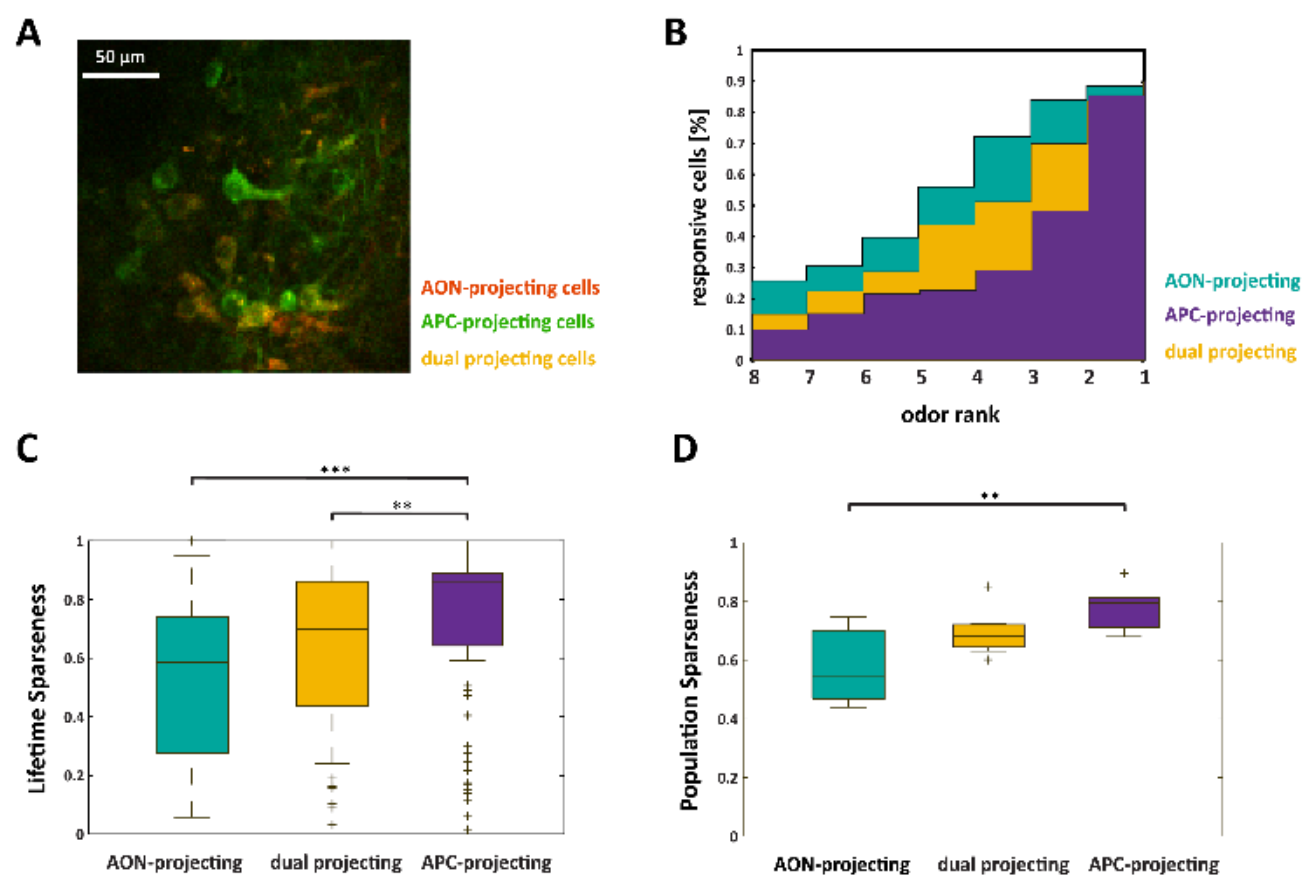<br><br>Suppl. Fig 2<br><br>Line 570-579<br>"In preparations that were simultaneously traced, we separated double-labeled cells from single-traced cells and analyzed their respective GCaMP6f-signals upon odorant stimulation from 2P imaging. From those cell populations (AON-traced, APC-traced, and yellow (double-labeled) cells), we plotted the cumulative probability of responding cells and calculated lifetime and population sparseness (Supplement Fig. 2) to compare them to our previous findings. Cumulative probability and sparseness plots showed double-labeled cells located between AON or APC-projecting cells. Interestingly, significance levels increased for both lifetime (ANOVA, post hoc test, $p<0.001$) and population sparseness (ANOVA, post hoc test, $p = 0.001$), suggesting that, although not all OB output neurons project to a single cortical area, those that do show a highly significant difference in their coding properties."
**4.**	**The pooled analyses in Figures 1–4 compare AON- versus APC-projecting populations that differ substantially in their MC/TC ratio (as confirmed in Figure 5C). Since it is well-established that MCs and TCs have distinct tuning properties, these differences may primarily reflect cell-type composition rather than**	We are sorry for this misunderstanding. In fact, both AON and APC projecting populations in Figure 1-4 have similar numbers of TCs and MCs. Imaging experiments were performed without this separation, and cell types were subsequently separated by depth and morphology by a researcher blind to the projection target. As a result, we found 57 TCs and 160 MCs in the AON-projecting cell population, and 64 TCs and 175 MCs in the APC-projecting cell population. These relatively equal cell compositions across the respective datasets might not reflect

	**target-specific coding. Given the centrality of this cell type distinction, it should be introduced more explicitly and earlier in the text. Additionally, the magnitude of this disparity calls into question the value of the analyses in Figures 1-4, which fail to control for projection cell type identity. The authors should attempt to quantify how much of the observed AON/APC differences can be explained by relative cell-type fractions, and discuss the implications for their pooled analyses.**	actual cell numbers, but they suggest that differences between populations cannot be attributed to differences in composition. It is also interesting to note that the lower selectivity of the AON is primarily attributable to TC properties, but TCs are not the majority in this population of AON-projecting cells.
	Minor Comments/Concerns	
1.	**A significant fraction of the analyses in the paper rely on computing the fraction of units which are significantly odor responsive. The methods define "significantly odor responsive" as exceeding two standard deviations of a pre-odor baseline, but do not specify the exact baseline window or the statistical criteria used to establish significance for cell-odor pair analyses. The authors should also assess how the population of responsive cells identified with criterion align with other standard measures of responsivity (e.g. auROC, Wilcoxon-rank sum analysis).**	Trials were usually 10 seconds long with a 4 s pro-odor phase. Baselines were calculated for each trial as the mean and SD over the 4 seconds preceding odorant stimulation. Responses (positive or negative) were considered statistically significant when they exceeded 2 x SD of the baseline.
**2.**	**In Figure 1D, the authors note that viral GCaMP expression in target-defined output neurons yields a significantly higher diffuse signal in the OB than transgenic Pcdh21-GCaMP approaches. The authors should comment on how their**	Thanks for raising this point, which Reviewer 2 also mentioned. As previously shown by (Wachowiak et al. 2013), contour plots derived from widefield imaging of PCdh21-GCaMP display a more diffuse signal than other models in which GCaMP was expressed only in ORNs or TH-positive neurons. As they have confirmed with additional two-photon imaging, this effect arises from the combination of widefield imaging and exclusive

	**retrograde viral strategy affects MTC coverage relative to other work.**	labeling of MTCs, which form a spread network of primary and secondary dendrites. Therefore, PCdh21 expression appears more diffuse in widefield imaging, even though a discrete glomerular signal can be detected with 2-photon imaging. A critical point here is that the PCdh21-GCaMP line expresses GCaMP in the dendrites of MTCs as well as in the axon terminals of ORNs. In our experiments, we have employed a retrograde viral labeling approach in PCdh21-Cre mice, which results in GCaMP expression only in MTCs, unlike the PCdh21-GCaMP transgenic line. As shown previously, we argue that the difference in the diffuse component between these groups stems from differences in cellular expression.<br><br>To make our analysis clearer, we have now rephrased the corresponding sentence to:<br>Line 391 - 398 :<br>“Each map was thresholded at 40% of the maximal amplitude, normalized, and responses to the same odorant were averaged across preparations to assess whether odorants evoked similar patterns across the dorsal surface of the OB. Contour plots show consensus topographies, i.e., they reflect the most reliably activated areas across several animals. Odorants evoked responses with similar topography across the dorsal OB for all three conditions (AON-, APC-virally traced, and transgenic), with peak responses in overlapping domains for each odorant. Activated domains highly overlapped for each odorant (Fig. 1D, lower panel, contour plots).”
**3.**	**It’s unclear why the MTCs in figure 2B are grouped together, rather than separated, since the cells would’ve been imaged across different imaging planes. For clarity and ease of comparison in future figures, the authors should indicate which cell-odor pairs are MCs vs TCs. Similarly, it’s unclear if the data in figure 6B is the sorted version of the data presented here. The authors should clarify.**	Thank you for bringing this to our attention. Within the scope of this manuscript, we first examined projection-target-dependent differences in olfactory bulb output neurons. We collected data for MCs and TCs across different imaging planes. The separation of the two different types of output neurons was done only retrospectively, after we saw strong differences in an effort to explain these differences. As a result, this categorization is not based on genetic markers but only on location morphology, which might be partially insufficient. Therefore, we thought it would be beneficial to first introduce the overall differences between MTCs projecting to different regions in the first part of the manuscript, and then present the further subdivided population differences.<br><br>To make it clear that the data in Figure 6B is the sorted version of the data in Figure 2B, we start the section “AON-targeting TCs

		display high odor responsiveness but low odor selectivity" with the following explanation:<br><br>Line 582-584:<br>"We separated our functional 2-photon microscopy dataset, obtained with the initial approach (Fig. 1A), into MCs and TCs."
**4.**	**Given the A-P axis differences observed in figure 1, the authors should clarify relatively where the 2P imaging took place.**	FOVs for imaging were chosen based on activity hotspots. Unfortunately, we cannot reconstruct the exact location of FOVs.
**5.**	**In each figure legend, the authors should include the total numbers of cells per projection area included in the analyses to supplement the fractional metrics.**	Thanks for bringing this to our attention. We have added the numbers to the respective figure legends.
**6.**	**The authors should provide examples of the calcium traces returning to baseline in figure 2 so that the reader can get a better sense of the odor-response dynamics.**	We are really sorry that we cannot provide this. The majority of recordings lasted 10 seconds, with a 4-sec pre-odor phase, a 4-sec stimulation phase, and a 2-sec post-odor phase. This post-odor phase is, unfortunately, too short for most cells to return to baseline calcium levels. Both AON and APC-traced cells contain at least one cell that at least approaches the pre-odor baseline (cell 4 for AON and cell 3 for APC).
7.	**To supplement the cell-odor pair analysis in figure 2b, the authors should include a cell x odor-evoked responses for all odors so that the reader can visualize the response sparseness and variability for different populations more clearly.**	We assume that this comment refers to visualizing odorant-specific responses. As we imaged at different FOV on the dorsal surface, the maximal amplitudes of cells to particular odorants by themselves are, in our opinion not really meaningful. We have, however, now included analyses of lifetime and population sparseness in the selectivity section of the manuscript, so we hope this satisfies the reviewer.
**8.**	**A comment about the breathing trace is needed in figure 2 legend.**	Thanks for pointing this out. The following was added to the figure legend of Fig. 2F<br>Line 909-911:<br>*"Sniff-aligned average mean fluorescence trace of significant odor-responsive AON- (696 COPs) and APC-traced (507 COPs) MTCs. Grey bar marks the odor stimulation."*

<table>
<tr><td>9.</td><td>For all figures which include mean calculations, the authors should indicate how many cells were included (e.g. Figure 2F, 3E,7D etc.) and what the population sparseness is for both odor concentrations and odor identities. Alternatively, the authors could normalize by the minimum number of cells per group.</td><td>Thanks for raising this concern. Sparseness analyses were also requested by our other reviewers. We have provided the requested numbers in the figure legends (see question nr 5) and have added lifetime and population sparseness plots to Figures 3F, 4H, 6G, and 7F. This as well as mutual information (Fig. 7E),

1) confirmed the stronger odor selectivity of APC projecting cell types and that AON TCs are especially broadly tuned (selectivity) and
2) indicated that though APC projecting TCs show the strongest concentration-variant responses (the biggest response changes upon increase in concentration), AON projecting cells, especially AON MCs, might also transfer considerable concentration information. In these cells, this is probably not realized as a rate code (one cell/the population shows stronger responses with higher concentrations) but as a population code, i.e., different cells in the population respond to different concentrations. The sparse responses in AON projecting cells (Fig. 7F) as well as their low amount of mutual information when comparing responses to differing odor concentrations (Fig. 7E) provide strong evidence for this mode of concentration coding for the AON projecting cells.</td></tr>
<tr><td>10.</td><td>This study and others (including Zhang et al., 2025), have demonstrated variability in mitral and tufted cell responses, even for those that innervate the same glomerulus. Given the variability of cell responses, the authors should identify correlations between odor response vectors for all cells (sorted by cell type and projection region) so that the similarities and differences between cell class and projection region area apparent at a single cell level.</td><td>We constructed odor response vectors for all units and calculated the correlations between these vectors within and between the respective groups. Correlation was consistently low and not significantly different between within- and between-group values.

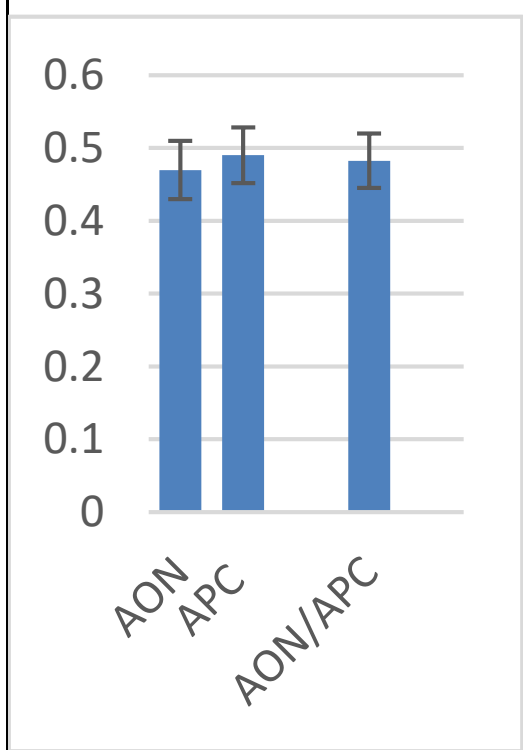

We think this is because FOVs were chosen randomly across the dorsal surface of the OB, making odor response vectors too variable.</td></tr>
<tr><td>11.</td><td>Line 451 – what was the concentration used in these experiments?</td><td>Except in experiments investigating concentration sensitivity, all odor presentations were conducted with 1% VP diluted in clean air. This information was integrated into the manuscript as the following sentence:
Line 442-444:</td></tr>
</table>

		"Figure 2B depicts odorant-evoked responses recorded from AON- (left) and APC-traced cells (right) in response to a panel of eight monomolecular odors at 1%VP (see Materials and Methods) sorted by odor-evoked signal strength."
**12.**	**It's unclear what the authors mean by lines 462-463. Given the known differences in mitral and tufted cell properties, comparing mean AUC for the combined population doesn't make sense – examples where the molecular identity slightly differs. The authors should clarify or delete this conclusion.**	Thank you for raising this concern. This study aimed to compare projection-dependent vs. cell-type differences. Therefore, we would argue that despite knowing the differences between mitral and tufted cell properties, comparing the mean AUC across the combined population is justified. Moreover, comparing AUC across the combined population suggests similar virus expression levels in those projection target-defined populations. We think this is important to be able to compare the populations. We also added information on the composition of our target-defined populations, which contained roughly the same number of MCs and TCs, as this information was not stated clearly enough in the original version of the manuscript.
**13.**	**The metrics that the authors use to quantify the breadth of odor tuning in Figure 3 (fraction of MTCs responding to N odors) are difficult to interpret. Given that the authors report a greater overall fraction of odor-responsive AON-projecting cells than APC-projecting cells, metrics that do not rely directly on the fraction of responsive cells will be informative. The authors should supplement this analysis with additional metrics of odor selectivity (such as lifetime sparseness, response entropy, selectivity index, ROC-based measures), and/or odor identity decoding analyses.**	Thank you for this suggestion, which clearly improved our data presentation. To address your question, we added an analysis of lifetime sparseness (Fig. 3F), which showed significantly sparser responses in APC neurons. This result was further supported by separating streams into TCs and MCs and performing the same sparseness analysis (Fig. 6G, left). We stated these in the following lines:<br>Line 498-503<br>"We calculated both lifetime sparseness (Fig. 3F, left) as a measure of single cell selectivity as well as population sparseness (Fig. 3F, right) as an indicator of how sparse the populations was reacting to individual stimuli. Both lifetime and population sparseness showed a statistical difference between projection-defined populations (lifetime sparseness: $p = 0.002$, Mann-Whitney U Test, and population sparseness: $p = 0.015$, Mann-Whitney U Test) with APC-traced MTCs showing a higher selectivity than AON-traced APCs."<br>And line 622 – 626<br>"To quantify differences in selectivity between the cell types we calculated lifetime sparseness (Fig. 6G, left) and were able to show that AON projecting MCs and TCs exhibited lower sparseness than APC projecting cell types (($p = 0.04$ Kruskal-Wallis test; Dunn's Post Hoc Comparison, $p = 0.102$ AON-MCs vs. AON-TCs, $p = 0.064$ AON-MCs vs. APC-MCs, $p =0.049$ AON-MCs vs. APC-TCs, $p = 0.002$ AON-TCs vs. APC-MCs, p =

		0.003 AON-TCs vs. APC-TCs, p =0.556 APC-MCs vs. APC-TCs)."
14.	The visualization of odor responsiveness in figure 3B is difficult to interpret. In order to understand how diverse the responses are across odors, the authors should include a figure that illustrates maximum dF per odor for all cells by projection target. This will better illustrate if all cells are responding to the same odor or a different set of odors.	Thank you for this comment and suggestion. We agree with the reviewer that this figure does not accurately reflect the diverse responses across odorants. As FOVs for imaging were located at different points on the dorsal surface of the olfactory bulb we would, however, like to point out that talking about odor specificity to particular odorants is difficult within our dataset since odorant responses are differentially mapped across the dorsal surface of the olfactory bulb, therefore, we mapped specificity always in relation to the odorant eliciting the strongest response (odor rank 1) instead of absolute responses per odorant. Figure 3B was designed this way to show that maximum response strength does not directly relate to the breadth of tuning.<br><br>Due to this and further comments from other reviewers, we have, however, commented on our set of odorants:<br>Line 828-831<br>"In this study, our odor set was limited and primarily comprised esters that have been shown to exhibit robust activation on the dorsal OB surface (Uchida et al. 2000; Wachowiak et al. 2013). Even with this limited set of chemicals, significant differences in the selectivity of AON- and APC-projecting cells were readily detectable."
15.	Likewise, the metrics used to quantify concentration-tuning in Figure 4, which also rely on quantifying the fraction of responsive cells suffer from similar concerns. As the authors note, the fraction of responsive AON-traced cells is lower than the fraction of responsive APC-traced cells at every concentration. This fact obscures the effect of concentration-dependence on the fraction of responsive cells in Figure 4E. Additional analyses (e.g. mutual information, concentration decoding) should be included to supplement this analysis.	We thank the reviewer for this helpful suggestion. We have now integrated a figure on mutual information into Figure 7 (7E) for all 4 different populations. We found that the mutual information between responses of AON MCs at the highest and lowest employed concentrations was small and did not increase when responses at the highest concentration were compared with those at the second-highest concentration. We also calculated lifetime and population sparseness for all 4 populations and found that AON MCs showed a considerably higher sparseness. The finding of low mutual information together with high sparseness in AON-traced MCs led us to change our conclusion considerably as these results suggest that AON MCs do not show a lower capacity for concentration coding but might code for concentrations differently than MCs or TCs projecting to the APC which show a marked increase in calcium signal (and therefore probably higher action potential frequency) when concentrations are increasing. We comment now on rate vs. population coding for the two respective projection targets in the results:<br>Line 678-682

		"In conclusion, APC projecting cells exhibit the strongest capacity for concentration-dependent changes in response. This suggests that APC-projecting cells convey concentration-dependent information by increasing response strength, whereas AON-projecting MCs respond more sparsely to different odor concentrations, which might point to population-based concentration coding. "
**16.**	**In Figure 4F, the response amplitude of APC-projecting MTCs is significantly greater than the response amplitude of AON-projecting MTCs over a range of concentrations. However, in Figure 3D/E, the authors report no significant difference in the response amplitude of APC vs AON-projecting MTCs. The authors should clarify the odor concentration used in earlier figures to explain this apparent disparity (in methods, results and figure legends), and comment on the significance of the difference in response amplitude at higher concentrations.**	This is a really good point, as all recordings on the selectivity of traced populations were performed at 1% VP (noted now in line 443 of the results) there is a difference in the fact that maximal amplitudes do not seem to differ in Fig 3 D/E, but there is a strong difference seen in Fig 4 F at 1%. We do not have a definitive answer to that, but we have to explain that the selectivity and sensitivity analyses are based on 2 non-overlapping datasets. Furthermore, a slight trend toward larger APC-traced neuron responses to the optimal odorant remains visible in Figure 3E, so the discrepancies might simply stem from variability in recordings and FOVs.<br><br>For further speculation on optimal concentration ranges and possible differences in adaptation across projection targets, our data are, unfortunately, insufficient; however, we will look into this interesting aspect more closely in the future.
**17.**	**The data in figure 5C indicates that a significant proportion of AON-projecting cells were found in the EPL. However, in lines 553-555, the authors write that about 25% of AON and APC-projecting cells were TCs. The authors should explain this discrepancy.**	Thanks for drawing our attention to this potentially confusing part. These two are, in fact, separate datasets. Figure 5C is based on our dual-traced dataset, in which we quantified MCs and TCs of different projection targets in histology using differential indicator expression and found that 80.6% of AON-traced cells in EPL correspond to TCs. This proportion matches well with the literature (Igarashi et al. 2012). In the imaging dataset, we used the same indicator for AON- and APC-traced cell populations in separate animals, to avoid kinetic differences across calcium indicators. Here, we recorded similar numbers of AON- and APC-projecting populations of TCs and MCs (57 TCs and 160 MCs in the AON-projecting population, and 64 TCs and 175 MCs in the APC-projecting population). Imaging experiments were performed without this separation and cell types were separated later retrospectively by depth and morphology by a researcher blind to the projection target. We suspect that, due to their larger size, we have a recording bias toward MCs, which is unphysiological for the AON. However, it enabled us to compare

		these two populations and the differences between differentially projecting cell populations.
18.	**The deep learning model that the authors use in Figure 5E is not particularly informative given its black-box nature. As the authors note, “the predictive features of this model are not easily extractable”. Given that there are no significant differences between the population averaged calcium traces of TCs or MCs with different projection targets (Figure 5D), identifying the features that drive this above-chance decoding is particularly important. We encourage the authors to utilize another approach to estimate the features distinguishing the different output streams. For instance, the SVM that the authors use in Figure 8A, which explicitly includes features extracted from activity traces could be more informative.**	Thank you for your comment. We agree that the deep learning model we used in the former Figure 5E has its drawbacks. To estimate the features that distinguish the different output streams, we used the SVM-based model shown in Figure 8. Also in response to a question from another reviewer, we further described feature importance/SVM analysis: We used features (amplitude, rise time, latency, and fall) extracted separately for each sniff from fitted 2-photon calcium imaging traces (see methods, Fig. 8A) comprising 36 sniff-features (4 features x 9 sniffs). To measure sniff-feature importance, we employed feature selection algorithms. A filter-type feature selection algorithm (we used a univariate feature ranking for classification using chi-square) measures feature importance based on feature characteristics, such as variance and relevance to the response (available in the MATLAB Statistics and Machine Learning Toolbox). This filter-type feature selection is uncorrelated to the training algorithm. Our data pool contained 1431 cell odor pairs (AON mitral 468, AON tufted 287, APC mitral 512, APC tufted 164). Data was down-sampled to match the number of data points in the smallest category. Down-sampling was repeated 100.000 times (bootstrapping), each followed by feature ranking. The results are depicted in Figure 8F, demonstrating that the feature “amplitude” had the highest feature importance, followed by “onset” and “risetime. Given the more informative nature of the SVM model, we agree with the reviewers that the additional benefit of providing the deep learning model is limited and therefore removed it in the modified version of the manuscript.
19.	**The respiration-modulation analysis appears to use different response criteria for classifying odor-modulated cell odor pairs than the criteria for classifying odor-responsive cell odor-pairs used elsewhere in the paper. The authors should please clarify the rationale for this discrepancy**	Thank you for pointing this out. Since we could not find clear criteria for sniff modulated vs. not sniff modulated activity in the literature, we came up with our own classification criterion for our fitted optical data: Traces were classified as significantly “sniff modulated” if two criteria were met: 1) Fluorescence trace fluctuations were considered as odor modulated if the maximum amplitude during odor presentation was 1.5 times higher than in the pre-odor period. 2) Fluorescence trace fluctuations were considered as sniff modulated (as opposed to a tonic response) if the relative sniff amplitude, defined as the signal amplitude following the sniff divided by the amplitude before the sniff, was less than 1.1. These empirically determined values enabled a clear separation between sniff-modulated and tonic responses. We

		employed 2SD to evaluate general odor responsiveness (independent of sniff modulation).
**20.**	**In Figure 8C, the authors show that AON-traced TCs display the greatest sniff modulation during odor presentation. However, as shown in Figure 8B, AON-traced TCs also display this greater sniff modulation during baseline, likely driven by stronger sniff modulation in TCs vs MCs overall. The authors should comment on the extent to which this effect is specific to AON-traced TCs during odor presentation, and provide additional analyses to support this conclusion.**	As described in the manuscript, we fitted traces to have a completely objective measure of sniff modulation with the algorithm automatically thresholding and detecting events. We included this objective measurement of detectable "sniffs" as we feel it is very interesting that a higher number of events can be detected exclusively in the AON projecting potion of the TCs while the number of detected breathing related events during the odor period rises most prominently in the APC projecting TC population pointing out differing properties between TCs traced from different cortical areas and underlining the argument of output streams with different properties. We have elaborated on the mechanical properties of TCs in this respect in the discussion as<br>Line 824-826<br>"Our data furthermore show that TCs have more detectable sniff-triggered transients, suggesting that they might also convey mechanical signals that OB output neurons encode, stemming from blood pressure pulsations (Jammal Salameh et al. 2024) and airflow through the nose (Mahajan et al. 2025)."
**21.**	**In the discussion, the authors argue that "although only a minority of the TC population, the APC-projecting TCs convey the predominant part of information on odor identity and concentration to the olfactory cortex". This is a substantial claim, and difficult to support without direct recordings in APC. Additionally, while the authors propose that APC-projecting TCs may convey more odor identity information than APC-projecting MCs, this is not directly shown in the analysis.**	Thank you for raising this concern. We have now amended the manuscript to include lifetime sparseness calculations for the different cell types and projection targets (Fig 6G), providing an additional measure of their selectivity. This showed that APC TCs had a slightly higher (but not significantly) lifetime sparseness than APC MCs and AON MCs, and a significantly higher lifetime sparseness than AON TCs. Additionally, APC TCs responded less promiscuously than AON TCs and responded to a panel of different odorants with more strongly differing response amplitudes than APC MCs. We therefore still believe that our assessment that APC TCs have the best identity-coding capabilities is justified, which is what we intended to convey. However, as the reviewers correctly noted, without direct measurements, this is all just circumstantial evidence, and we have therefore removed the argument from the discussion.
**22.**	**The authors should cite Koldaeva et al., 2021 as a reference for**	Done (see line 765)

	**distinguishing between the activity of mitral and tufted cells**	
23.	**To improve clarity, the authors should consider rephrasing “most unselective” to “broadest response” instead**	Thanks for your suggestion, it was rephrased.
**24.**	**Virus concentrations need to be clearly indicated in methods**	Thanks for drawing attention to this point. Injection volume is stated in the methods section at lines 147/148 as the following:<br><br>“Virus (0.5 µl, with titers in the range of 2.5 – 4.2 x 10^{12} gc/ml) was delivered through a 26 or 30-gauge metal needle at a rate of 0.1 µl/min.”
25.	**Given that mitral and tufted cells generally are more active under anesthesia, the justification of dF instead of dF/F0 is not convincing. The choice to use dF (instead of normalizing it) makes the comparison of amplitudes between projection-specific MTCs difficult to assess (between GECIs, mice, cells at different imaging depths due to nonlinear excitation through the tissue). At a minimum, the authors should provide odor-evoked z-score or dF/F responses or justify the use of dF (e.g. provide examples of low baseline responses), which may be prone to artifacts from imaging depth, relative calcium concentration, etc. An additional experiment where GCaMP and jrCaMP are expressed by AON and APC-projecting cells within the same animal may serve as a more direct comparison of their response properties without the animal-dependent artifacts.**	We thank you for your concerns regarding the comparability of the displayed amplitudes and metrics, and we understand the necessity of normalization. We previously made the argument that dF is a valid way of reporting Ca-Imaging signals, as seen in (Ingram et al. 2024), and that low baseline F values would distort values if used for normalization. Please allow us to expand this argument.<br><br>Normalization by baseline division was developed to account for differences in loading/expression, as cells with low Ca++-indicator levels can only show small-amplitude changes. We would make the argument that the current generation of calcium indicators is designed to show the highest signal-to-noise possible, which means they have a very low baseline fluorescence that is more dependent on the basic activity of a neuron than expression, and can detect single action potentials (Chen et al. 2013) which means that changes in fluorescence accurately reflect changes in neuronal firing. We would therefore prefer to show dF values rather than the, in our opinion, outdated dF/F measure, though we will change all analyses to dF/F if the reviewers insist.

26.	**Correction: line 355 “sites” instead of “sides”**	Thanks for the correction; this has been changed.
**27.**	**Citation needed: line 368**	Thanks for your comment. The following was added<br>Line 369:<br>“Since AON and APC serve different functions in odor processing (Bolding and Franks 2017; Lei et al. 2006), we speculated that differences between AON- and APC-projecting MTCs might already be observable using epifluorescence OB population imaging.”
**28.**	**The set of odors hardly allows the authors to distinguish odor selectivity across a broad range of odorant classes. The authors should mention this as a potential caveat when interpreting the results about odor selectivity across processing streams.**	We have added a sentence in the discussion.<br>Line 828-831<br>“In this study, our odor set was limited and primarily comprised esters that have been shown to exhibit robust activation on the dorsal OB surface (Uchida et al. 2000; Wachowiak et al. 2013). Even with this limited set of chemicals, significant differences in the selectivity of AON- and APC-projecting cells were readily detectable.”
29.	**The authors should convert their “selectivity” measure into lifetime sparseness so that it is more easily comparable to other studies on the OB and olfactory cortex.**	Done. The additional plots can be seen in 3F, 4H, 6G und 7F.
**30.**	**It’s unclear in the tuning curve calculations if the authors accounted for differences in the number of imaged mitral and tufted cells by regional projections (see lines 624-626). Since it’s unclear if the tuning curves would be dominated by the most numerous cell type, the authors should provide additional evidence or account for this potential artifact.**	We are sorry for this misunderstanding. In fact, both AON and APC projecting populations in Figure 1-4 have similar numbers of TCs and MCs. Imaging experiments were performed without this separation, and, in a retrospective analysis, cell types were subsequently separated by depth and morphology by a researcher blind to the projection target. As a result, we found 57 TCs and 160 MCs in the AON-projecting cell population, and 64 TCs and 175 MCs in the APC-projecting cell population. Thus, likely due to their larger size, we observe a recording bias toward MCs, which is unphysiological for the AON. However, it enables us to compare these two populations and the differences between them, which are derived from the properties of the projection-target-defined cells rather than from discrepancies between numbers of TCs and MCs within these populations.<br>Please also see the response to major point 4.

**31.**	**Lines 641-642 – is it possible that APC-projecting TCs respond to a narrow range of concentrations but that their sensitivity is higher? Did the authors use concentrations lower than 0.1%?**	This is a very good point. The odor concentrations mentioned here are within the range common for experiments in anesthetized mice i.e. (Wachowiak et al. 2013; Ogg et al. 2018; Zak et al. 2024). We cannot, however, exclude the possibility that cell populations have increased sensitivity outside the applied range.
**32.**	**It's unclear if the odor-evoked responses were aligned to the inhalation. The authors should clarify and include additional newly aligned odor-responses (as latencies, and other metrics may shift the results).**	Thanks for bringing this to our attention. In all experiments included in this manuscript, animals underwent a double tracheotomy procedure, which enabled an artificial sniffing paradigm that overcomes issues arising from the natural inhalation rhythm (Wachowiak and Cohen 2001; Spors et al. 2006; Carey and Wachowiak 2011; Wachowiak et al. 2013). This double tracheotomy enables artificial sniffing, providing complete control over orthonasal odor stimulation. This method increases the reproducibility of results, reducing noise, and overcomes optical signal issues caused by natural breathing. A key advantage is that odor onset and inhalation onset/frequency are precisely synchronized, and this holds across odor presentations and animals.<br>In order to make this clearer, we have added the following to our manuscript:<br>Line 154-157<br>"Odorants were presented for 4 seconds, with odor onset preceding the inhalation of the artificial sniff. This way, odor onset and inhalation onset/frequency are precisely timed, allowing precise averaging across animals without the need for e.g., "time warping"."
**33.**	**Lines 787-788 - the authors should highlight how the AON-TCs fits well with prior literature**	Done, this can be found in the discussion now<br>Line 814-826<br>"One reason for the stronger diversity in TC response properties might be that, according to our dual tracing results, these contain a lower number of neurons that have collaterals in both areas, but also corresponds well with previous reports that demonstrated that TCs, in general, convey stronger stimulus-specific information (Chae et al. 2022). TCs have furthermore been shown to form a more heterogeneous cell population (Nagayama et al. 2014), receive stronger afferent input than mitral cells (Gire et al. 2012), show stronger excitability and more irregular firing patterns (Burton and Urban 2014), and respond with a more stable timing in relation to the sniff onset (Fukunaga et al. 2012). All these findings support the idea that TCs are responsible for the rapid distribution of information to olfactory cortical centers.

		Their wide range of firing patterns observed in *in vitro* recordings supports the notion that cells in this population have inherently different response properties, possibly due to differing projection targets. Our data furthermore show that TCs have more detectable sniff-triggered transients, suggesting that they might also convey mechanical signals that OB output neurons encode, stemming from blood pressure pulsations (Jammal Salameh et al. 2024) and airflow through the nose (Mahajan et al. 2025).“
**34.**	**The discussion could benefit from a more detailed speculation on the role of these parallel processing streams in the behaving animal.**	Thanks for this comment, as multiple reviewers have requested this, we have added a separate section in the discussion titled “*Differential output streams functions”*<br>Line 834 – 842<br>„One possible interpretation is that the AON, with its strong, fast, and sniff-modulated inputs, serves as a first responder in odor detection in animals where specificity could be sacrificed for reliability and speed. The first sniffs of an incoming odor might then trigger responses in the animal, such as an increase in sniffing (i.e., sampling rate), if warranted. This would also align with the findings of Schiltz et al., who report that AON neurons exhibit poor odor specificity but respond strongly to novel stimuli (Schiltz et al. 2025), and with the hypothesis that the AON might function as a tool for olfactory navigation (Brunert et al. 2023). The APC that receives more specific odor-related information would then be responsible for fine discrimination and valence coding, tasks that require such information.”

Publication bibliography

Ackels, T.; Jordan, R.; Schaefer, A. T.; Fukunaga, I. (2020): Respiration-Locking of Olfactory Receptor and Projection Neurons in the Mouse Olfactory Bulb and Its Modulation by Brain State. In *Frontiers in cellular neuroscience* 14, p. 220. DOI: 10.3389/fncel.2020.00220.

Amrutkar, Ankit; Kampa, Björn; Schulz, Volkmar; Stegmaier, Johannes; Rothermel, Markus; Merhof, Dorit (2025): Towards Robust and Generalizable Gerchberg Saxton based Physics Inspired Neural Networks for Computer Generated Holography: A Sensitivity Analysis Framework. Available online at https://arxiv.org/pdf/2505.00220.

Banerjee, A.; Marbach, F.; Anselmi, F.; Koh, M. S.; Davis, M. B.; Da Garcia Silva, P. et al. (2015): An Interglomerular Circuit Gates Glomerular Output and Implements Gain Control in the Mouse Olfactory Bulb. In *Neuron* 87 (1), pp. 193–207. DOI: 10.1016/j.neuron.2015.06.019.

Boehm, Erik; Brunert, Daniela; Rothermel, Markus (2020): Input dependent modulation of olfactory bulb activity by GABAergic basal forebrain projections. In *bioRxiv*, 2020.03.29.014191. DOI: 10.1101/2020.03.29.014191.

Bolding, K. A.; Franks, K. M. (2017): Complementary codes for odor identity and intensity in olfactory cortex. In *eLife* 6. DOI: 10.7554/eLife.22630.

Bozza, T.; McGann, J. P.; Mombaerts, P.; Wachowiak, M. (2004): In vivo imaging of neuronal activity by targeted expression of a genetically encoded probe in the mouse. In *Neuron* 42 (1), pp. 9–21. DOI: 10.1016/s0896-6273(04)00144-8.

Brunert, D.; Medinaceli Quintela, R.; Rothermel, M. (2023): The anterior olfactory nucleus revisited - An emerging role for neuropathological conditions? In *Progress in neurobiology* 228, p. 102486. DOI: 10.1016/j.pneurobio.2023.102486.

Burton, S. D.; Urban, N. N. (2014): Greater excitability and firing irregularity of tufted cells underlies distinct afferent-evoked activity of olfactory bulb mitral and tufted cells. In *The Journal of physiology*. DOI: 10.1113/jphysiol.2013.269886.

Carey, R. M.; Wachowiak, M. (2011): Effect of sniffing on the temporal structure of mitral/tufted cell output from the olfactory bulb. In *The Journal of neuroscience : the official journal of the Society for Neuroscience* 31 (29), pp. 10615–10626.

Chae, H.; Banerjee, A.; Dussauze, M.; Albeanu, D. F. (2022): Long-range functional loops in the mouse olfactory system and their roles in computing odor identity. In *Neuron*. DOI: 10.1016/j.neuron.2022.09.005.

Chen, T.-W.; Wardill, T. J.; Sun, Y.; Pulver, S. R.; Renninger, S. L.; Baohan, A. et al. (2013): Ultra-sensitive fluorescent proteins for imaging neuronal activity. In *Nature* in press.

Chen, Yushu; Chen, Xiaoyin; Baserdem, Batuhan; Zhan, Huiqing; Li, Yan; Davis, Martin B. et al. (2022): High-throughput sequencing of single neuron projections reveals spatial organization in the olfactory cortex. In *Cell* 185 (22), 4117-4134.e28. DOI: 10.1016/j.cell.2022.09.038.

Cheung, M. C.; Carey, R. M.; Wachowiak, M. (2009): A method for generating natural and user-defined sniffing patterns in anesthetized or reduced preparations. In *Chemical senses* 34 (1), pp. 63–76.

Congiu, Mauro; Mondoloni, Sarah; Zouridis, Ioannis S.; Schmors, Lisa; Lecca, Salvatore; Lalive, Arnaud L. et al. (2023): Plasticity of neuronal dynamics in the lateral habenula for cue-punishment associative learning. In *Molecular psychiatry* 28 (12), pp. 5118–5127. DOI: 10.1038/s41380-023-02155-3.

Economo, M. N.; Hansen, K. R.; Wachowiak, M. (2016): Control of Mitral/Tufted Cell Output by Selective Inhibition among Olfactory Bulb Glomeruli. In *Neuron* 91 (2), pp. 397–411. DOI: 10.1016/j.neuron.2016.06.001.

Fukunaga, Izumi; Berning, Manuel; Kollo, Mihaly; Schmaltz, Anja; Schaefer, Andreas T. (2012): Two Distinct Channels of Olfactory Bulb Output. In *Neuron* 75 (2), pp. 320–329. DOI: 10.1016/j.neuron.2012.05.017.

Gire, David H.; Franks, Kevin M.; Zak, Joseph D.; Tanaka, Kenji F.; Whitesell, Jennifer D.; Mulligan, Abigail A. et al. (2012): Mitral Cells in the Olfactory Bulb Are Mainly Excited through a Multistep Signaling Path. In *The Journal of Neuroscience* 32 (9), pp. 2964–2975. DOI: 10.1523/jneurosci.5580-11.2012.

Grobman, M.; Dalal, T.; Lavian, H.; Shmuel, R.; Belelovsky, K.; Xu, F. Q. et al. (2018): A Mirror-Symmetric Excitatory Link Coordinates Odor Maps across Olfactory Bulbs and Enables Odor Perceptual Unity. In *Neuron* 99 (4), 800-+. DOI: 10.1016/j.neuron.2018.07.012.

Hirata, T.; Fujisawa, H. (1999): Environmental control of collateral branching and target invasion of mitral cell axons during development. In *J. Neurobiol.* 38 (1), pp. 93–104.

Igarashi, K. M.; Ieki, N.; An, M.; Yamaguchi, Y.; Nagayama, S.; Kobayakawa, K. et al. (2012): Parallel mitral and tufted cell pathways route distinct odor information to different targets in the olfactory cortex. In *The Journal of neuroscience : the official journal of the Society for Neuroscience* 32 (23), pp. 7970–7985. DOI: 10.1523/JNEUROSCI.0154-12.2012.

Ingram, Sonia; Chisholm, Kim I.; Wang, Feng; Koninck, Yves de; Denk, Franziska; Goodwin, George L. (2024): Assessing spontaneous sensory neuron activity using in vivo calcium imaging. In *Pain* 165 (5), pp. 1131–1141. DOI: 10.1097/j.pain.0000000000003116.

Jammal Salameh, Luna; Bitzenhofer, Sebastian H.; Hanganu-Opatz, Ileana L.; Dutschmann, Mathias; Egger, Veronica (2024): Blood pressure pulsations modulate central neuronal activity via mechanosensitive ion channels. In *Science* 383 (6682), eadk8511. DOI: 10.1126/science.adk8511.

Lei, H.; Mooney, R.; Katz, L. C. (2006): Synaptic integration of olfactory information in mouse anterior olfactory nucleus. In *The Journal of neuroscience : the official journal of the Society for Neuroscience* 26 (46), pp. 12023–12032. DOI: 10.1523/JNEUROSCI.2598-06.2006.

Leong, Lee Min; Wharton, David; Subramanian, Narayan; Karamched, Bhargav; Bertram, Richard; Storace, Douglas A. (2025): Evidence that interglomerular inhibition generates non-monotonic concentration-response relationships in mitral/tufted glomeruli in the mouse olfactory bulb. In *bioRxiv*. DOI: 10.1101/2025.02.28.640652.

Mahajan, Sarang; Das, Susobhan; Tamboli, Suhel; Pandey, Sanyukta; Bhattacharjee, Anindya S.; Pardasani, Meenakshi et al. (2025): Mouse olfactory system acts as anemo-detector and anemo-discriminator. In *Science advances* 11 (41), eadq8390. DOI: 10.1126/sciadv.adq8390.

Medinaceli Quintela, R.; Bauer, J.; Wallhorn, L.; Le, K.; Brunert, D.; Rothermel, M. (2020): Dynamic Impairment of Olfactory Behavior and Signaling Mediated by an Olfactory Corticofugal System. In *The Journal of neuroscience : the official journal of the Society for Neuroscience* 40 (38), pp. 7269–7285. DOI: 10.1523/JNEUROSCI.2667-19.2020.

Nagayama, S.; Homma, R.; Imamura, F. (2014): Neuronal organization of olfactory bulb circuits. In *Frontiers in neural circuits* 8, p. 98. DOI: 10.3389/fncir.2014.00098.

Ogg, M. C.; Ross, J. M.; Bendahmane, M.; Fletcher, M. L. (2018): Olfactory bulb acetylcholine release dishabituates odor responses and reinstates odor investigation. In *Nature communications* 9 (1), p. 1868. DOI: 10.1038/s41467-018-04371-w.

Otazu, G. H.; Chae, H.; Davis, M. B.; Albeanu, D. F. (2015): Cortical Feedback Decorrelates Olfactory Bulb Output in Awake Mice. In *Neuron* 86 (6), pp. 1461–1477. DOI: 10.1016/j.neuron.2015.05.023.

Parabucki, Ana; Bizer, Alexander; Morris, Genela; Munoz, Antonio E.; Bala, Avinash D. S.; Smear, Matthew; Shusterman, Roman (2019): Odor Concentration Change Coding in the Olfactory Bulb. In *eNeuro* 6 (1). DOI: 10.1523/ENEURO.0396-18.2019.

Rothermel, M.; Brunert, D.; Zabawa, C.; Diaz-Quesada, M.; Wachowiak, M. (2013): Transgene expression in target-defined neuron populations mediated by retrograde infection with adeno-associated viral vectors. In *The Journal of neuroscience : the*

official journal of the Society for Neuroscience 33 (38), pp. 15195–15206. DOI: 10.1523/JNEUROSCI.1618-13.2013.

Rothermel, M.; Wachowiak, M. (2014): Functional imaging of cortical feedback projections to the olfactory bulb. In *Frontiers in neural circuits* 8, p. 73. DOI: 10.3389/fncir.2014.00073.

Schiltz, Eleonore; Broux, Martijn; Aydin, Cagatay; Goncalves, Pedro; Haesler, Sebastian (2025): Experience shapes the transformation of olfactory representations along the cortico-hippocampal pathway.

Spors, H.; Wachowiak, M.; Cohen, L. B.; Friedrich, R. W. (2006): Temporal dynamics and latency patterns of receptor neuron input to the olfactory bulb. In *The Journal of neuroscience : the official journal of the Society for Neuroscience* 26 (4), pp. 1247–1259. DOI: 10.1523/JNEUROSCI.3100-05.2006.

Uchida, N.; Takahashi, Y. K.; Tanifuji, M.; Mori, K. (2000): Odor maps in the mammalian olfactory bulb: domain organization and odorant structural features. In *Nature neuroscience* 3 (10), pp. 1035–1043.

Vanwalleghem, Gilles; Constantin, Lena; Scott, Ethan K. (2020): Calcium Imaging and the Curse of Negativity. In *Frontiers in Neural Circuits* 14, p. 607391. DOI: 10.3389/fncir.2020.607391.

Verhagen, J. V.; Wesson, D. W.; Netoff, T. I.; White, J. A.; Wachowiak, M. (2007): Sniffing controls an adaptive filter of sensory input to the olfactory bulb. In *Nature neuroscience* 10 (5), pp. 631–639. DOI: 10.1038/nn1892.

Wachowiak, M.; Cohen, L. B. (2001): Representation of odorants by receptor neuron input to the mouse olfactory bulb. In *Neuron* 32 (4), pp. 723–735. DOI: 10.1016/s0896-6273(01)00506-2.

Wachowiak, M.; Economo, M. N.; Diaz-Quesada, M.; Brunert, D.; Wesson, D. W.; White, J. A.; Rothermel, M. (2013): Optical dissection of odor information processing in vivo using GCaMPs expressed in specified cell types of the olfactory bulb. In *The Journal of neuroscience : the official journal of the Society for Neuroscience* 33 (12), pp. 5285–5300. DOI: 10.1523/JNEUROSCI.4824-12.2013.

Zak, Joseph D.; Reddy, Gautam; Konanur, Vaibhav; Murthy, Venkatesh N. (2024): Distinct information conveyed to the olfactory bulb by feedforward input from the nose and feedback from the cortex. In *Nat Commun* 15 (1), p. 3268. DOI: 10.1038/s41467-024-47366-6.

Reviewer 4

1.	**One of our initial concerns focused on the question of how the 30-50% of MTCs that collateralize to both AON and APC contribute to odor coding. The addition of the new supplemental figure (S2) helps to clarify this point by comparing odor tuning in dual-labeled vs single-labeled cells. It would be informative to also generate a similar plot showing the response properties of single-labeled vs dual-labeled cells separated by cell type (MCs vs TCs) and in response to the concentration series if this data is available. Finally, the authors should clarify which analyses do and do not contain dual-projecting MTCs**.	Thank you for this comment. We have now created a figure similar to Supp. Fig. 2 to separate this dataset with dually traced cells into mitral and tufted cells (Supp Fig. 3, line 640 and 1066). As you can see in both populations, MCs and TCs, the cumulative probability plot shows stronger differences between AON and APC traced cells than the original figure Fig. 6f. That said, this dataset is limited and both lifetime and population sparseness are only significant for the strong differences between AON and APC traced cells in TCs while p-values in MCs indicate a clear trend but do not reach significance levels.<br>The dataset for the concentration-dependent coding is even more limited and, therefore, not suitable for this type of analysis.<br>Due to the limited nature of this dataset, all figures in the main body of the text are generated using potentially dual-projecting neurons. To make this clearer, we have included the following sentence (line 589).<br>“Here, we used our functional 2-photon microscopy dataset, obtained with the initial approach (Fig. 1A), separated into MCs and TCs, though it contained potentially dually projecting cells, as this dataset was much larger and thus more representative.”
2.	**As we noted in our initial review, differences in the tuning properties of MCs and TCs are central to explaining the differences in target-specific MTC activity, as this paper shows in later figures. The importance of this distinction argues for the introduction of these cell-type specific differences earlier in the paper. Since this information is critical to the paper's argument, and (if we understand correctly), available for all of the data present in Figures 1-4, we would argue (in agreement with reviewer 2) that the repeated presentation of grouped vs separated datasets reads as redundant, and that the paper would be made more clear by separating the data by cell type from the start.**	Thank you for raising this point. However, we would like to politely disagree. Through the given narrative, we want to make clear: First, how we approached this project, i.e., first analyzing data purely according to the projection target and only later sorting out the different cell types. Second, this narrative and the fact that AON and APC projecting datasets contain equal numbers of MCs and TCs are meant to convey the message that it is **not** only the cell types that carry the differences between projection targets. This is later supported by the SVM, which shows superior performance in classifying by the projection target compared to cell type.<br>We also made this argument in the first round of the revision. As it was accepted by reviewers 1 and 2, we would like to refrain from changing the manuscript.
3.	**We note the addition of lifetime and population sparseness, and mutual**	We also feel that the suggested analysis made the paper a lot stronger. Right now, the SVM decoder

	**information metrics, which we agree have significantly improved the paper. However, we feel that additional value would be added by comparing the performance of population decoders trained to decode odor identity and concentration separately using these two cell populations (AON vs APC projecting). Since neurons in APC/AON receive convergent input from many MTCs, this population decoding could better approximate the information available to these downstream cortical circuits. It would be informative to compare the overall decoding performance as well as the distribution of decoding errors (e.g. bias towards high or low concentrations) between the two populations.**	contains only identity data, not intensity (concentration) data, as the former dataset was larger and thus more representative.<br>Though we agree that exploring the concentration coding in more detail with special attention to decoding errors at different concentrations would be most interesting, we feel that a more detailed analysis of concentration coding and decoding to see at which concentrations the respective areas perform best is beyond the scope of what we aimed to accomplish with this paper.
4.	**The authors indicate that they classify cells by lamination, cell body size, and other morphological features. Given the importance of this classification, representative images of the different cell types (mitral and tufted cells) and the features with which they classified them would be helpful.**	Thank you for your comment. As we have stated in answer to point 2, we do not think that the separation into mitral and tufted cells is the key point of the paper.<br>We can, however, understand the reviewer's worry about this separation. As we could not find any strict reference values, we also did not adhere strictly to values.<br>MCs were 20-30 µm in diameter, with a wide, round or triangular shape, while cells classified as TCs were between 15 and 25 µm and often had a drop-like shape. We believe a better argument for the classification is the location within the OB layers, as identified in high-resolution z-stacks recorded after the imaging session within the FOV. Traced cells showed rich dendritic tufts in the glomerular layer; deeper, you would find a rich neuropile consisting of dendrites with cell bodies (TCs) embedded within this layer, until, deeper still, a consistent layer of cell bodies (MCs) would be visible.<br>To demonstrate this, we have attached a GIF for the reviewer that simulates scanning through the different OB layers. This particular example is from an APC-traced animal where we imaged from the mitral cell layer at 292µm depth.
5.	**The difference in image quality between mitral and tufted cells in figure 6a illustrates the potential confounds of comparing neural activity of mitral and tufted cells without normalization, given that the amplitudes may simply reflect**	Though we appreciate the reviewers' concerns about the confounds of cells imaged at different imaging depths, we are confident that, even if that were a consistent problem in our dataset, it would not explain the differences between AON and APC-projecting cells observed in both tufted and mitral

	**imaging quality at different depths (see e.g. 6d and 6e, where MCs have lower response amplitudes than TCs regardless of projection target). The authors should acknowledge this caveat and describe how they account for the potential confound of imaging quality at different imaging depths when comparing MCs and TCs. We would further urge the authors to consider dF/F normalization - which is straightforward - or to otherwise demonstrate that the differences observed between MC/TC responses are not due solely to depth.**	cells. To alleviate some of the concerns, we have calculated the signal-to-noise ratio, i.e., the average of the signal during odor stimulation (4s)/the average of the baseline (4s) for all significantly responding cells. You can see the result below: 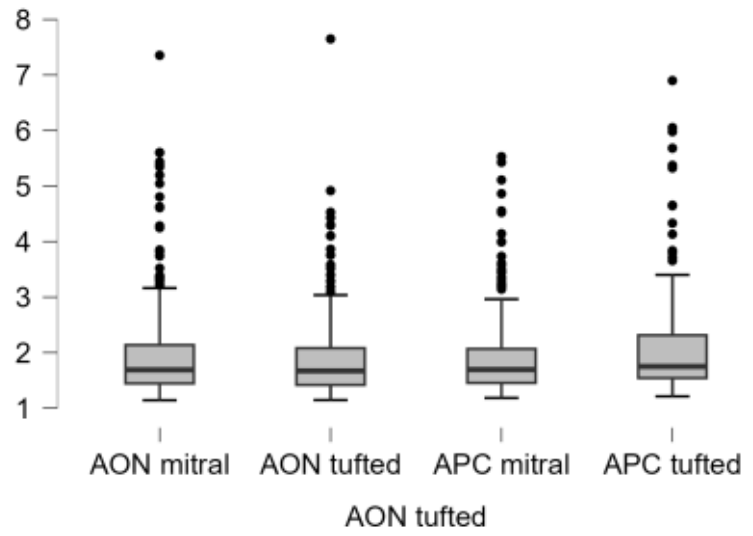 Statistical check revealed no difference between mitral and tufted cells.

Minor Comments

1.	**We appreciate the addition of information to the legends (including # of cells per projection area, information about concentration in earlier figures) which we feel have improved the paper's clarity. Could the authors also please clarify which odorant(s) were used in the concentration series experiments?**	Done
2.	**The following sentence is unclear: “Cumulative probability and sparseness plots showed double-labeled cells located between AON or APC-projecting cells. Interestingly, significance levels increased for both lifetime (ANOVA, post hoc test, p<0.001) and population sparseness (ANOVA, post hoc test, p = 0.001), suggesting that, although not all OB output neurons project to a single cortical area, those that do show a highly significant difference in their coding properties.**	The reviewer is right, that was phrased poorly and has now been changed (line 578)<br>“Cumulative probability and sparseness plots showed that double-labeled cells had properties unlike those of AON- or APC-projecting cells but fell right between those of singly projecting cells. Interestingly, significance levels for difference between AON and APC projecting MTCs became larger through removal of the dually projecting cells for both lifetime (ANOVA, post hoc test, p<0.001) and population sparseness (ANOVA, post hoc test, p = 0.001), suggesting that, although not all OB output neurons project to a single cortical area, those that do show a highly significant difference in their coding properties.”
3.	**The following sentence is unclear: “For both AON- and APC-traced OB output streams, we found that about a fourth of the identified cells were TCs and that on average more TCs showed at least one significant odor response than MCs for both traced populations”**	This sentence now reads (line 595)<br>“Separating the differentially traced populations we found that both contained an equal ratio of tufted to mitral cells, i.e.. in both populations, only about a quarter of the cells were tufted cells. Those tufted cells were more responsive to the tested odor panel, as more TCs showed at least one significant odor response than MCs for both traced populations”

4.	**It's unclear if figures 6-7 use the dual injection strategy or if it uses the approach in figure 1 (diff mice with the same calcium indicator). The author could clarify this in their figures by including a schematic and/or more detail in the figure legends.**	The dataset in figures 1, 6, and 7 is the same. We did not perform all calculations on datasets cleared of dually projecting cells, as the dataset was too small. To make this clearer, we have included a sentence (line 589):<br>"Here, we used our functional 2-photon microscopy dataset, obtained with the initial approach (Fig. 1A), separated into MCs and TCs, though it contained potentially dually projecting cells, as this dataset was much larger and thus more representative."<br><br>Although the data could not be cleared of dual-projecting cells, we hope the reviewer agrees that the differences between the differentially traced populations are clearly visible.
5.	**Row 316 should say 'sites'**	done
6.	**This sentence in the abstract is unclear: However, differences in target outweigh pure cell-identity differences, as demonstrated by support vector machine classifier analysis. Please clarify.**	This sentence has been modified.
